# STAT6-targeting antisense oligonucleotides against solitary fibrous tumor

**DOI:** 10.1016/j.omtn.2024.102154

**Published:** 2024-02-15

**Authors:** Yi Li, Jose L. Mondaza-Hernandez, David S. Moura, Alexey S. Revenko, Angelica Tolentino, John T. Nguyen, Nam Tran, Clark A. Meyer, Jose Merino-Garcia, Rafael Ramos, Davide Di Lernia, Javier Martin-Broto, Heather N. Hayenga, Leonidas Bleris

**Affiliations:** 1 Department of Bioengineering, University of Texas at Dallas, Richardson, TX 75080, USA; 2 Center for Systems Biology, University of Texas at Dallas, Richardson, TX 75080, USA; 3 Health Research Institute Fundacion Jimenez Diaz, Universidad Autonoma de Madrid (IIS/FJD-UAM), 28049 Madrid, Spain; 4 University Hospital General de Villalba, 28400 Madrid, Spain; 5 Department of Antisense Drug Discovery, Ionis Pharmaceuticals, Inc, Carlsbad, CA 92010, USA; 6 Neurosurgical Oncology, Department of Neuro-Oncology, H. Lee Moffitt Cancer Center and Research Institute, Tampa, FL 33612, USA; 7 Pathology Department, University Hospital Fundacion Jimenez Diaz, Universidad Autonoma, Av. Reyes Catolicos 2, 28040 Madrid, Spain; 8 Pathology Department, University Hospital Son Espases, 07120 Palma de Mallorca, Spain; 9 Medical Oncology Department, University Hospital Fundacion Jimenez Diaz, 28040 Madrid, Spain; 10 Department of Biological Sciences, University of Texas at Dallas, Richardson, TX 75080, USA

**Keywords:** MT: Oligonucleotides: Therapies and Applications, solitary fibrous tumor, SFT, antisense oligonucleotides, ASOs, patient-derived xenograft, PDX, gene fusion, sarcoma, hemangiopericytoma

## Abstract

Solitary fibrous tumor (SFT) is a rare, non-hereditary soft tissue sarcoma thought to originate from fibroblastic mesenchymal stem cells. The etiology of SFT is thought to be due to an environmental intrachromosomal gene fusion between NGFI-A-binding protein 2 (NAB2) and signal transducer and activator protein 6 (STAT6) genes on chromosome 12, wherein the activation domain of STAT6 is fused with the DNA-binding domain of NAB2 resulting in the oncogenesis of SFT. All NAB2-STAT6 fusion variations discovered in SFTs contain the C-terminal of STAT6 transcript, and thus can serve as target site for antisense oligonucleotides (ASOs)-based therapies. Indeed, our *in vitro* studies show the STAT6 3′ untranslated region (UTR)-targeting ASO (ASO 993523) was able to reduce expression of NAB2-STAT6 fusion transcripts in multiple SFT cell models with high efficiency (half-maximal inhibitory concentration: 116–300 nM). Encouragingly, *in vivo* treatment of SFT patient-derived xenograft mouse models with ASO 993523 resulted in acceptable tolerability profiles, reduced expression of NAB2-STAT6 fusion transcripts in xenograft tissues (21.9%), and, importantly, reduced tumor growth (32.4% decrease in tumor volume compared with the untreated control). Taken together, our study established ASO 993523 as a potential agent for the treatment of SFTs.

## Graphical abstract

**Figure:**
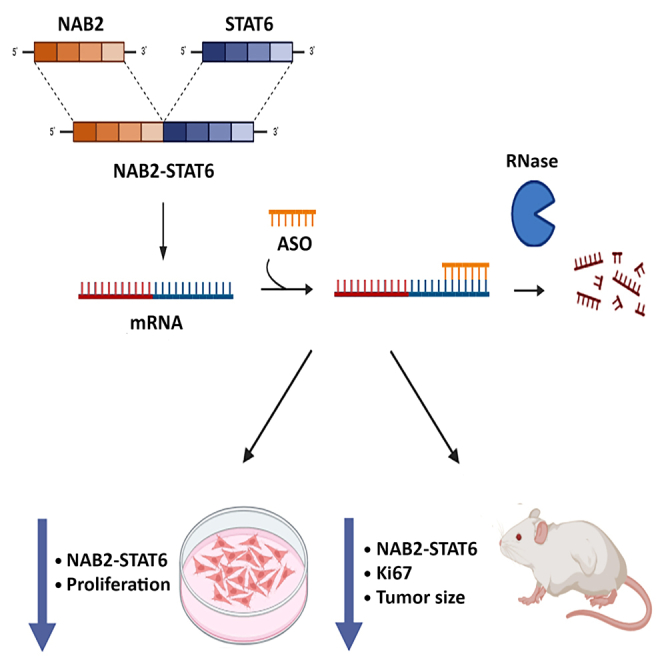

## Teaser

Hayenga and colleagues demonstrated that ASOs can efficiently downregulate the expression of NAB2-STAT6 fusion transcripts *in vitro* and *in vivo*, and, importantly, exert anti-tumor effects *in vivo* using SFT cell line models and PDX mouse models.

## Introduction

Solitary fibrous tumor (SFT) is a rare nonhereditary soft tissue sarcoma that affects approximately 1 per 1 million people per year worldwide. SFTs appear as highly vascularized solid and/or cystic tumors.^1^ They are thought to originate from mesenchymal stem cells with a fibroblastic phenotype.^2^^,^^3^^,^^4^ Anatomically, the primary site of origin is ubiquitous and can occur in most places (commonly found intracranially within the meninges and extracranially within the pleura, pelvis, retroperitoneum, kidney, liver, limbs, or pancreas). SFTs have a high rate of local recurrence (65%) and widespread metastases (33%), with a proclivity to metastasize to the bones, liver, and lungs.^5^^,^^6^^,^^7^^,^^8^ The median overall survival for SFT patients on the most effective systemic therapies currently available (pazopanib, bevacizumab and temozolomide, sunitinib, trabectedin, etc.) is 11–24 months.^9^^,^^10^^,^^11^ Therefore, a targeted and efficacious systemic treatment for SFT is highly desired.

In 2013, a defining discovery was made that all SFTs have a hallmark intrachromosomal gene fusion between NGFI-A-binding protein 2 (NAB2) and signal transducer and activator protein 6 (STAT6) on chromosome 12 (region 12q13).^12^^,^^13^ Since then, at least 12 distinct junctional breakpoints (between exons 2–7 on NAB2 and exons 2–22 on STAT6) of the NAB2-STAT6 fusion have been identified and are thought to account for pathological variation and tumor aggressiveness in SFTs.^14^^,^^15^ The exact mechanism of action remains to be elucidated. However, it has been hypothesized that due to the nuclear localization signal and DNA-binding domain in NAB2, the NAB2-STAT6 fusion is translocated to the nucleus and functions as an oncogenic transcriptional factor. Specifically, depending on the fusion breakpoint junction, the chimeric protein produced by the NAB2-STAT6 gene fusion has at least one repressor domain from NAB2 replaced by the transactivation domain of STAT6, resulting in constitutive activation of early growth response factor 1-mediated transcription and downstream oncogenesis.^12^^,^^13^^,^^16^^,^^17^ Even though the NAB2-STAT6 gene fusion alone seems to drive the cancer, therapeutic options and most importantly, targeted studies and clinical trials remain elusive.^10^

Antisense oligonucleotides (ASOs) are synthetic single-stranded nucleic acid sequences (typically 15–25 bp in length) that can regulate expressions of their mRNA targets via two major pathways: an RNase H-dependent mechanism that consists mainly of DNA or both RNA and DNA ASOs, and an RNase H-independent mechanism that mainly consists of RNA ASOs. In the RNase H-dependent mechanism, DNA-based ASOs bind to their complementary target mRNAs, and RNase H recognizes the DNA-RNA heteroduplex and subsequently catalyzes the degradation of the mRNA.^18^^,^^19^^,^^20^ Therefore, ASOs could serve as useful therapeutic agents for the treatment of diseases caused by the overexpression of certain genes. By 2021, 14 ASOs had been approved by the U.S. Food and Drug Administration that target diverse genetic and rare diseases, including Duchenne muscular dystrophy, spinal muscular atrophy, and hypercholesterolemia.^21^^,^^22^^,^^23^^,^^24^^,^^25^^,^^26^^,^^27^^,^^28^^,^^29^

In this study, we evaluate a STAT6 3′ UTR-targeting, single-stranded DNA ASOs, with the objective to downregulate the expression of all NAB2-STAT6 fusion transcripts, in an effort to exert anti-tumor benefits for SFTs. Importantly, this therapy can apply broadly to SFT patients; all NAB2-STAT6 fusion types contain the C-terminal of STAT6 transcript.

## Results

### Generation of SFT cell models

**Figure 1:**
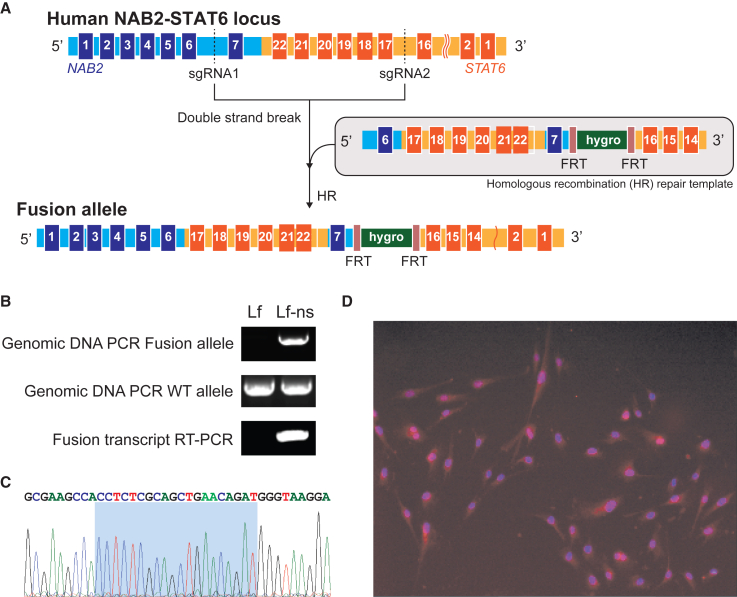
Generation of human Lf-ns cells using CRISPR (A) Schematic illustration of the homologous recombination process to create the specific fusion type. (B) Long-range genomic DNA and RNA RT-PCR assays confirmed that Lf-ns cells are heterozygous. (C) Sanger sequencing result of RT-PCR amplicons confirmed the fusion type as NAB2exon6-STAT6exon17. (D) ICC assay showed prominent nuclear expression of NAB2-STAT6 fusion protein. (Red) STAT6 expression by anti-STAT6 antibody conjugated with Alexa Fluor 594, (blue) nuclear staining by DAPI.

We previously established an SFT cell line (NS-poly) by editing the NAB2exon6-STAT6exon17 fusion into human colon cancer cells (HCT116)^30^ using the CRISPR-SpCas9 system.^31^^,^^32^^,^^33^^,^^34^^,^^35^^,^^36^ However, SFTs are believed to originate from mesenchymal stem cells with fibroblastic differentiation. Therefore, we developed a human telomerase reverse transcriptase (hTERT)-immortalized lung fibroblast-based NAB2exon6-STAT6exon17 (Lf-ns) cell line using the same CRISPR-mediated knock-in strategy as for the NS-poly cell model (Figure 1A). Next, to determine the genotype of Lf-ns cells, we harvested genomic DNA and performed long-range PCR using forward primer P1 (within exon 6 of NAB2) and reverse primer P2 (within exon 17 of STAT6) (Table S1). As shown in Figure 1B, the expected amplicon (1,194 bp) was observed in Lf-ns cells, but not in the parental immortalized Lf. Next, the genomic DNA was subjected to long-range PCRs using primers designed for wild-type alleles (forward primer P3: within exon 6 of NAB2; and reverse primer P4: within intron 6 of NAB2). The expected amplicon (1,020 bp) was observed in both Lf-ns and Lf cells. Taken together, these results indicated that our Lf-ns stable cells were heterozygous for both the genomic NAB2-STAT6 fusion and wild-type STAT6.

To confirm the presence of the fusion at the RNA-level, we harvested the total RNA from stable fibroblasts and performed RT-PCR for the fusion transcript (441 bp, forward primer P5: within exon 5 of NAB2; reverse primer P6: within exon 19 of STAT6). Consistent with results from our long-range PCRs, an expected NAB2-STAT6 fusion transcript band was observed in the Lf-ns cells, but not Lf cells (Figure 1B). The intended fusion breakpoint was further confirmed through sanger sequencing (Figure 1C) (breakpoint adjacent region between NAB2 exon 6: 5′-CCTCTCGCAG-3′ and STAT6 exon 17: 5′-CTGAACAGAT-3′, highlighted in blue). Next, we performed immunocytochemistry (ICC) on Lf-ns cells using an anti-STAT6 antibody conjugated with Alexa Fluor 594. As shown in Figure 1D, prominent nuclear expression of STAT6 fusion was observed. This result was consistent with previous reports, which show the STAT6 protein is predominately translocated into the nucleus upon fusion with NAB2.^12^

In addition to developing genetically engineered SFT cells, we procured three primary SFT cell lines. The primary cell lines were harvested and isolated from resected SFT tumor biospecimens from three individuals with different NAB2-STAT6 fusion subtypes (Table 1). Successful establishment of primary SFT cells was confirmed by continual expression of the SFT-identifying NAB2-STAT6 fusion. As an example, for primary SFT cells isolated at the Moffitt Cancer Center (named Moffitt-ns), total RNA was extracted and subjected to RT-PCR using Lf cells as negative control. As shown in Figure S1A, the 396-bp PCR product from the fusion allele (forward primer P5: within exon 5 of NAB2; reverse primer P6: within exon 19 of STAT6) was only observed in the primary Moffitt-ns cells, which was subsequently validated by Sanger sequencing (Figure S1B) (breakpoint-adjacent region between NAB2 exon 5: 5′-CATTTGCAGG-3′ and STAT6 exon 16: 5′-GCTCTCCACA-3′, highlighted in gray). In addition, the PCR product for the wild-type NAB2 allele (431 bp, forward primer P5: within exon 5 of NAB2; reverse primer P7: within exon 19 of STAT6) was observed in both cell lines (Figure S1A). Taken together, these results confirmed that Moffitt-ns stable cells were heterozygous for both the NAB2exon5-STAT6exon16 fusion and the wild-type NAB2 gene transcripts. Last, similar to Lf-ns, protein analysis by ICC of the primary cells showed prominent nuclear expression of STAT6 (Figure S1C).

**Table 1:** Engineered and primary SFT cell models that were used in this study

ID	Description	Origin/Primary Location	NAB2-STAT6 fusion type	Matching *in vivo* mouse model
Lf-ns	hTERT-immortalized Lf-based SFT cell model	CRISPR-mediated knock-in strategy	NAB2exon6-STAT6exon17	no
Moffitt-ns	primary SFT cell line established at Moffitt Cancer Center	isolated from SFT patient (brain)	NAB2exon5-STAT6exon16	no
INT-SFT	primary SFT cell line established at Oncology Referral Center (Centro di Riferimento Oncologico)	isolated from SFT patient (intra-abdominal pelvic)	NAB2exon6-NAB2intron6-STAT6exon16	no
IEC139	primary SFT cell line established at Advanced Therapies and Biomarkers in Sarcomas (ATBSarc)	isolated from SFT patient (mesogastrium)	NAB2exon6-STAT6exon16	yes

### *In vitro* efficacy testing of STAT6 3′ UTR-targeting ASOs

**Figure 2:**
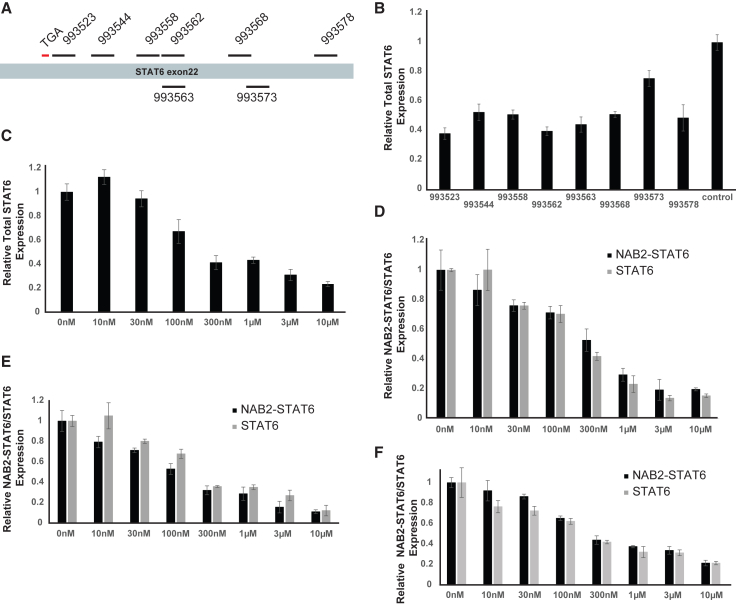
*In vitro* efficacy of unaided delivery of STAT6 3′ UTR-targeting ASOs (A) Schematic illustration of where along exon 22 of STAT6 the 8 STAT6 3′ UTR-targeting ASOs bind. (B) 993523 showed the highest efficacy (62% suppression) against both wild-type (WT) STAT6 and NAB2-STAT6 fusion transcripts in Moffitt-ns cells with gymnotic delivery at concentration of 1 μM after 48 h. (C) In Moffitt-ns cells, 993523 efficiently suppressed both WT STAT6 and NAB2-STAT6 fusion transcripts (IC_50_ of 199.4 nM). 993523 efficiently suppressed both the NAB2-STAT6 fusion and WT STAT6 transcripts in (D) Lf-ns cells (IC_50_ of 299.9 nM and 206.3 nM). (E) INT-SFT cells (IC_50_ of 115.7 nM and 199.5 nM), and (F) IEC139 cells (IC_50_ of 250.8 nM and 201.7 nM).

ASOs targeting the NAB2-STAT6 fusion transcripts were designed within the C-terminal of the human STAT6 transcript, which includes the 3′ untranslated region (UTR) sequence (∼1.1 kb), since all NAB2-STAT6 fusions contain this region. In addition, previous studies have demonstrated that depletion of wild-type STAT6 may elicit beneficial effects in cancer therapeutics via inhibition of M2 macrophage differentiation.^37^^,^^38^^,^^39^^,^^40^ Therefore, we designed a panel of eight STAT6 3′ UTR-targeting ASOs (Figure 2A; Table S2) to suppress the expression levels of both wild-type STAT6 and NAB2-STAT6 fusion transcripts. All STAT6 ASOs are 16 nucleotides in length and connected sequentially by phosphorothioate internucleotide linkages. All ASOs have three 2′−4′ cEt-modified ribonucleotides at both the 5′- and 3′-ends, which confers an increased affinity to the target mRNA and increased resistance to exonucleases and endonucleases within the cell. The central portion is composed of 10 deoxynucleotides, which enables RNase H1 to recognize and cleave the target RNAs in the ASO:RNA duplex.^41^^,^^42^^,^^43^^,^^44^^,^^45^^,^^46^^,^^47^^,^^48^^,^^49^

To evaluate the *in vitro* efficacies of these candidate ASOs against both wild-type STAT6 and NAB2-STAT6 fusion transcripts (for real-time RT-PCR, forward primer P8: within exon 16 of STAT6; reverse primer P9: within exons 17 and 18 of STAT6), Moffitt-ns cells were treated with 1 μM ASOs using gymnotic delivery (i.e., directly added to the growth medium without any transfection agents). As shown in Figure 2B, most ASOs caused a significant reduction of the target transcripts with candidates 993523, 993562, and 993563 showing the highest efficacies (62%, 60%, and 56% decrease, respectively). Next, to determine the potency of the best ASO candidate 993523, we performed dose-response assays using gymnotic delivery of 993523 and primary Moffitt-ns cells. Indeed, 993523 efficiently suppressed the expression of total STAT6 transcripts (primer recognized both wild-type STAT6 and NAB2-STAT6 fusion transcripts combined) in Moffitt-ns cells (Figure 2C) (half-maximal inhibitory concentration [IC_50_] of 199.4 nM).

To evaluate specificity and robustness of STAT6 ASOs, we further subjected the three top ASO candidates (993523, 993562, and 993563) to three additional SFT cell models (Lf-ns, INT-SFT, and IEC139). These cell lines are from different cell type backgrounds as well as NAB2-STAT6 fusion types (Table 1). Specifically, for 993523 in Lf-ns fusion cells, we designed two sets of primers. One primer set recognized only the NAB2-STAT6 fusion transcript (forward primer P10: within exon 6 of NAB2; reverse primer P11: within exon 6 of NAB2 and exon 17 of STAT6), and the other primer only recognized the wild-type STAT6 transcript (forward primer P12: within exon 16 of STAT6; reverse primer P13: within exon 17 of STAT6). As shown in Figure 2D, both transcripts were suppressed by 993523 with comparable potency (IC_50_ of 299.9 nM for the NAB2-STAT6 fusion transcript, and IC_50_ of 206.3 nM for STAT6 transcript). Similar results were also observed in INT-SFT cells (Figure 2E), which showed an IC_50_ of 115.7 nM for the NAB2-STAT6 fusion transcript (forward primer P14: within exon 6 of NAB2; reverse primer P15: within exon 16 of STAT6), and an IC_50_ of 199.5 nM for STAT6 transcript (forward primer P16: within exon 15 of STAT6; reverse primer P17: within exon 16 of STAT6), as well as in the IEC139 cells (Figure 2F) (same primer sets as in INF-SFT cells, IC_50_ of 250.8 nM for the NAB2-STAT6 fusion transcript, and IC_50_ of 201.7 nM for STAT6 transcript). Taken together, our results demonstrated that 993523, and to a lesser extent 993562 and 993563, can efficiently target both wild-type STAT6 and NAB2-STAT6 fusion transcripts in SFT cells (Table 2). Moreover, these studies prove that both STAT6 and NAB2-STAT6 fusion transcripts contain the targeting site for the three candidate ASOs.

**Table 2:** IC_50_ values for STAT6 3′ UTR-targeting ASO inhibition of either NAB2-STAT6 or STAT6 transcript expression in SFT cell models

IC_50_ (nM)	993523	993562	993563
Lf-ns/NAB2-STAT6	299.9	309.4	378.1
Lf-ns/STAT6	206.3	343.1	1,020.5
INT-SFT/NAB2-STAT6	115.7	1472.5	2,574.9
INT-SFT/STAT6	199.5	3451.5	706.7
IEC139/NAB2-STAT6	250.8	966.8	,2452.7
IEC139/STAT6	201.7	1,241.8	1578

**Figure 3:**
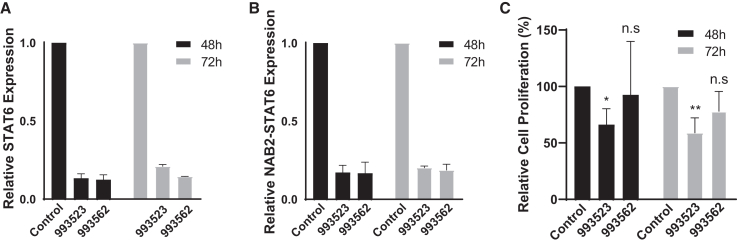
*In vitro* efficiency of liposome-mediated delivery of STAT6 3′ UTR-targeting ASOs RNAiMAX-mediated delivery of 40 nM of ASOs 993523 or 993562 efficiently decreased the expression of (A) STAT6 and (B) NAB2-STAT6 fusion transcripts in IEC139 cells at both 48 and 72 h. (C) RNAiMAX-mediated delivery of ASO 993523 suppressed IEC139 cell proliferation at both 48 and 72 h. For statistical analysis, two-tailed t-tests were conducted. ∗p < 0.05; ∗∗p < 0.01; n.s., no significant difference.

Efficient delivery of ASOs remains a challenge in the clinical setting; accordingly, various vehicles, from DNA nanostructures to exosomes, have been explored.^38^ Herein, using liposome RNAiMAX (Thermo Fisher Scientific), our *in vitro* transfection results showed that 40 nM of ASOs 993523 or 993562 can be efficiently delivered into IEC139 cells and subsequently downregulate the expressions of both wild-type STAT6 and NAB2-STAT6 fusion transcripts (Figures 3A and 3B). Specifically, for STAT6 the addition of 40 nM of 993523 induced an 86.6% and 79.0% decrease after 48 and 72 h, respectively, and 993562 induced an 87.5% and 85.6% decrease after 48 and 72 h, respectively. Similarly, for NAB2-STAT6 the addition of 40 nM 993523 induced an 82.8% and 79.8% decrease after 48 and 72 h, respectively, and 993562 induced an 83.3% and 81.3% decrease after 48 and 72 h, respectively. More important, downregulation of NAB2-STAT6 and STAT6 transcripts by 40 nM of 993523 resulted in a 38.9% and 41.0% decrease in cell proliferation at 48 and 72 h, respectively, compared with the control ASO (Figure 3C). These results highlight the ASOs anti-tumor potential.

### Characterization of the IEC139 patient-derived xenograft mouse model

**Figure 4:**
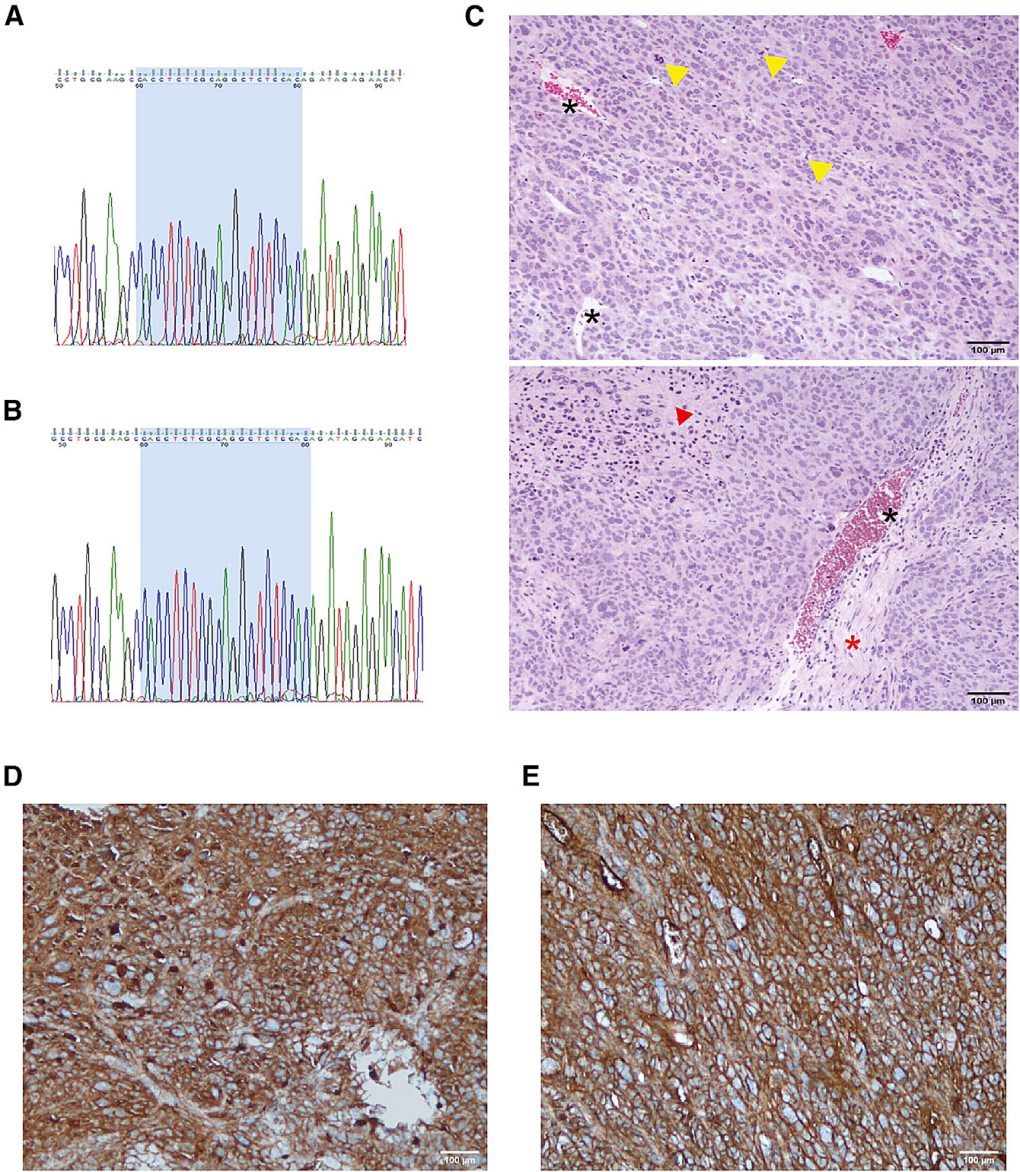
Characterization of the IEC139 primary cells and PDX models The NAB2exon6-STAT6exon16 fusion type was confirmed by harvesting the total RNA from (A) IEC139 primary cells and (B) PDX tumors and subsequent RT-PCR and Sanger sequencing. (C) Hematoxylin and eosin staining of PDX tumor tissues showed hypercellular proliferation of fusiform cells, with enlarged “staghorn” blood vessels (black asterisks) and collagenous components (red asterisks). A grade III SFT was indicated by the high number of mitoses (yellow arrowheads), marked atypia, nuclear pleomorphism, and necrotic areas (red arrowheads). (D) IHC assays showed strong nuclear staining for STAT6 (brown) in the PDX tumor tissues. (E) IHC assays showed strong membrane staining for CD34 (brown) in the PDX tumor tissues.

Tumor tissues used in the IEC139 patient-derived xenograft (PDX) mouse model were originally resected from a patient with SFT (NAB2exon6-STAT6exon16 fusion type) in the mesogastrium (signed consent in accordance with the institutional guidelines of the biobank at the University Hospital Virgen del Rocio). In addition to the development of an IEC139 PDX mouse model (see “materials and methods/generation of IEC139 PDX mouse model”) we also established a matching IEC139 primary SFT cell line (Table 1).^50^ To identify the NAB2-STAT6 fusion type of the IEC139 PDX model, total RNA was extracted from resected PDX tumor samples and subjected to RT-PCR and Sanger sequencing using primers specific to the fusion type (forward primer P18: within exon 6 of NAB2; reverse primer P19: within exon 16 of STAT6). As shown in Figures 4A and 4B, the IEC139 cell line and the corresponding PDX samples were confirmed to have a NAB2exon6-STAT6exon16 fusion type (breakpoint between NAB2 exon 6: 5′-CACCTCTCGCAG-3′ and STAT6 exon 16: 5′-GCTCTCCAC-3′, highlighted in blue). Importantly, histological analysis of the PDX tumor was consistent with the clinical presentation of SFT, showing proliferation of spindle and epithelioid cells with marked atypia and nuclear pleomorphism, arranged randomly on a fibrous stroma with necrotic areas (Figure 4C). Similar to pathological cases of SFT, expanded and branched vessels containing non-atypical endothelial cells were also observed. We note that the high mitotic index (25 mitoses/mm^2^) together with atypia and pleomorphism are indicative of a high-grade SFT (Figures 4C and S2).^51^ Finally, immunohistochemistry (IHC) demonstrated intense nuclear positivity of STAT6 and diffuse expression of CD34 (Figures 4D, 4E, and S2).^52^ To our knowledge, this is the only xenograft animal model available for non-dedifferentiated SFT.

### *In vivo* tolerability testing of STAT6 3′ UTR-targeting ASOs

**Figure 5:**
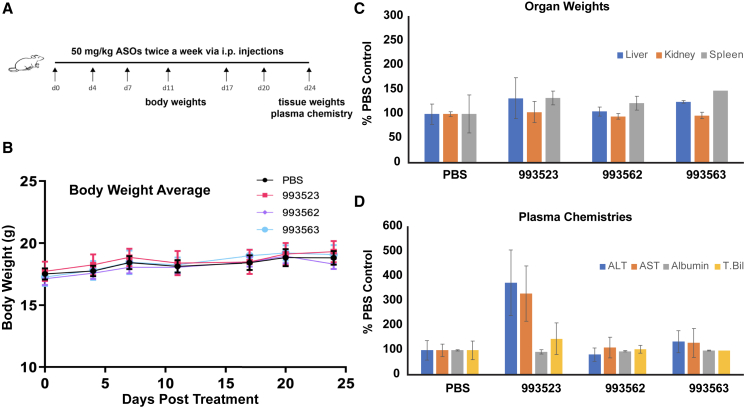
*In vivo* tolerability testing of STAT6 3’-UTR-targeting ASOs (A) Schematic illustrating the injection interval and frequency for the *in vivo* tolerability testing experiments. (B) The animals’ averaged body weight did not significantly change during the treatment course with ASOs 993523, 993562, and 993563 (n = 4). (C) ASOs 993523, 993562, and 993563 did not induce a significant change in the liver, kidney, or spleen of mice (n = 4). (D) ASOs 993562 and 993563 did not induce significant hepatotoxicity in mice (n = 4). ASO 993523 induced a moderate but not clinically precluding increase in serum ALT and AST levels (p < 0.01 using ANOVA with Dunnett’s post hoc tests).

Some high-affinity ASOs have been reported to induce hepatoxicity and immune system activation *in vivo*, potentially due to their immunostimulatory nature or hybridization-mediated off-target effects.^53^^,^^54^^,^^55^ To probe the *in vivo* tolerability of our three candidate STAT6 3′ UTR-targeting ASOs (993523, 993562, and 993563), four female C57BL/6 mice per ASO group were treated with intraperitoneal injections of 50 mg/kg ASO twice a week for up to 24 days (Figure 5A). Next, the animals were evaluated using three metrics: body weight, tissue weight, and level of serum biomarkers. As shown in Figure 5B, all three candidate SFT-ASOs did not affect body weights, compared with the PBS control. Similarly, organ weights (liver, kidney, and spleen) were not significantly affected (Figure 5C). Finally, to evaluate ASO-induced hepatotoxicity, serum levels of biomarkers alanine transaminase (ALT), aspartate transaminase (AST), albumin, and total bilirubin were measured.^56^ As shown in Figure 5D, no significant increases were observed in 993562- and 993563-treated groups for all four biomarkers, and only a moderate but not clinically precluding increase in serum ALT and AST levels (∼3- to 4-fold compared with PBS control) was observed in the 993523-treated group. Taken together, these results indicated that the selected SFT-ASOs 993523, 993562, and 993563 displayed acceptable *in vivo* tolerability profiles.

### *In vivo* efficacy testing of the STAT6 3′ UTR-targeting ASOs

**Figure 6:**
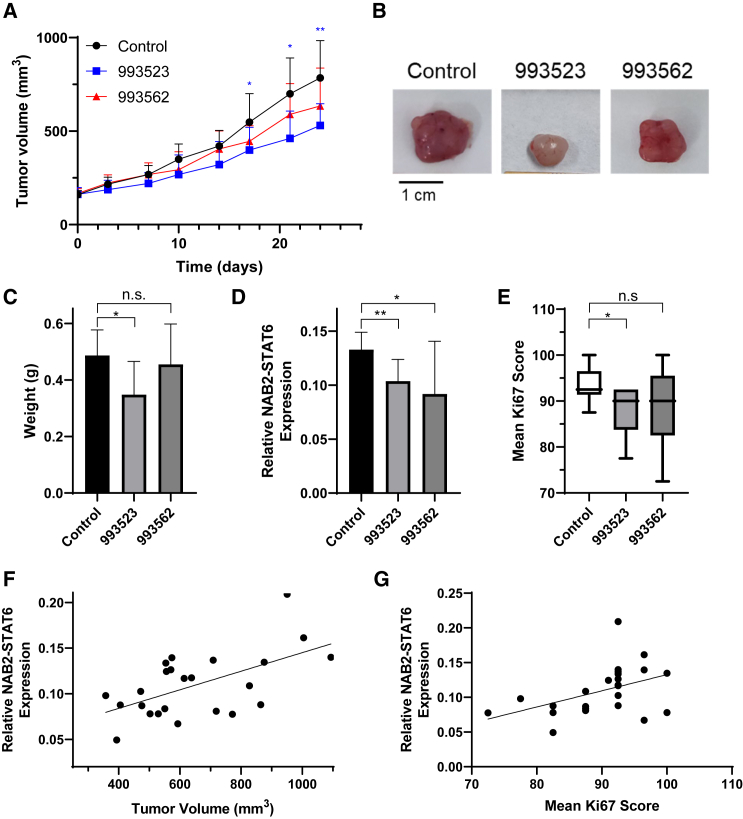
*In vivo* efficacy testing of STAT6 3′ UTR-targeting ASOs in IEC139 PDX mouse models (A) Tumor volumes were significantly lower for ASO 993523-treated mice between days 17–24, compared with the untreated control tumors. No significant differences were observed for ASO 993562-treated group (n = 8). (B) Representative tumor samples for each treatment group on day 25. (C) The ASO 993523-treated group showed a significant decrease in tumor weight by day 25 (n = 8). (D) Both ASOs 993523 and 993562 demonstrated significant downregulation of the expression of NAB2-STAT6 fusion transcripts within the tumor tissues by day 25 (n = 8). (E) The ASO 993523-treated group showed a significantly lower mean Ki67 score compared with the control group. (F) Expression levels of NAB2-STAT6 fusion transcripts exhibited a positive correlation with tumor volumes (Pearson correlation coefficient: 0.58; p = 0.003). (G) Expression levels of NAB2-STAT6 fusion transcripts exhibited a positive correlation with Ki67 scores (Pearson correlation coefficient: 0.44; p value: 0.030). For statistical analysis, two-tailed t -tests were conducted. ∗p < 0.05; ∗∗p < 0.01; n.s., no significant difference.

To evaluate *in vivo* efficacies of candidate ASOs, 993523 and 993562, 8- to 10-week-old female Foxn1^nu^ athymic nude mice bearing IEC139 PDX were randomized to receive either a treatment ASO or control ASO (792169, which has no targets within the human transcriptome but the same DNA backbone modifications) when the tumor volume reached 150–200 mm^3^. Efficient delivery of ASOs (control and STAT6) to the tumor tissue was confirmed by IHC staining using anti-ASO primary antibody^57^ (Ionis) (Figure S2-ASO panel). Next, as shown in Figure 6A, by day 17, mice treated with 993523 demonstrated a significant decrease in tumor volume compared with the control group. By completion of the study (day 24), the tumor volume for the 993523-treated (530.2 ± 115.9 mm^3^) was on average 32.4% smaller than the control-treated tumors (784.7 ± 199.4 mm^3^) (Figure 6B). Similarly, tumor weights measured at the end of treatment (day 24) were significantly lower in the 993523 group (348 ± 117 mg) compared with the control group (487 ± 90 mg) (Figure 6C). In contrast, treatment with ASO 993562 did not have any statistically significant effects on either tumor volumes or weights (Figures 6A and 6C).

Moreover, ASO 993523-treated tumor tissues had a significant reduction (21.9%) in RNA expression of the NAB2-STAT6 fusion transcript compared with control ASO treated tumors (Figure 6D). In contrast, wild-type STAT6 was not significantly downregulated (Figure S3). In addition, evaluation of IHC Ki67 staining revealed significant less proliferation in the 993523-treated tumors (88.13 ± 5.63) as compared with the control group (93.63 ± 3.88) (Figures 6E and S2/Ki-67 panel). These results are corroborative with the smaller tumor sizes (Figure 6B) and anti-proliferation effects of ASO 993523 observed in our *in vitro* assays (Figure 3C). We note that treatment of ASO 993562 also induced smaller, albeit statistically insignificant, Ki67 score (88.31 ± 9.92; p = 0.1447), as well as lower expression of the NAB2-STAT6 fusion transcript (decrease of 30.9%).

We want to emphasize that our data demonstrated a clear positive correlation between relative NAB2-STAT6 fusion transcript expression level and either tumor volume (Figure 6F) (Pearson correlation coefficient, 0.58) or Ki67 score (Figure 6G) (Pearson correlation coefficient, 0.44). These results, together with observations that no significant differences in STAT6 expression were found between the treatment groups (Figure S3), further support an oncogenic role of the NAB2-STAT6 fusion transcript in SFT.

Finally, consistent with our earlier *in vivo* tolerability testing results (Figure 5), no statistically significant body weight fluctuations were observed throughout the treatment period for any of the STAT6 3′ UTR-targeting ASOs (Figure S4). Taken together, our results demonstrate that 993523 can safely exert anti-tumor effects in SFT PDX models.

## Discussion

In this study, we have shown that STAT6 3′ UTR-targeting ASO 993523 effectively decreased the expression of NAB2-STAT6 fusion transcripts both *in vitro* and *in vivo* and, importantly, exerted anti-tumor impact in the SFT PDX model. These effects were likely attributed to suppressed cell proliferation, as evidenced by both inhibition of cell proliferation in the 993523-transfected SFT cells (Figure 3) and reduction of Ki67-positive cells in the 993523-treated SFT PDX model (Figure 6). It should be noted that, although NAB2-STAT6 fusion accounts for almost all known SFT cases, recently we have reported one rare SFT case in which a different fusion type (NFIX-STAT6) was observed.^58^ Nevertheless, our current ASO design strategy, which targets the 3′ UTR of STAT6, should also treat those SFT fusions.

Broadly speaking, clinical adoption of ASOs poses challenges, primarily due to concerns surrounding their safety attributes and their capacity to effectively combat tumors. Regarding safety, herein we show STAT6 ASOs have a satisfactory safety profile in mice PDX models. Furthermore, recent studies using other STAT6-targeting ASOs have similarly reported the absence of toxicity in their mouse models, whether delivered with exosomes or alone.^38^^,^^59^ That said, it is important to highlight that our candidate ASO 993523 exhibits the ability to target both NAB2-STAT6 fusion and the conventional wild-type STAT6 transcripts (as illustrated in Figure 2) and the consequences of inhibiting the wild-type STAT6 transcripts are yet to be understood fully. To address this concern, for each variant of the NAB2-STAT6 fusion, an alternative approach could involve the design of ASOs specifically aimed at the fusion junctions. For example, in our recent publication,^30^ we developed an ASO tailored to target specifically the NAB2exon6-STAT6exon17 fusion junction that, when delivered using RNAiMAX, achieved a 58% suppression of expression of NAB2-STAT6 transcript at 1 μM within 48 h. Nevertheless, it should be noted that STAT6-targeting ASO 993523 showed even greater targeting efficiency, particularly when a gymnotic delivery method was adopted. Taken together, these results shed light on the delicate balance between two competing aspects: (a) specificity for the fusion transcript to prevent potential off-target effects and (b) efficacy to ensure a therapeutic benefit.

Another clinical challenge relates to the ability of ASOs to counteract tumor growth. Although intratumor suppression of the NAB2-STAT6 fusion transcript was relatively modest (as evidenced by the 21.9% decrease seen with 993523, as shown in Figure 6C), tumor growth was still significantly slowed (Figures 6A and 6B). This observation suggests that the NAB2-STAT6 fusion protein plays a critical role in the expansion of SFT and, thus, could serve as an effective therapeutic target for SFTs. It is conceivable that a more pronounced decrease in the fusion transcript level, and subsequently stronger anti-tumor effects and extended survival, could be achieved by extending the treatment duration. This assumption is supported by the positive correlation between tumor volume and NAB2-STAT6 expression levels (Figure 6F), the gradual potency in tumor suppression in 993523-treated mice from day 17 onwards, as well as previous reports that showed tumor growth curves of anti-STAT6 ASO-treated mice diverging from the control group over an extended duration (≤35 days).^38^^,^^59^ Furthermore, the administration of higher dosages of ASOs is a viable option, given the low *in vivo* toxicity observed in our experiments. Subsequent investigations centered on enhancing the delivery of ASOs to the intended target and cellular uptake are needed and underway.^60^

An alternative approach to increase STAT6 ASO efficacy in treating SFTs includes the utilization of combinational therapies. ASOs have been shown to exert synergistic anti-tumor effects when used in combination with small-molecule chemotherapeutic agents. For example, patients with refractory or relapsed acute myeloid leukemia exhibited improved clinical outcomes when anti-survivin ASO (LY2181308) was administrated in conjunction with cytarabine and idarubicin.^61^ Our data (Figure S5) showed that the tyrosine kinase inhibitor (TKI) sitravatinib substantially suppressed the *in vitro* proliferation of IEC139 (IC_50_, 1.1 nM). Sitravatinib is a multi-kinase inhibitor that has strong anti-angiogenic activity, while also inhibiting the function of TAMs (Tyro3, AXL, and MerTK) and DDR1.^62^ Notably, sitravatinib is currently undergoing clinical evaluation for multiple indications including sarcomas.^63^ Collectively, these studies suggest a combined treatment strategy involving ASO 993523 and sitravatinib or other TKIs (such as pazopanib^64^^,^^65^) could enhance the overall treatment effectiveness.

In conclusion, while the inhibitory effects related to the expression of NAB2-STAT6 fusion transcripts in 993523-treated SFT PDX model may be relatively mild, it is noteworthy that even this modest inhibition has led to a significant decrease in tumor size, highlighting the remarkable sensitivity of SFT growth to NAB2-STAT6 inhibition. Future studies investigating the mechanistic details of NAB2-STAT6 in SFT (e.g., RNA sequencing assays using ASO 993523 as perturbing agents) and validating the observed positive correlation between NAB2-STAT6 expression (at both mRNA and protein levels) and cell proliferation in a clinical cohort will be vital for advancing the field and ultimately improving outcomes for SFT patients.

## Materials and methods

### Isolation of primary Moffitt-ns cells

Primary SFT tissues were isolated at Moffitt Cancer Center under the Total Cancer Care research protocol and approved institutional review board, Institutional Biosafety and Chemical Safety Committee (IBCC), and Material Transfer Agreements (MTA) protocols. Tissues were cut into small pieces and incubated in RPMI 1640 (Gibco; catalog number: 11879020) media containing collagenase type II (1 mg/mL, Thermo Fisher Scientific; catalog number: NC9460908) and DNase I (30 U/mL, Roche; catalog number: 11284932001) for 40 min at 37°C. Next, the digested mixture was centrifuged at 1,000 rpm for 10 min at 4°C and the pellets were subjected to another round of collagenase type II/DNase I treatment. Finally, the pellet was resuspended in 10 mL RPMI-1640 media containing 10% fetal bovine serum (FBS; Corning; catalog number: 35-011-CV), 0.1 mM minimal essential medium nonessential amino acids (Invitrogen; catalog number: 11140-050), and penicillin (0.045 U/mL) and streptomycin (0.045 U/mL) (penicillin-streptomycin liquid; Invitrogen; catalog number: 15140), and subsequently maintained at 37°C with 5% CO_2_.

### Mammalian cell culture

The hTERT-immortalized Lf cells were acquired from the American Type Culture Collection (ATCC; catalog number: CRL-4058) and maintained at 37 °C, 100% humidity, and 5% CO_2_. The cells were grown in Fibroblast Basal Medium (ATCC; catalog number: PCS-201-030) supplemented with Fibroblast Growth Kit-Low Serum (ATCC; catalog number: PCS-201-041) and 0.3 μg/mL puromycin (Gibco; catalog number: A1113803). To pass the cells, the adherent culture was first washed with PBS (Dulbecco’s PBS; Mediatech; catalog number: 21-030-CM), then trypsinized with Trypsin-EDTA for Primary Cells (ATCC; catalog number: PCS-999-003), and finally diluted in fresh medium. Same protocol was used for maintaining Lf-ns cells, except that hygromycin (47.5 μg/mL; Thermo Fisher Scientific; catalog number: 10687010) was included in the complete growth medium.

The INT-SFT cell line was established using SV40 Large T Antigen-mediated immortalization by Dr. Roberta Maestro’s group at Oncology Referral Center (Centro di Riferimento Oncologico). The IEC139 primary cell line was established by Dr. Javier Martin-Broto’s group at Advanced Therapies and Biomarkers in Sarcomas (ATBSarc). INT-SFT and IEC139 cells were maintained in RPMI-1640 media (Thermo Fisher Scientific; catalog number: 11-875-085) containing 10% FBS (Invitrogen; catalog number: 26140), MEM non-essential amino acids (Invitrogen; catalog number: 11140–050) and 0.045 U/mL penicillin and 0.045 U/mL streptomycin (penicillin-streptomycin liquid; Invitrogen; catalog number: 15140). To pass the cells, the adherent culture was first washed with PBS (Dulbecco’s PBS; Mediatech; catalog number: 21-030-CM), then trypsinized with Trypsin-EDTA (0.25% Trypsin with EDTAX4Na; Invitrogen; catalog number: 25200) and finally diluted in fresh medium.

### Long-range genomic PCR and RT-PCR

For long-range genomic PCR, total genomic DNAs were harvested using DNeasy Blood & Tissue kit (Qiagen; catalog number: 69504), and long-range PCR reactions were performed using Q5 High-Fidelity 2× Master Mix (New England Biolabs; catalog number: M0492). We used 100 ng genomic DNAs as the template, and the PCR conditions were first 1 cycle of 98 °C for 30 s, followed by 40 cycles of 98 °C for 10 s, 66 °C for 30 s, and 72 °C for 2 min. The PCR products were subjected to 1% agarose gel electrophoresis and the DNA bands of interest were purified using QIAquick Gel Extraction Kit (Qiagen; catalog number: 28706) and subjected to direct Sanger sequencing (Genewiz) and analyzed using FinchTV (Geospiza).

For RT-PCR, total RNAs were harvested using RNeasy Mini kit (Qiagen; catalog number: 74106) and cDNAs were made using QuantiTect Reverse Transcription kit (500 ng RNA; Qiagen; catalog number: 205311). The cDNAs were then subjected to PCR reactions using Q5 High-Fidelity 2× Master Mix and the PCR conditions were: first 1 cycle of 98 °C for 30 s, followed by 40 cycles of 98 °C for 10 s, 63 °C for 30 s, and 72 °C for 1 min. The PCR products were subjected to 1% agarose gel electrophoresis and the DNA bands of interest were purified using QIAquick Gel Extraction Kit and subjected to direct Sanger sequencing (Genewiz) and analyzed using FinchTV (Geospiza).

### ICC

We seeded 20,000 cells in 24-well glass plates and 16 h later, the cells were washed with 500 μL ice-cold PBS, and then treated with pre-chilled 100% methanol (Thermo Fisher Scientific; catalog number: A454) at 25 °C for 5 min. The cells were then washed three times with ice-cold PBS, and subsequently incubated with 1% BSA (Thermo Fisher Scientific; catalog number: 37525) in 1× PBS with 0.1% Triton X-100 (Sigma-Aldrich; catalog number: 93443) at 25 °C for 1 h. The cells were then incubated with Alexa Fluor 594 Anti-STAT6 antibody (1:100; Abcam; catalog number: ab207012) in 1% BSA in 1× PBST at 4 °C overnight. Next, the cells were washed with ice-cold PBS for three times, followed with incubation with DAPI (1 μg/mL in PBS; Thermo Fisher Scientific; catalog number: 62248) at 25 °C for 5 min. The cells were then washed with ice-cold PBS three times. Finally, cells were imaged using the Olympus IX81 microscopy and a Precision Control environmental chamber. The images were captured using a Hamamatsu ORCA-03 Cooled monochrome digital camera. The filter sets (Chroma) are as follows: ET390/18× (excitation) and ET460/60m (emission) for BFP, and ET560/40× (excitation) and ET630/75m (emission) for mKate. Data collection and processing was performed in the software package Slidebook 5.0.

### *In vitro* ASO treatment

All ASOs were dissolved in PBS. ASO treatments were performed either without transfection reagents (gymnotic delivery) or using Lipofectamine RNAiMAX (Invitrogen; catalog number: 13778075). For gymnotic delivery, cells were seeded in complete growth medium and the following day, ASOs were added to the growth medium at desired final concentrations. For RNAiMAX-mediated transient transfection, cells were seeded in complete growth medium in six-well plates (Sigma-Aldrich; catalog number: CLS3516) and the following day, 100 pmol ASOs were added according to the manufacturer’s protocol. After 48 or 72 h, the cells were harvested for further analysis.

### Real-time RT-PCR

For real-time RT-PCR assays, total RNAs were extracted using RNeasy Mini Kit. First-strand cDNAs were synthesized using QuantiTect Reverse Transcription kit. Next, quantitative PCR was performed using the KAPA SYBR FAST universal qPCR Kit (Kapa Biosystems; catalog number: KK4601), with GAPDH as the internal control. The forward primer (P20) for human GAPDH was 5′-AATCCCATCACCATCTTCCA-3′ and the reverse primer (P21) for human GAPDH was 5′-TGGACTCCACGACGTACTCA-3′. Quantitative analysis was performed using the 2^−ΔΔCt^ method. Fold-change values were reported as means with SDs.

### Cell proliferation assay

IEC139 cells were seeded in complete growth medium and the following day ASOs were transiently transfected using RNAiMAX according to the manufacturer’s protocol. After 48 and 72 h, cells were trypsinized with 0.25% trypsin-EDTA at 37 °C for 5 min. Trypsin-EDTA was then neutralized by adding the complete medium. The cell suspension was then counted using a hemacytometer (Paul Marienfeld GmbH & Co. KG; catalog number: 0640011). All experiments were performed in triplicate.

### Generation of the IEC139 PDX mouse model

The tumor tissues for preparing IEC139 PDX mouse model were originally resected from an SFT patient (NAB2exon6-STAT6exon16 fusion type) with signed consent for the use in pre-clinical research. Briefly, under anesthesia and sterile conditions, a small incision on the skin was introduced in the flank area of a Foxn1^nu^ athymic nude mouse (Jackson Lab; strain number: 002019). Next, the harvested tumor fragment was placed in the cavity underneath the skin, and then the wound was stitched. This instance was considered as passage 0 of the SFT PDX mouse model. The tumor was resected when it reached the volume of 1,500 mm^*3*^, and subsequently reimplanted in mice following the same protocol until passage 3. At this point, the model was considered as being stable, which guarantees growth upon reimplantation. For long-term storage, the harvested PDX tumors were frozen in the RPMI-1640 media containing 10% DMSO (Sigma-Aldrich; catalog number: D2650) and the tissues were found to be viable after re-thawing.

### Hematoxylin and eosin staining

The paraffin-embedded tissue slides (10 μm) were first deparaffinized by heating at 60 °C for 10 min. The slides were then re-hydrated and staining using hematoxylin 560 (Leica; catalog number: 3801570). The slides were then counterstained using Eosin Y 515 (Leica; catalog number: 3801615). After dehydration, one drop of mounting medium (Abcam; catalog number: ab64230) and a glass cover were added to each slide, and slides were then observed using a Leica DMi1 Microscopy.

### IHC

Paraffin-embedded tissue slides (10 μm) were deparaffinized by immersing in Xylene for 10 min, followed by sequential rehydration in anhydrous ethanol for 5 min and 96% ethanol for 5 min, and finally, in water. For automated staining, an advanced next generation stainer, the Benchmark ULTRA (Roche Diagnostics), was used with an integrated workflow. Before staining, the tissue samples underwent pretreatment using the ULTRA Cell Conditioning Solution (ULTRA CC1) buffer (Roche Diagnostics). CD34 staining required 56 min of pretreatment, while STAT6 staining required 64 min. The primary antibodies used in the staining process were prediluted and ready to use. CD34 staining was achieved using the QBEND/10 antibody (Roche Diagnostics) and required 8 min of incubation, while STAT6 staining used the EP325 antibody (Cell Marque; 1:50 dilution) with an incubation time of 32 min. Visualization of the stained samples was carried out using the OptiView DAB IHC Detection Kit (Roche Diagnostics) and Dako Autostainer Link 48 (Agilent), following the manufacturers’ instructions. Ki67 staining (anti-Ki67 rabbit monoclonal antibody, Roche Diagnostics) in PDX paraffin tissue microarray was evaluated by examining multiple sections along the tumor. The percentages of Ki67-positive cells were recorded for each section, and the mean values across multiple sections were then calculated as the Ki67 final score.

### Measurement of serum levels of AST, ALT, blood urea nitrogen, total bilirubin, and albumin

All four animals per group on terminal sacrifice were used for clinical chemistry assessment. Blood samples were collected from all surviving animals and put into tubes containing potassium salt of EDTA. The tubes were centrifuged (approximately 10,000 rpm, 10 min, at 4 °C) to obtain plasma. Plasma samples were processed on Olympus AU480 analyzer to obtain clinical chemistry parameters, which included AST, ALT, blood urea nitrogen, total bilirubin, and albumin.

### *In vivo* ASO treatment

A total of 25 IEC139 PDX animals were used. Foxn1^nu^ athymic nude mice were purchased from Charles River Laboratories (catalog number: 490CRATHHO) and maintained in a pathogen-free facility, in accordance with Protocol PROEX 163.7/23 approved by the Department of Environment, Agriculture, and Interior Affairs of the Community of Madrid. Briefly, 8- to 10-week-old female Foxn1^nu^ athymic nude mice bearing IEC139 PDX were randomized to receive the treatment ASOs (ASOs 993523 and 993562) or control ASOs (792169, no targets within human transcriptome) when the tumor volume reached 150–200 mm^3^. All ASOs were administered via subcutaneous injection under the neck area (50 mg/kg per injection, 2 injections per week) and the treatment lasted for a total of 24 days. During the treatment period, the animals’ body weight and tumor volume were measured at the time of injection. The tumor size was measured using a digital caliper and the tumor volume was calculated as (*L* × *W* × *W*)/2, where *L* was tumor length and *W* the tumor width. On day 25 (1 day after the last injection), the animals were euthanized. Subsequently, tumor tissues and internal organs were weighted and then collected for further analysis.

### CellTiter-Glo Luminescent Cell Viability assay

Sitravatinib was a gift from Dr. Rolf Brekken at University of Texas Southwestern. The CellTiter-Glo Luminescent Cell Viability kit was purchased from Promega (catalog number: G7573). The cell viability assays were performed according to the manufacturer’s recommendations. Briefly, 3,000 IEC139 cells were seeded on 96-well plate. After 16 h, the cells were treated with sitravatinib at different final concentrations (0, 0.1, 0.3, 1, 3, 10, 30, and 100 nM) for 72 h. Next, the CellTiter-Glo reagent was added to each well and the plate was incubated for 10 min at room temperature, and subsequently the luminescence was measured using an FLUOstar Omega microplate reader (BMG Labtech; catalog number: 0415-0003). All experiments were performed in triplicate.

## Data and code availability

Data collected for this article are available in the supplemental information.

## Supplemental information

Document S1. Figures S1–S5 and Tables S1–S6

Document S2. Article plus supplemental information
