# Supplementary material for: STAT6-targeting antisense oligonucleotides against solitary fibrous tumor
Source: Mol Ther Nucleic Acids. 2024 Feb 15;35(2):102154. doi: 10.1016/j.omtn.2024.102154 (PMC10950871; doi:10.1016/j.omtn.2024.102154)
Supplement: Document S1. Figures S1–S5 and Tables S1–S6 [file mmc1.pdf]

OMTN, Volume *35*

Supplemental information

STAT6-targeting antisense oligonucleotides against solitary fibrous tumor

Yi Li, Jose L. Mondaza-Hernandez, David S. Moura, Alexey S. Revenko, Angelica Tolentino, John T. Nguyen, Nam Tran, Clark A. Meyer, Jose Merino-Garcia, Rafael Ramos, Davide Di Lernia, Javier Martin-Broto, Heather N. Hayenga, and Leonidas Bleris

Supplemental materials

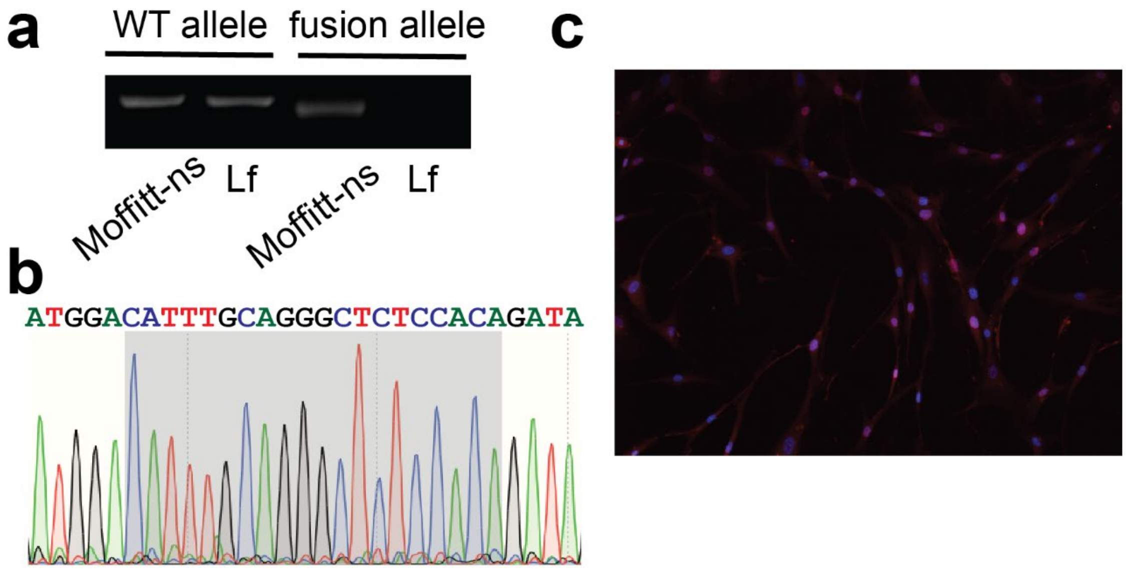

Figure S1. Primary SFT cell line established at Moffitt Cancer Center (Moffitt-ns). (a) RNA RT-PCR assays confirmed that Moffitt-ns cells are heterozygous. **(b)** Sanger confirmed the fusion as NAB2exon5-STAT6exon16. **(c)** ICC showed prominent nuclear expression of the fusion protein. (red) anti-STAT6 antibody conjugated with Alexa Fluor 594, (blue) DAPI.

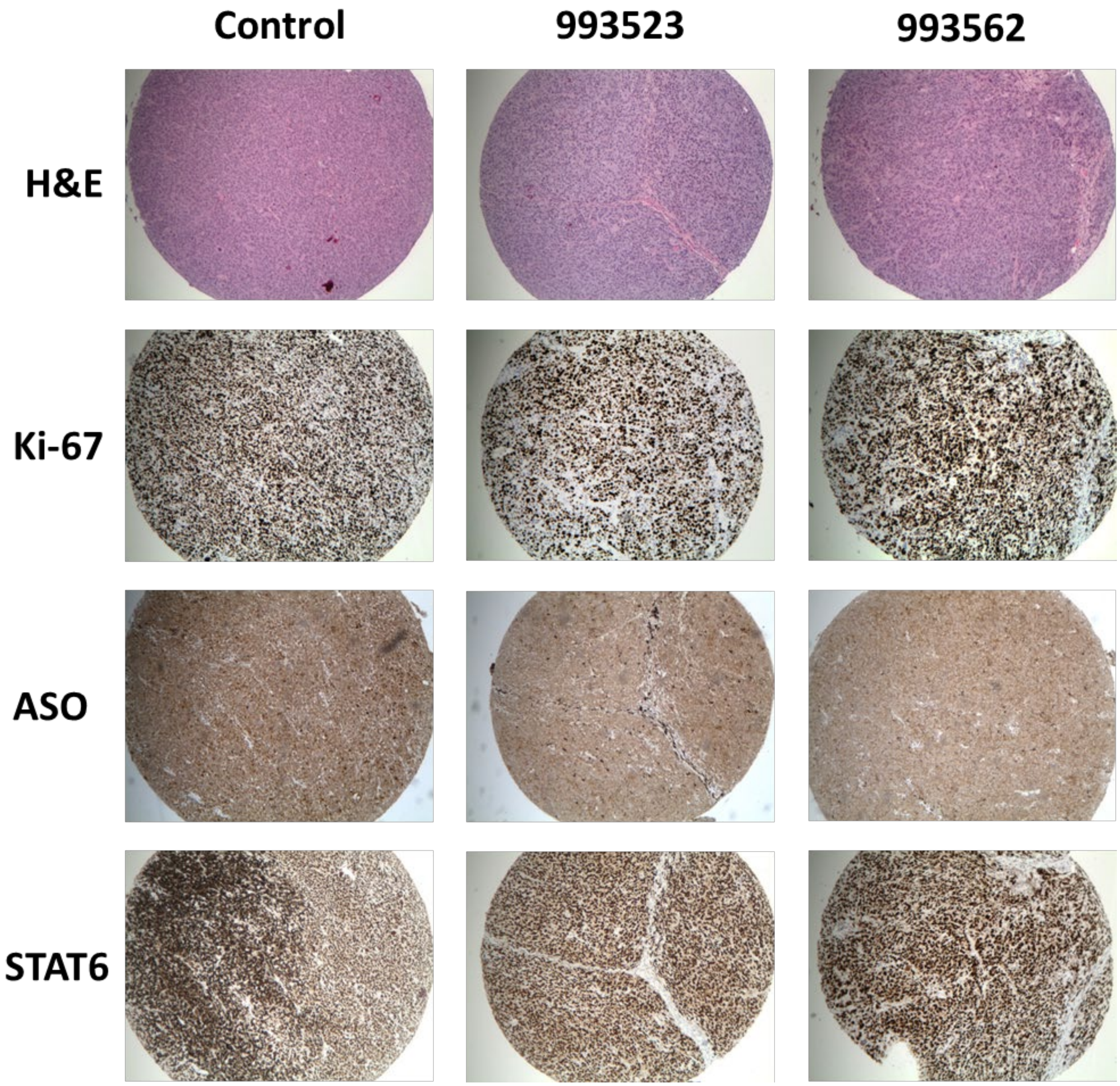

Figure S2. Representative images for H&E and IHC (immunohistochemistry) staining using IEC139 PDX tissues. The percentage of Ki-67-positive cells were significantly lower in 993523-treated tumor tissues compared to control-treated sample. In contrast, no significant differences were observed between different treatment groups for ASO or total STAT6 (NAB2-STAT6 fusion and wild type STAT6).

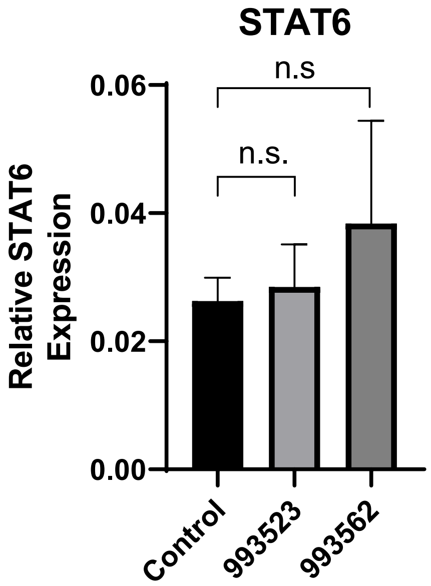

Figure S3. ASOs 993523 and 993562 did not significantly downregulate the expression of wild type STAT6 transcript within the tumor tissues at the end of treatment. For statistical analysis, two-tailed t-tests were conducted.

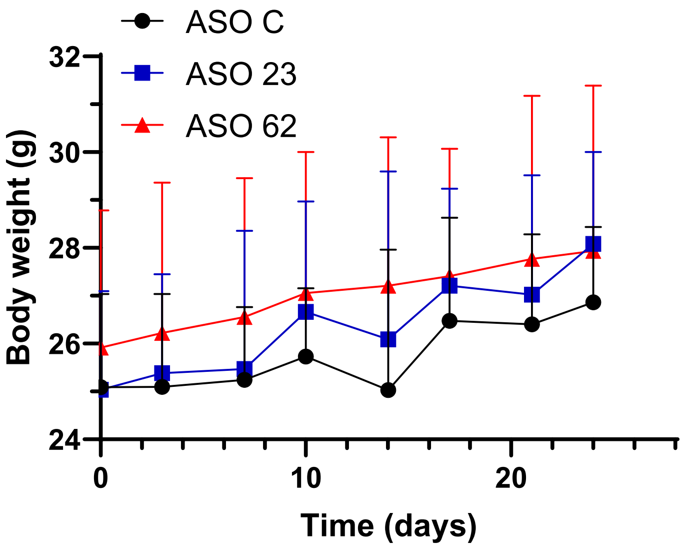

Figure S4. No significant changes in body weight were observed during the entire treatment period among the three treatment groups (n=8).

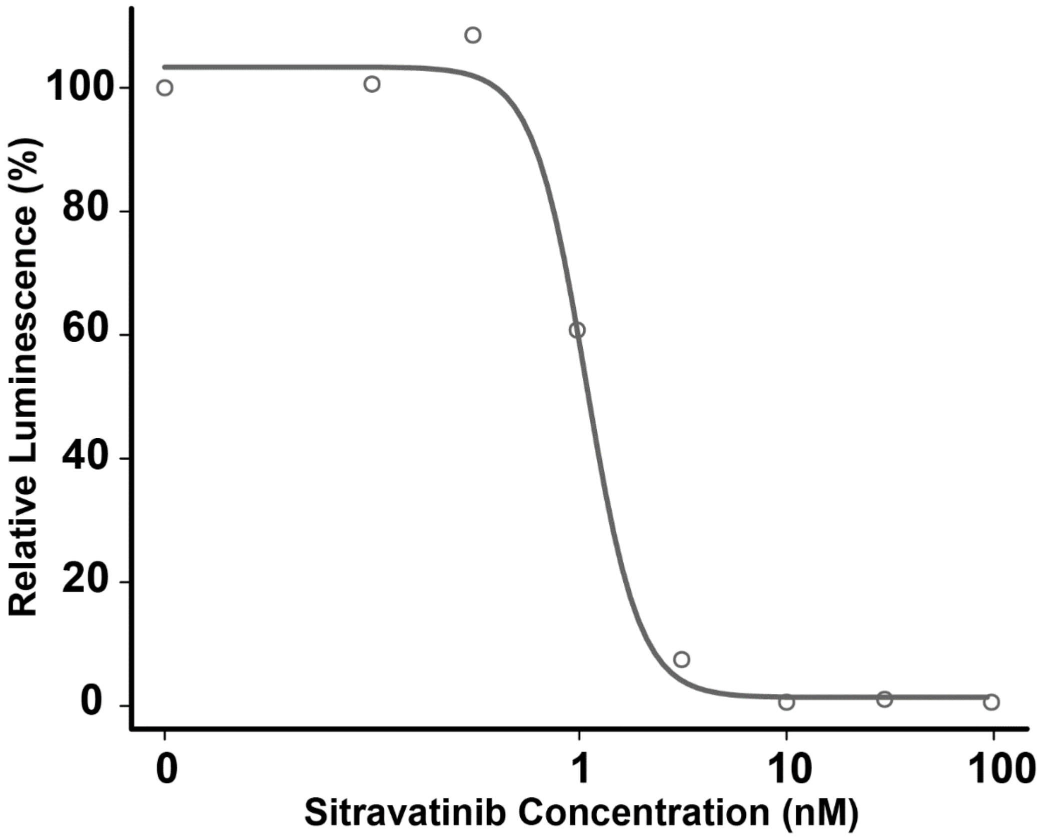

Figure S5. Tyrosine kinase inhibitor Sitravatinib potently inhibits SFT cell proliferation *in vitro* (IC50: 1.1 nM).

Table S1. Primers used in this study.

Primer	Sequence (5'->3')
P1	GGTCATGTCCAAGGCTGACGCCGCCCCCTG
P2	GTAGCTGGGACATAACCCCTGCCATCCTTACC
P3	GGTCATGTCCAAGGCTGACGCCGCCCCCTG
P4	CCTCCCCTCCCTGGCTGTGCGTAGCTCTGT
P5	CCTGTCTGGGGAGAGTCTGGATG
P6	GGGGGGATGGAGTGAGAGTGTG
P7	GGGGGGATGGAGTGAGAGTGTG
P8	CTTTCCGGAGCCACTACAAG
P9	AGGAAGTGGTTGGTCCCTTT
P10	CTGCAGCAGACACTGATGGA
P11	TACCCATCTGTTCAGCTGCG
P12	GAATCCGGGATCTTGCTCAG
P13	CCTGCCATCCTTACCCATCT
P14	CGAAGCCACCTCTCGCAG
P15	CTTGTAGTGGCTCCGGAAAG
P16	AACCTTTCTCCTCCGCTTCA
P17	TGGCTGGATGTTCTCTATCTGT
P18	AGCAGACACTGATGGACGAG
P19	TGGGCTTCTTGGGATAGAGA
P20	AATCCCATCACCATCTTCCA
P21	TGGACTCCACGACGTACTCA

Table S2. Candidate STAT6 3’-UTR-targeting ASOs used in this study.

ID	Sequence (5'->3')	Length	Molecular Weight	Extinction Coefficient
993523	GTAGAAGAGCTGTCTC	16	5456.5166	161.18
993544	CCAATATCCTCTATCC	16	5327.5137	146.08
993558	ACATATCCTAGGTACA	16	5398.2	166.38
993562	CAACTAAGGTGCCAGC	16	5438.5479	157.62
993563	GCAACTAAGGTGCCAG	16	5464.5449	162.18
993568	TTCTATGTGGTCATGC	16	5413.4771	149.32
993573	CAGATAGACACATGTT	16	5424.5176	166.02
993578	AGCATATGTCAGAGAG	16	5475.5278	173.8

Table S3. Lf-ns NAB2exon6-STAT6exon17 mRNA transcript sequence.

AGACAGAGGCGCGGAGGCTCGGAGAGAGAAGACGTGGAGGGAGGGACAGAGCCTG
GACAGCGGTGGACACGGCATCGTGCGCGGGGAAGAGGGCAGCACGCAGCAGGCGC
CGAGCGCCGGGCACCGAGAAGGGCAGCCCGGGTGATCTCCGGCCGTCCATGCACAG
AGCGCCTTCCCCCACAGCCGAGCAGCCGCCGGGCGGAGGGGACAGCGCCCGCCGGA
CCCTGCAGCCCAGACTCAAGCCCAGTGCCCGAGCCATGGCACTGCCTCGGACGCTG
GGGGAGCTGCAGCTGTACCGGGTCCTGCAGCGCGCCAACCTCCTTTCCTACTATGAG
ACCTTCATCCAGCAGGGAGGGGACGACGTGCAGCAGCTGTGTGAGGCGGGTGAGGA
GGAGTTTCTGGAGATCATGGCACTTGTGGGCATGGCCACCAAGCCCCTCCATGTCCG
GCGCCTGCAGAAGGCACTGAGAGAGTGGGCCACCAATCCAGGGCTCTTCAGTCAAC
CAGTGCCTGCTGTTCCCGTCTCCAGCATCCCGCTCTTCAAGATCTCTGAGACTGCGG
GTACCCGGAAAGGGAGCATGAGCAATGGGCATGGCAGCCCAGGGGAAAAGGCAGG
CAGTGCCCGCAGTTTTAGCCCCAAGAGCCCCCTTGAACTTGGAGAGAAGCTATCACC
ACTGCCTGGGGGACCTGGGGCAGGGGACCCCCGGATCTGGCCAGGCCGGAGCACTC
CAGAGTCGGACGTTGGGGCAGGAGGAGAAGAGGAGGCTGGCTCGCCCCCCTTCTCC
CCCCCTGCAGGGGGAGGAGTCCCTGAGGGGACTGGGGCTGGGGGGCTGGCAGCAG
GTGGGACTGGGGGTGGTCCAGACCGACTGGAGCCAGAGATGGTACGCATGGTGGTG
GAAAGTGTGGAGAGGATCTTCCGGAGCTTCCCAAGGGGGGATGCTGGGGAGGTCAC
ATCCCTGCTAAAGCTGAATAAGAAGCTGGCACGGAGCGTTGGGCACATCTTTGAGA
TGGATGATAATGACAGCCAGAAGGAAGAGGAGATCCGCAAATACAGCATCATCTAT
GGCCGTTTCGACTCTAAGCGGCGGGAGGGCAAGCAGCTCAGCCTGCACGAGCTCAC
CATCAACGAGGCTGCTGCCCAGTTCTGCATGAGGGACAACACGCTCTTATTACGGAG
AGTGGAGCTCTTCTCTTTGTCCCGCCAAGTAGCCCGAGAGAGCACCTACTTGTCCTC
CTTGAAGGGCTCCAGGCTTCACCCTGAAGAACTGGGAGGCCCTCCACTGAAGAAGC
TGAAACAAGAGGTTGGAGAACAGAGTCACCCTGAAATCCAGCAGCCTCCCCCAGGC
CCTGAGTCCTATGTACCCCCATACCGCCCCAGCCTGGAGGAGGACAGCGCCAGCCT
GTCTGGGGAGAGTCTGGATGGACATTTGCAGGCTGTGGGGTCATGTCCAAGGCTGA
CGCCGCCCCCTGCTGACCTGCCTCTGGCATTGCCAGCCCATGGGCTATGGAGCCGAC
ACATCCTGCAGCAGACACTGATGGACGAGGGGCTGCGGCTCGCCCGCCTCGTCTCCC
ACGACCGCGTGGGCCGCCTCAGCCCCTGTGTGCCTGCGAAGCCACCTCTCGCAGCTG
AACAGATGGGTAAGGATGGCAGGGGTTATGTCCCAGCTACCATCAAGATGACCGTG
GAAAGGGACCAACCACTTCCTACCCCAGAGCTCCAGATGCCTACCATGGTGCCTTCT
TATGACCTTGGAATGGCCCCTGATTCCTCCATGAGCATGCAGCTTGGCCCAGATATG
GTGCCCCAGGTGTACCCACCACACTCTCACTCCATCCCCCCGTATCAAGGCCTCTCC
CCAGAAGAATCAGTCAACGTGTTGTCAGCCTTCCAGGAGCCTCACCTGCAGATGCCC
CCCAGCCTGGGCCAGATGAGCCTGCCCTTTGACCAGCCTCACCCCCAGGGCCTGCTG
CCGTGCCAGCCTCAGGAGCATGCTGTGTCCAGCCCTGACCCCCTGCTCTGCTCAGAT
GTGACCATGGTGGAAGACAGCTGCCTGAGCCAGCCAGTGACAGCGTTTCCTCAGGG
CACTTGGATTGGTGAAGACATATTCCCTCCTCTGCTGCCTCCCACTGAACAGGACCT
CACTAAGCTTCTCCTGGAGGGGCAAGGGGAGTCGGGGGGAGGGTCCTTGGGGGCAC
AGCCCCTCCTGCAGCCCTCCCACTATGGGCAATCTGGGATCTCAATGTCCCACATGG
ACCTAAGGGCCAACCCCAGTTGGTGATCCCAGCTGGAGGGAGAACCCAAAGAGACA
GCTCTTCTACTACCCCCACAGACCTGCTCTGGACACTTGCTCATGCCCTGCCAAGCA
GCAGATGGGGAGGGTGCCCTCCTATCCCCACCTACTCCTGGGTCAGGAGGAAAAGA
CTAACAGGAGAATGCACAGTGGGTGGAGCCAATCCACTCCTTCCTTTCTATCATTCC

CCTGCCCACCTCCTTCCAGCACTGACTGGAAGGGAAGTTCAGGCTCTGAGACACACC
CCAACATGCCTGCACCTGCAGCGCACACGCACGCACACACACATACAGAGCTCT
CTGAGGGTGATGGGGCTGAGCAGGAGGGGGGCTGGGTAAGAGCACAGGTTAGGGC
ATGGAAGGCTTCTCCGCCCATTCTGACCCAGGGCCTAGGACGGATAGGCAGGAACA
TACAGACACATTTACACTAGAGGCCAGGGATAGAGGATATTGGGTCTCAGCCCTAG
GGGAATGGGAAGCAGCTCAAGGGACCCTGGGTGGGAGCATAGGAGGGGTCTGGAC
ATGTGGTTACTAGTACAGGTTTTGCCCTGATTAAAAAATCTCCCAAAGCCCCAAATT
CCTGTTAGCCAGGTGGAGGCTTCTGATACGTGTATGAGACTATGCAAAAGTACAAG
GGCTGAGATTCTTCGTGTATAGCTGTGTGAACGTGTATGTACCTAGGATATGTTAAA
TGTATAGCTGGCACCTTAGTTGCATGACCACATAGAACATGTGTCTATCTGCTTTTGC
CTACGTGACAACACAAATTTGGGAGGGTGAGACACTGCACAGAAGACAGCAGCAAG
TGTGCTGGCCTCTCTGACATATGCTAACCCCCAAATACTCTGAATTTGGAGTCTGACT
GTGCCCAAGTGGGTCCAAGTGGCTGTGACATCTACGTATGGCTCCACACCTCCAATG
CTGCCTGGGAGCCAGGGTGAGAGTCTGGGTCCAGGCCTGGCCATGTGGCCCTCCAG
TGTATGAGAGGGCCCTGCCTGCTGCATCTTTTCTGTTGCCCCATCCACCGCCAGCTTC
CCTTCACTCCCCTATCCCATTCTCCCTCTCAAGGCAGGGGTCATAGATCCTAAGCCAT
AAAATAAATTTTATTCCAAAATAACAAAATAAATAATCTACTGTACACAATCTGAAA
A

Blue: NAB2 exons 1-6
Green: STAT6 exons 17-22
Red: ASO IONIS993523 target site

Table S4. Moffitt-ns NAB2exon5-STAT6exon16 mRNA transcript sequence:

AGACAGAGGCGCGGAGGCTCGGAGAGAGAAGACGTGGAGGGAGGGACAGAGCCTG
GACAGCGGTGGACACGGCATCGTGCGCGGGGAAGAGGGCAGCACGCAGCAGGCGC
CGAGCGCCGGGCACCGAGAAGGGCAGCCCGGGTGATCTCCGGCCGTCCATGCACAG
AGCGCCTTCCCCCACAGCCGAGCAGCCGCCGGGCGGAGGGGACAGCGCCCGCCGGA
CCCTGCAGCCCAGACTCAAGCCCAGTGCCCGAGCCATGGCACTGCCTCGGACGCTG
GGGGAGCTGCAGCTGTACCGGGTCCTGCAGCGCGCCAACCTCCTTTCCTACTATGAG
ACCTTCATCCAGCAGGGAGGGGACGACGTGCAGCAGCTGTGTGAGGCGGGTGAGGA
GGAGTTTCTGGAGATCATGGCACTTGTGGGCATGGCCACCAAGCCCCTCCATGTCCG
GCGCCTGCAGAAGGCACTGAGAGAGTGGGCCACCAATCCAGGGCTCTTCAGTCAAC
CAGTGCCTGCTGTTCCCGTCTCCAGCATCCCGCTCTTCAAGATCTCTGAGACTGCGG
GTACCCGGAAAGGGAGCATGAGCAATGGGCATGGCAGCCCAGGGGAAAAGGCAGG
CAGTGCCCGCAGTTTTAGCCCCAAGAGCCCCCTTGAACTTGGAGAGAAGCTATCACC
ACTGCCTGGGGGACCTGGGGCAGGGGACCCCCGGATCTGGCCAGGCCGGAGCACTC
CAGAGTCGGACGTTGGGGCAGGAGGAGAAGAGGAGGCTGGCTCGCCCCCCTTCTCC
CCCCCTGCAGGGGGAGGAGTCCCTGAGGGGACTGGGGCTGGGGGGCTGGCAGCAG
GTGGGACTGGGGGTGGTCCAGACCGACTGGAGCCAGAGATGGTACGCATGGTGGTG
GAAAGTGTGGAGAGGATCTTCCGGAGCTTCCCAAGGGGGGATGCTGGGGAGGTCAC
ATCCCTGCTAAAGCTGAATAAGAAGCTGGCACGGAGCGTTGGGCACATCTTTGAGA
TGGATGATAATGACAGCCAGAAGGAAGAGGAGATCCGCAAATACAGCATCATCTAT
GGCCGTTTCGACTCTAAGCGGCGGGAGGGCAAGCAGCTCAGCCTGCACGAGCTCAC
CATCAACGAGGCTGCTGCCCAGTTCTGCATGAGGGACAACACGCTCTTATTACGGAG
AGTGGAGCTCTTCTCTTTGTCCCGCCAAGTAGCCCGAGAGAGCACCTACTTGTCCTC
CTTGAAGGGCTCCAGGCTTCACCCTGAAGAACTGGGAGGCCCTCCACTGAAGAAGC
TGAAACAAGAGGTTGGAGAACAGAGTCACCCTGAAATCCAGCAGCCTCCCCCAGGC
CCTGAGTCCTATGTACCCCCATACCGCCCCAGCCTGGAGGAGGACAGCGCCAGCCT
GTCTGGGGAGAGTCTGGATGGACATTTGCAGGGCTCTCCACAGATAGAGAACATCC
AGCCATTCTCTGCCAAAGACCTGTCCATTCGCTCACTGGGGGACCGAATCCGGGATC
TTGCTCAGCTCAAAAATCTCTATCCCAAGAAGCCCAAGGATGAGGCTTTCCGGAGCC
ACTACAAGCCTGAACAGATGGGTAAGGATGGCAGGGGTTATGTCCCAGCTACCATC
AAGATGACCGTGGAAAGGGACCAACCACTTCCTACCCCAGAGCTCCAGATGCCTAC
CATGGTGCCTTCTTATGACCTTGGAATGGCCCCTGATTCCTCCATGAGCATGCAGCTT
GGCCCAGATATGGTGCCCCAGGTGTACCCACCACTCTCACTCCATCCCCCCGTAT
CAAGGCCTCTCCCCAGAAGAATCAGTCAACGTGTTGTCAGCCTTCCAGGAGCCTCAC
CTGCAGATGCCCCCCAGCCTGGGCCAGATGAGCCTGCCCTTTGACCAGCCTCACCCC
CAGGGCCTGCTGCCGTGCCAGCCTCAGGAGCATGCTGTGTCCAGCCCTGACCCCCTG
CTCTGCTCAGATGTGACCATGGTGGAAGACAGCTGCCTGAGCCAGCCAGTGACAGC
GTTTCCTCAGGGCACTTGGATTGGTGAAGACATATTCCCTCCTCTGCTGCCTCCCACT
GAACAGGACCTCACTAAGCTTCTCCTGGAGGGGCAAGGGGAGTCGGGGGGAGGGTC
CTTGGGGGCACAGCCCCTCCTGCAGCCCTCCCACTATGGGCAATCTGGGATCTCAAT
GTCCCACATGGACCTAAGGGCCAACCCCAGTTGGTGATCCCAGCTGGAGGGAGAAC
CCAAAGAGACAGCTCTTCTACTACCCCCACAGACCTGCTCTGGACACTTGCTCATGC
CCTGCCAAGCAGCAGATGGGGAGGGTGCCCTCCTATCCCCACCTACTCCTGGGTCAG
GAGGAAAAGACTAACAGGAGAATGCACAGTGGGTGGAGCCAATCCACTCCTTCCTT
TCTATCATTCCCCTGCCCACCTCCTTCCAGCACTGACTGGAAGGGAAGTTCAGGCTC

TGAGACACACCCCAACATGCCTGCACCTGCAGCGCACACGCACGCACACACACA
TACAGAGCTCTCTGAGGGTGATGGGGCTGAGCAGGAGGGGGGCTGGGTAAGAGCAC
AGGTTAGGGCATGGAAGGCTTCTCCGCCCATTCTGACCCAGGGCCTAGGACGGATA
GGCAGGAACATACAGACACATTTACACTAGAGGCCAGGGATAGAGGATATTGGGTC
TCAGCCCTAGGGGAATGGGAAGCAGCTCAAGGGACCCTGGGTGGGAGCATAGGAG
GGGTCTGGACATGTGGTTACTAGTACAGGTTTTGCCCTGATTAAAAAATCTCCCAAA
GCCCCAAATTCCTGTTAGCCAGGTGGAGGCTTCTGATACGTGTATGAGACTATGCAA
AAGTACAAGGGCTGAGATTCTTCGTGTATAGCTGTGTGAACGTGTATGTACCTAGGA
TATGTTAAATGTATAGCTGGCACCTTAGTTGCATGACCACATAGAACATGTGTCTAT
CTGCTTTTGCCTACGTGACAACACAAATTTGGGAGGGTGAGACACTGCACAGAAGA
CAGCAGCAAGTGTGCTGGCCTCTCTGACATATGCTAACCCCCAAATACTCTGAATTT
GGAGTCTGACTGTGCCCAAGTGGGTCCAAGTGGCTGTGACATCTACGTATGGCTCCA
CACCTCCAATGCTGCCTGGGAGCCAGGGTGAGAGTCTGGGTCCAGGCCTGGCCATG
TGGCCCTCCAGTGTATGAGAGGGCCCTGCCTGCTGCATCTTTTCTGTTGCCCCATCCA
CCGCCAGCTTCCCTTCACTCCCCTATCCCATTCTCCCTCTCAAGGCAGGGGTCATAGA
TCCTAAGCCATAAAATAAATTTTATTCCAAAATAACAAAATAAATAATCTACTGTAC
ACAATCTGAAAA

Blue: NAB2 exons 1-5

Green: STAT6 exons 16-22

Red: ASO IONIS993523 target site

Table S5. INT-SFT NAB2exon6-NAB2intron6-STAT6exon16 mRNA transcript sequence:

AGACAGAGGCGCGGAGGCTCGGAGAGAAGACGTGGAGGGAGGGACAGAGCCTG
GACAGCGGTGGACACGGCATCGTGCGCGGGGAAGAGGGCAGCACGCAGCAGGCGC
CGAGCGCCGGGCACCGAGAAGGGCAGCCCGGGTGATCTCCGGCCGTCCATGCACAG
AGCGCCTTCCCCCACAGCCGAGCAGCCGCCGGGCGGAGGGGACAGCGCCCGCCGGA
CCCTGCAGCCCAGACTCAAGCCCAGTGCCCGAGCCATGGCACTGCCTCGGACGCTG
GGGGAGCTGCAGCTGTACCGGGTCCTGCAGCGCGCCAACCTCCTTTCCTACTATGAG
ACCTTCATCCAGCAGGGAGGGGACGACGTGCAGCAGCTGTGTGAGGCGGGTGAGGA
GGAGTTTCTGGAGATCATGGCACTTGTGGGCATGGCCACCAAGCCCCTCCATGTCCG
GCGCCTGCAGAAGGCACTGAGAGAGTGGGCCACCAATCCAGGGCTCTTCAGTCAAC
CAGTGCCTGCTGTTCCCGTCTCCAGCATCCCGCTCTTCAAGATCTCTGAGACTGCGG
GTACCCGGAAAGGGAGCATGAGCAATGGGCATGGCAGCCCAGGGGAAAAGGCAGG
CAGTGCCCGCAGTTTTAGCCCCAAGAGCCCCCTTGAACTTGGAGAGAAGCTATCACC
ACTGCCTGGGGGACCTGGGGCAGGGGACCCCCGGATCTGGCCAGGCCGGAGCACTC
CAGAGTCGGACGTTGGGGCAGGAGGAGAAGAGGAGGCTGGCTCGCCCCCCTTCTCC
CCCCCTGCAGGGGGAGGAGTCCCTGAGGGGACTGGGGCTGGGGGGCTGGCAGCAG
GTGGGACTGGGGGTGGTCCAGACCGACTGGAGCCAGAGATGGTACGCATGGTGGTG
GAAAGTGTGGAGAGGATCTTCCGGAGCTTCCCAAGGGGGGATGCTGGGGAGGTCAC
ATCCCTGCTAAAGCTGAATAAGAAGCTGGCACGGAGCGTTGGGCACATCTTTGAGA
TGGATGATAATGACAGCCAGAAGGAAGAGGAGATCCGCAAATACAGCATCATCTAT
GGCCGTTTCGACTCTAAGCGGCGGGAGGGCAAGCAGCTCAGCCTGCACGAGCTCAC
CATCAACGAGGCTGCTGCCCAGTTCTGCATGAGGGACAACACGCTCTTATTACGGAG
AGTGGAGCTCTTCTCTTTGTCCCGCCAAGTAGCCCGAGAGAGCACCTACTTGTCCTC
CTTGAAGGGCTCCAGGCTTCACCCTGAAGAACTGGGAGGCCCTCCACTGAAGAAGC
TGAAACAAGAGGTTGGAGAACAGAGTCACCCTGAAATCCAGCAGCCTCCCCCAGGC
CCTGAGTCCTATGTACCCCCATACCGCCCCAGCCTGGAGGAGGACAGCGCCAGCCT
GTCTGGGGAGAGTCTGGATGGACATTTGCAGGCTGTGGGGTCATGTCCAAGGCTGA
CGCCGCCCCCTGCTGACCTGCCTCTGGCATTGCCAGCCCATGGGCTATGGAGCCGAC
ACATCCTGCAGCAGACACTGATGGACGAGGGGCTGCGGCTCGCCCGCCTCGTCTCCC
ACGACCGCGTGGGCCGCCTCAGCCCCTGTGTGCCTGCGAAGCCACCTCTCGCAGCTC
CTGACAGAACCAGAAATTCCAGCGCAACCGAGTTGCAGCTCAAAAATCTCTATCCC
AAGAAGCCCAAGGATGAGGCTTTCCGGAGCCACTACAAGCCTGAACAGATGGGTAA
GGATGGCAGGGGTTATGTCCCAGCTACCATCAAGATGACCGTGGAAAGGACCAAC
CACTTCCTACCCCAGAGCTCCAGATGCCTACCATGGTGCCTTCTTATGACCTTGGAA
TGGCCCCTGATTCCTCCATGAGCATGCAGCTTGGCCCAGATATGGTGCCCCAGGTGT
ACCCACCACACTCTCACTCCATCCCCCCGTATCAAGGCCTCTCCCCAGAAGAATCAG
TCAACGTGTTGTCAGCCTTCCAGGAGCCTCACCTGCAGATGCCCCCCAGCCTGGGCC
AGATGAGCCTGCCCTTTGACCAGCCTCACCCCCAGGGCCTGCTGCCGTGCCAGCCTC
AGGAGCATGCTGTGTCCAGCCCTGACCCCCTGCTCTGCTCAGATGTGACCATGGTGG
AAGACAGCTGCCTGAGCCAGCCAGTGACAGCGTTTCCTCAGGGCACTTGGATTGGTG
AAGACATATTCCCTCCTCTGCTGCCTCCCACTGAACAGGACCTCACTAAGCTTCTCCT
GGAGGGGCAAGGGGAGTCGGGGGGAGGGTCCTTGGGGGCACAGCCCCTCCTGCAG
CCCTCCCACTATGGGCAATCTGGGATCTCAATGTCCCACATGGACCTAAGGGCCAAC
CCCAGTTGGTGATCCCAGCTGGAGGGAGAACCCAAAGAGACAGCTCTTCTACTACC
CCCACAGACCTGCTCTGGACACTTGCTCATGCCCTGCCAAGCAGCAGATGGGGAGG

GTGCCCTCCTATCCCCACCTACTCCTGGGTCAGGAGGAAAAGACTAACAGGAGAAT
GCACAGTGGGTGGAGCCAATCCACTCCTTCCTTTCTATCATTCCCCTGCCCACCTCCT
TCCAGCACTGACTGGAAGGGAAGTTCAGGCTCTGAGACACACCCCAACATGCCTGC
ACCTGCAGCGCGCACACGCACGCACACACACATACAGAGCTCTCTGAGGGTGATGG
GGCTGAGCAGGAGGGGGGCTGGGTAAGAGCACAGGTTAGGGCATGGAAGGCTTCTC
CGCCCATTCTGACCCAGGGCCTAGGACGGATAGGCAGGAACATACAGACACATTTA
CACTAGAGGCCAGGGATAGAGGATATTGGGTCTCAGCCCTAGGGGAATGGGAAGCA
GCTCAAGGGACCCTGGGTGGGAGCATAGGAGGGGTCTGGACATGTGGTTACTAGTA
CAGGTTTTGCCCTGATTAAAAAATCTCCCAAAGCCCCAAATTCCTGTTAGCCAGGTG
GAGGCTTCTGATACGTGTATGAGACTATGCAAAAGTACAAGGGCTGAGATTCTTCGT
GTATAGCTGTGTGAACGTGTATGTACCTAGGATATGTTAAATGTATAGCTGGCACCT
TAGTTGCATGACCACATAGAACATGTGTCTATCTGCTTTTGCCTACGTGACAACACA
AATTTGGGAGGGTGAGACACTGCACAGAAGACAGCAGCAAGTGTGCTGGCCTCTCT
GACATATGCTAACCCCCAAATACTCTGAATTTGGAGTCTGACTGTGCCCAAGTGGGT
CCAAGTGGCTGTGACATCTACGTATGGCTCCACACCTCCAATGCTGCCTGGGAGCCA
GGGTGAGAGTCTGGGTCCAGGCCTGGCCATGTGGCCCTCCAGTGTATGAGAGGGCC
CTGCCTGCTGCATCTTTTCTGTTGCCCCATCCACCGCCAGCTTCCCTTCACTCCCCTA
TCCCATTCTCCCTCTCAAGGCAGGGGTCATAGATCCTAAGCCATAAAATAAATTTTA
TTCCAAAATAACAAAATAAATAATCTACTGTACACAATCTGAAAA

Blue: NAB2 exons 1-6
Purple: NAB2 intron 6
Green: STAT6 exons 16-22
Red: ASO IONIS993523 target site

Table S6. IEC139 NAB2exon6-STAT6exon16 mRNA transcript sequence:

AGACAGAGGCGCGGAGGCTCGGAGAGAGAAGACGTGGAGGGAGGGACAGAGCCTG
GACAGCGGTGGACACGGCATCGTGCGCGGGGAAGAGGGCAGCACGCAGCAGGCGC
CGAGCGCCGGGCACCGAGAAGGGCAGCCCGGGTGATCTCCGGCCGTCCATGCACAG
AGCGCCTTCCCCCACAGCCGAGCAGCCGCCGGGCGGAGGGGACAGCGCCCGCCGGA
CCCTGCAGCCCAGACTCAAGCCCAGTGCCCGAGCCATGGCACTGCCTCGGACGCTG
GGGGAGCTGCAGCTGTACCGGGTCCTGCAGCGCGCCAACCTCCTTTCCTACTATGAG
ACCTTCATCCAGCAGGGAGGGGACGACGTGCAGCAGCTGTGTGAGGCGGGTGAGGA
GGAGTTTCTGGAGATCATGGCACTTGTGGGCATGGCCACCAAGCCCCTCCATGTCCG
GCGCCTGCAGAAGGCACTGAGAGAGTGGGCCACCAATCCAGGGCTCTTCAGTCAAC
CAGTGCCTGCTGTTCCCGTCTCCAGCATCCCGCTCTTCAAGATCTCTGAGACTGCGG
GTACCCGGAAAGGGAGCATGAGCAATGGGCATGGCAGCCCAGGGGAAAAGGCAGG
CAGTGCCCGCAGTTTTAGCCCCAAGAGCCCCCTTGAACTTGGAGAGAAGCTATCACC
ACTGCCTGGGGGACCTGGGGCAGGGGACCCCCGGATCTGGCCAGGCCGGAGCACTC
CAGAGTCGGACGTTGGGGCAGGAGGAGAAGAGGAGGCTGGCTCGCCCCCCTTCTCC
CCCCCTGCAGGGGGAGGAGTCCCTGAGGGGACTGGGGCTGGGGGGCTGGCAGCAG
GTGGGACTGGGGGTGGTCCAGACCGACTGGAGCCAGAGATGGTACGCATGGTGGTG
GAAAGTGTGGAGAGGATCTTCCGGAGCTTCCCAAGGGGGGATGCTGGGGAGGTCAC
ATCCCTGCTAAAGCTGAATAAGAAGCTGGCACGGAGCGTTGGGCACATCTTTGAGA
TGGATGATAATGACAGCCAGAAGGAAGAGGAGATCCGCAAATACAGCATCATCTAT
GGCCGTTTCGACTCTAAGCGGCGGGAGGGCAAGCAGCTCAGCCTGCACGAGCTCAC
CATCAACGAGGCTGCTGCCCAGTTCTGCATGAGGGACAACACGCTCTTATTACGGAG
AGTGGAGCTCTTCTCTTTGTCCCGCCAAGTAGCCCGAGAGAGCACCTACTTGTCCTC
CTTGAAGGGCTCCAGGCTTCACCCTGAAGAACTGGGAGGCCCTCCACTGAAGAAGC
TGAAACAAGAGGTTGGAGAACAGAGTCACCCTGAAATCCAGCAGCCTCCCCCAGGC
CCTGAGTCCTATGTACCCCCATACCGCCCCAGCCTGGAGGAGGACAGCGCCAGCCT
GTCTGGGGAGAGTCTGGATGGACATTTGCAGGCTGTGGGGTCATGTCCAAGGCTGA
CGCCGCCCCCTGCTGACCTGCCTCTGGCATTGCCAGCCCATGGGCTATGGAGCCGAC
ACATCCTGCAGCAGACACTGATGGACGAGGGGCTGCGGCTCGCCCGCCTCGTCTCCC
ACGACCGCGTGGGCCGCCTCAGCCCCTGTGTGCCTGCGAAGCCACCTCTCGCAGGCT
CTCCACAGATAGAGAACATCCAGCCATTCTCTGCCAAAGACCTGTCCATTCGCTCAC
TGGGGGACCGAATCCGGGATCTTGCTCAGCTCAAAAATCTCTATCCCAAGAAGCCC
AAGGATGAGGCTTTCCGGAGCCACTACAAGCCTGAACAGATGGGTAAGGATGGCAG
GGGTTATGTCCCAGCTACCATCAAGATGACCGTGGAAAGGGACCAACCACTTCCTA
CCCCAGAGCTCCAGATGCCTACCATGGTGCCTTCTTATGACCTTGGAATGGCCCCTG
ATTCCTCCATGAGCATGCAGCTTGGCCCAGATATGGTGCCCCAGGTGTACCCACCAC
ACTCTCACTCCATCCCCCCGTATCAAGGCCTCTCCCCAGAAGAATCAGTCAACGTGT
TGTCAGCCTTCCAGGAGCCTCACCTGCAGATGCCCCCCAGCCTGGGCCAGATGAGCC
TGCCCTTTGACCAGCCTCACCCCCAGGGCCTGCTGCCGTGCCAGCCTCAGGAGCATG
CTGTGTCCAGCCCTGACCCCCTGCTCTGCTCAGATGTGACCATGGTGGAAGACAGCT
GCCTGAGCCAGCCAGTGACAGCGTTTCCTCAGGGCACTTGGATTGGTGAAGACATAT
TCCCTCCTCTGCTGCCTCCCACTGAACAGGACCTCACTAAGCTTCTCCTGGAGGGGC
AAGGGGAGTCGGGGGGAGGGTCCTTGGGGGCACAGCCCCTCCTGCAGCCCTCCCAC
TATGGGCAATCTGGGATCTCAATGTCCCACATGGACCTAAGGGCCAACCCCAGTTGG
TGATCCCAGCTGGAGGGAGAACCCAAAGAGACAGCTCTTCTACTACCCCCACAGAC

CTGCTCTGGACACTTGCTCATGCCCTGCCAAGCAGCAGATGGGGAGGGTGCCCTCCT
ATCCCCACCTACTCCTGGGTCAGGAGGAAAAGACTAACAGGAGAATGCACAGTGGG
TGGAGCCAATCCACTCCTTCCTTTCTATCATTCCCCTGCCCACCTCCTTCCAGCACTG
ACTGGAAGGGAAGTTCAGGCTCTGAGACACACCCCAACATGCCTGCACCTGCAGCG
CGCACACGCACGCACACACACATACAGAGCTCTCTGAGGGTGATGGGGCTGAGCAG
GAGGGGGGCTGGGTAAGAGCACAGGTTAGGGCATGGAAGGCTTCTCCGCCCATTCT
GACCCAGGGCCTAGGACGGATAGGCAGGAACATACAGACACATTTACACTAGAGGC
CAGGGATAGAGGATATTGGGTCTCAGCCCTAGGGGAATGGGAAGCAGCTCAAGGGA
CCCTGGGTGGGAGCATAGGAGGGGTCTGGACATGTGGTTACTAGTACAGGTTTTGCC
CTGATTAAAAAATCTCCCAAAGCCCCAAATTCCTGTTAGCCAGGTGGAGGCTTCTGA
TACGTGTATGAGACTATGCAAAAGTACAAGGGCTGAGATTCTTCGTGTATAGCTGTG
TGAACGTGTATGTACCTAGGATATGTTAAATGTATAGCTGGCACCTTAGTTGCATGA
CCACATAGAACATGTGTCTATCTGCTTTTGCCTACGTGACAACACAAATTTGGGAGG
GTGAGACACTGCACAGAAGACAGCAGCAAGTGTGCTGGCCTCTCTGACATATGCTA
ACCCCCAAATACTCTGAATTTGGAGTCTGACTGTGCCCAAGTGGGTCCAAGTGGCTG
TGACATCTACGTATGGCTCCACACCTCCAATGCTGCCTGGGAGCCAGGGTGAGAGTC
TGGGTCCAGGCCTGGCCATGTGGCCCTCCAGTGTATGAGAGGGCCCTGCCTGCTGCA
TCTTTTCTGTTGCCCCATCCACCGCCAGCTTCCCTTCACTCCCCTATCCCATTCTCCCT
CTCAAGGCAGGGGTCATAGATCCTAAGCCATAAAATAAATTTTATTCCAAAATAACA
AAATAAATAATCTACTGTACACAATCTGAAAA

Blue: NAB2 exons 1-6
Green: STAT6 exons 16-22
Red: ASO IONIS993523 target site
