# Supplementary material for: STAT6-targeting antisense oligonucleotides against solitary fibrous tumor
Source: Mol Ther Nucleic Acids. 2024 Feb 15;35(2):102154. doi: 10.1016/j.omtn.2024.102154 (PMC10950871; doi:10.1016/j.omtn.2024.102154)
Supplement: Document S2. Article plus supplemental information [file mmc2.pdf]

Molecular Therapy
Nucleic Acids
Original Article

STAT6-targeting antisense oligonucleotides against solitary fibrous tumor

Yi Li,[1,2,11] Jose L. Mondaza-Hernandez,[3,4,11] David S. Moura,[3,11] Alexey S. Revenko,[5] Angelica Tolentino,[5] John T. Nguyen,[1,2] Nam Tran,[6] Clark A. Meyer,[1] Jose Merino-Garcia,[7] Rafael Ramos,[8] Davide Di Lernia,[3] Javier Martin-Broto,[3,4,9] Heather N. Hayenga,[1] and Leonidas Bleris[1,2,10]

[1]Department of Bioengineering, University of Texas at Dallas, Richardson, TX 75080, USA; [2]Center for Systems Biology, University of Texas at Dallas, Richardson, TX 75080, USA; [3]Health Research Institute Fundacion Jimenez Diaz, Universidad Autonoma de Madrid (IIS/FJD-UAM), 28049 Madrid, Spain; [4]University Hospital General de Villalba, 28400 Madrid, Spain; [5]Department of Antisense Drug Discovery, Ionis Pharmaceuticals, Inc, Carlsbad, CA 92010, USA; [6]Neurosurgical Oncology, Department of Neuro-Oncology, H. Lee Moffitt Cancer Center and Research Institute, Tampa, FL 33612, USA; [7]Pathology Department, University Hospital Fundacion Jimenez Diaz, Universidad Autonoma, Av. Reyes Catolicos 2, 28040 Madrid, Spain; [8]Pathology Department, University Hospital Son Espases, 07120 Palma de Mallorca, Spain; [9]Medical Oncology Department, University Hospital Fundacion Jimenez Diaz, 28040 Madrid, Spain; [10]Department of Biological Sciences, University of Texas at Dallas, Richardson, TX 75080, USA

Solitary fibrous tumor (SFT) is a rare, non-hereditary soft tissue sarcoma thought to originate from fibroblastic mesenchymal stem cells. The etiology of SFT is thought to be due to an environmental intrachromosomal gene fusion between NGFI-A-binding protein 2 (NAB2) and signal transducer and activator protein 6 (STAT6) genes on chromosome 12, wherein the activation domain of STAT6 is fused with the DNA-binding domain of NAB2 resulting in the oncogenesis of SFT. All NAB2-STAT6 fusion variations discovered in SFTs contain the C-terminal of STAT6 transcript, and thus can serve as target site for antisense oligonucleotides (ASOs)-based therapies. Indeed, our *in vitro* studies show the STAT6 3′ untranslated region (UTR)-targeting ASO (ASO 993523) was able to reduce expression of NAB2-STAT6 fusion transcripts in multiple SFT cell models with high efficiency (half-maximal inhibitory concentration: 116–300 nM). Encouragingly, *in vivo* treatment of SFT patient-derived xenograft mouse models with ASO 993523 resulted in acceptable tolerability profiles, reduced expression of NAB2-STAT6 fusion transcripts in xenograft tissues (21.9%), and, importantly, reduced tumor growth (32.4% decrease in tumor volume compared with the untreated control). Taken together, our study established ASO 993523 as a potential agent for the treatment of SFTs.

INTRODUCTION

Solitary fibrous tumor (SFT) is a rare nonhereditary soft tissue sarcoma that affects approximately 1 per 1 million people per year worldwide. SFTs appear as highly vascularized solid and/or cystic tumors.[1] They are thought to originate from mesenchymal stem cells with a fibroblastic phenotype.[2–4] Anatomically, the primary site of origin is ubiquitous and can occur in most places (commonly found intracranially within the meninges and extracranially within the pleura, pelvis, retroperitoneum, kidney, liver, limbs, or pancreas). SFTs have a high rate of local recurrence (65%) and widespread metastases (33%), with a proclivity to metastasize to the bones, liver, and lungs.[5–8] The median overall survival for SFT patients on the most effective systemic therapies currently available (pazopanib, bevacizumab and temozolomide, sunitinib, trabectedin, etc.) is 11–24 months.[9–11] Therefore, a targeted and efficacious systemic treatment for SFT is highly desired.

In 2013, a defining discovery was made that all SFTs have a hallmark intrachromosomal gene fusion between NGFI-A-binding protein 2 (NAB2) and signal transducer and activator protein 6 (STAT6) on chromosome 12 (region 12q13).[12,13] Since then, at least 12 distinct junctional breakpoints (between exons 2–7 on NAB2 and exons 2–22 on STAT6) of the NAB2-STAT6 fusion have been identified and are thought to account for pathological variation and tumor aggressiveness in SFTs.[14,15] The exact mechanism of action remains to be elucidated. However, it has been hypothesized that due to the nuclear localization signal and DNA-binding domain in NAB2, the NAB2-STAT6 fusion is translocated to the nucleus and functions as an oncogenic transcriptional factor. Specifically, depending on the fusion breakpoint junction, the chimeric protein produced by the NAB2-STAT6 gene fusion has at least one repressor domain from NAB2 replaced by the transactivation domain of STAT6, resulting in constitutive activation of early growth response factor 1-mediated transcription and downstream oncogenesis.[12,13,16,17] Even though the NAB2-STAT6 gene fusion alone seems

Received 30 October 2023; accepted 13 February 2024;
https://doi.org/10.1016/j.omtn.2024.102154.

[11]These authors contributed equally

Correspondence: Javier Martin-Broto, Health Research Institute Fundacion Jimenez Diaz, Universidad Autonoma de Madrid (IIS/FJD-UAM), 28049 Madrid, Spain.
E-mail:
Correspondence: Heather N. Hayenga, Department of Bioengineering, University of Texas at Dallas, Richardson, TX 75080, USA.
E-mail: heather.hayenga@utdallas.edu
Correspondence: Leonidas Bleris, Department of Bioengineering, University of Texas at Dallas, Richardson, TX 75080, USA.
E-mail:

This is an open access article under the CC BY license (http://creativecommons.org/licenses/by/4.0/).

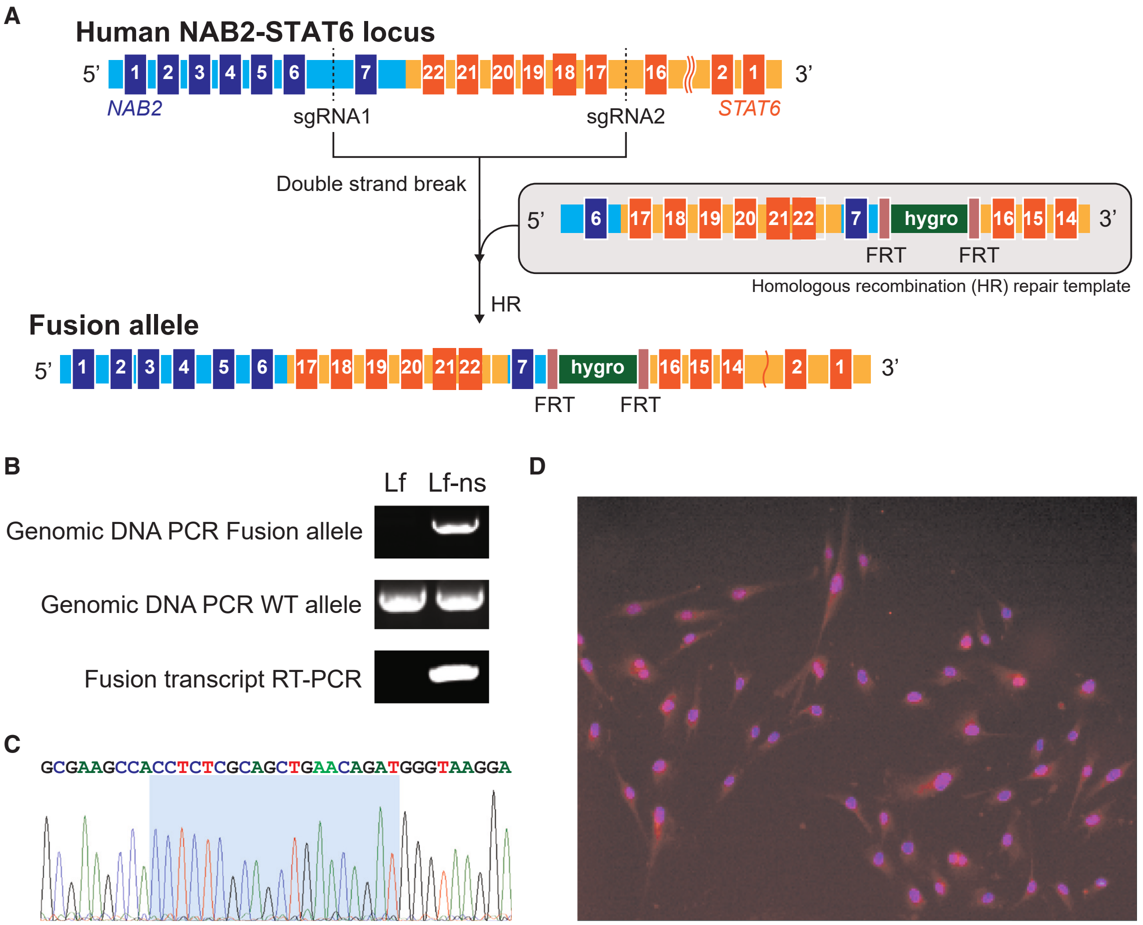

Figure 1. Generation of human Lf-ns cells using CRISPR

(A) Schematic illustration of the homologous recombination process to create the specific fusion type. (B) Long-range genomic DNA and RNA RT-PCR assays confirmed that Lf-ns cells are heterozygous. (C) Sanger sequencing result of RT-PCR amplicons confirmed the fusion type as NAB2exon6-STAT6exon17. (D) ICC assay showed prominent nuclear expression of NAB2-STAT6 fusion protein. (Red) STAT6 expression by anti-STAT6 antibody conjugated with Alexa Fluor 594, (blue) nuclear staining by DAPI.

to drive the cancer, therapeutic options and most importantly, targeted studies and clinical trials remain elusive.[10]

Antisense oligonucleotides (ASOs) are synthetic single-stranded nucleic acid sequences (typically 15–25 bp in length) that can regulate expressions of their mRNA targets via two major pathways: an RNase H-dependent mechanism that consists mainly of DNA or both RNA and DNA ASOs, and an RNase H-independent mechanism that mainly consists of RNA ASOs. In the RNase H-dependent mechanism, DNA-based ASOs bind to their complementary target mRNAs, and RNase H recognizes the DNA-RNA heteroduplex and subsequently catalyzes the degradation of the mRNA.[18–20] Therefore, ASOs could serve as useful therapeutic agents for the treatment of diseases caused by the overexpression of certain genes. By 2021, 14 ASOs had been approved by the U.S. Food and Drug Administration that target diverse genetic and rare diseases, including Duchenne muscular dystrophy, spinal muscular atrophy, and hypercholesterolemia.[21–29]

In this study, we evaluate a STAT6 3′ UTR-targeting, single-stranded DNA ASOs, with the objective to downregulate the expression of all NAB2-STAT6 fusion transcripts, in an effort to exert anti-tumor benefits for SFTs. Importantly, this therapy can apply broadly to SFT patients; all NAB2-STAT6 fusion types contain the C-terminal of STAT6 transcript.

RESULTS

Generation of SFT cell models

We previously established an SFT cell line (NS-poly) by editing the NAB2exon6-STAT6exon17 fusion into human colon cancer cells (HCT116)[30] using the CRISPR-SpCas9 system.[31–36] However, SFTs are believed to originate from mesenchymal stem cells with fibroblastic differentiation. Therefore, we developed a human telomerase reverse transcriptase (hTERT)-immortalized lung fibroblast-based NAB2exon6-STAT6exon17 (Lf-ns) cell line using the same CRISPR-mediated knock-in strategy as for the NS-poly cell model (Figure 1A). Next, to determine the genotype of Lf-ns cells, we

Table 1. Engineered and primary SFT cell models that were used in this study

ID	Description	Origin/Primary Location	NAB2-STAT6 fusion type	Matching *in vivo* mouse model
Lf-ns	hTERT-immortalized Lf-based SFT cell model	CRISPR-mediated knock-in strategy	NAB2exon6-STAT6exon17	no
Moffitt-ns	primary SFT cell line established at Moffitt Cancer Center	isolated from SFT patient (brain)	NAB2exon5-STAT6exon16	no
INT-SFT	primary SFT cell line established at Oncology Referral Center (Centro di Riferimento Oncologico)	isolated from SFT patient (intra-abdominal pelvic)	NAB2exon6-NAB2intron6-STAT6exon16	no
IEC139	primary SFT cell line established at Advanced Therapies and Biomarkers in Sarcomas (ATBSarc)	isolated from SFT patient (mesogastrium)	NAB2exon6-STAT6exon16	yes

harvested genomic DNA and performed long-range PCR using forward primer P1 (within exon 6 of NAB2) and reverse primer P2 (within exon 17 of STAT6) (Table S1). As shown in Figure 1B, the expected amplicon (1,194 bp) was observed in Lf-ns cells, but not in the parental immortalized Lf. Next, the genomic DNA was subjected to long-range PCRs using primers designed for wild-type alleles (forward primer P3: within exon 6 of NAB2; and reverse primer P4: within intron 6 of NAB2). The expected amplicon (1,020 bp) was observed in both Lf-ns and Lf cells. Taken together, these results indicated that our Lf-ns stable cells were heterozygous for both the genomic NAB2-STAT6 fusion and wild-type STAT6.

To confirm the presence of the fusion at the RNA-level, we harvested the total RNA from stable fibroblasts and performed RT-PCR for the fusion transcript (441 bp, forward primer P5: within exon 5 of NAB2; reverse primer P6: within exon 19 of STAT6). Consistent with results from our long-range PCRs, an expected NAB2-STAT6 fusion transcript band was observed in the Lf-ns cells, but not Lf cells (Figure 1B). The intended fusion breakpoint was further confirmed through sanger sequencing (Figure 1C) (breakpoint adjacent region between NAB2 exon 6: 5′-CCTCTCGCAG-3′ and STAT6 exon 17: 5′-CTGAACA GAT-3′, highlighted in blue). Next, we performed immunocytochemistry (ICC) on Lf-ns cells using an anti-STAT6 antibody conjugated with Alexa Fluor 594. As shown in Figure 1D, prominent nuclear expression of STAT6 fusion was observed. This result was consistent with previous reports, which show the STAT6 protein is predominately translocated into the nucleus upon fusion with NAB2.[12]

In addition to developing genetically engineered SFT cells, we procured three primary SFT cell lines. The primary cell lines were harvested and isolated from resected SFT tumor biospecimens from three individuals with different NAB2-STAT6 fusion subtypes (Table 1). Successful establishment of primary SFT cells was confirmed by continual expression of the SFT-identifying NAB2-STAT6 fusion. As an example, for primary SFT cells isolated at the Moffitt Cancer Center (named Moffitt-ns), total RNA was extracted and subjected to RT-PCR using Lf cells as negative control. As shown in Figure S1A, the 396-bp PCR product from the fusion allele (forward primer P5: within exon 5 of NAB2; reverse primer P6: within exon 19 of STAT6) was only observed in the primary Moffitt-ns cells, which was subsequently validated by Sanger sequencing (Figure S1B) (breakpoint-adjacent region between NAB2 exon 5: 5′-CATTTGCAGG-3′ and STAT6 exon 16: 5′-GCTCTCCACA-3′, highlighted in gray). In addition, the PCR product for the wild-type NAB2 allele (431 bp, forward primer P5: within exon 5 of NAB2; reverse primer P7: within exon 19 of STAT6) was observed in both cell lines (Figure S1A). Taken together, these results confirmed that Moffitt-ns stable cells were heterozygous for both the NAB2exon5-STAT6exon16 fusion and the wild-type NAB2 gene transcripts. Last, similar to Lf-ns, protein analysis by ICC of the primary cells showed prominent nuclear expression of STAT6 (Figure S1C).

In vitro efficacy testing of STAT6 3′ UTR-targeting ASOs

ASOs targeting the NAB2-STAT6 fusion transcripts were designed within the C-terminal of the human STAT6 transcript, which includes the 3′ untranslated region (UTR) sequence (∼1.1 kb), since all NAB2-STAT6 fusions contain this region. In addition, previous studies have demonstrated that depletion of wild-type STAT6 may elicit beneficial effects in cancer therapeutics via inhibition of M2 macrophage differentiation.[37–40] Therefore, we designed a panel of eight STAT6 3′ UTR-targeting ASOs (Figure 2A; Table S2) to suppress the expression levels of both wild-type STAT6 and NAB2-STAT6 fusion transcripts. All STAT6 ASOs are 16 nucleotides in length and connected sequentially by phosphorothioate internucleotide linkages. All ASOs have three 2′−4′ cEt-modified ribonucleotides at both the 5′- and 3′-ends, which confers an increased affinity to the target mRNA and increased resistance to exonucleases and endonucleases within the cell. The central portion is composed of 10 deoxynucleotides, which enables RNase H1 to recognize and cleave the target RNAs in the ASO:RNA duplex.[41–49]

To evaluate the *in vitro* efficacies of these candidate ASOs against both wild-type STAT6 and NAB2-STAT6 fusion transcripts (for real-time RT-PCR, forward primer P8: within exon 16 of STAT6; reverse primer P9: within exons 17 and 18 of STAT6), Moffitt-ns cells were treated with 1 μM ASOs using gymnotic delivery (i.e., directly added to the growth medium without any transfection agents). As shown in Figure 2B, most ASOs caused a significant reduction of the target transcripts with candidates 993523, 993562, and 993563 showing the highest efficacies (62%, 60%, and 56% decrease, respectively). Next, to determine the potency of the best ASO candidate 993523, we performed dose-response assays using gymnotic delivery of 993523 and primary Moffitt-ns cells. Indeed, 993523 efficiently suppressed the expression of total STAT6 transcripts (primer recognized both wild-type STAT6 and NAB2-STAT6 fusion transcripts

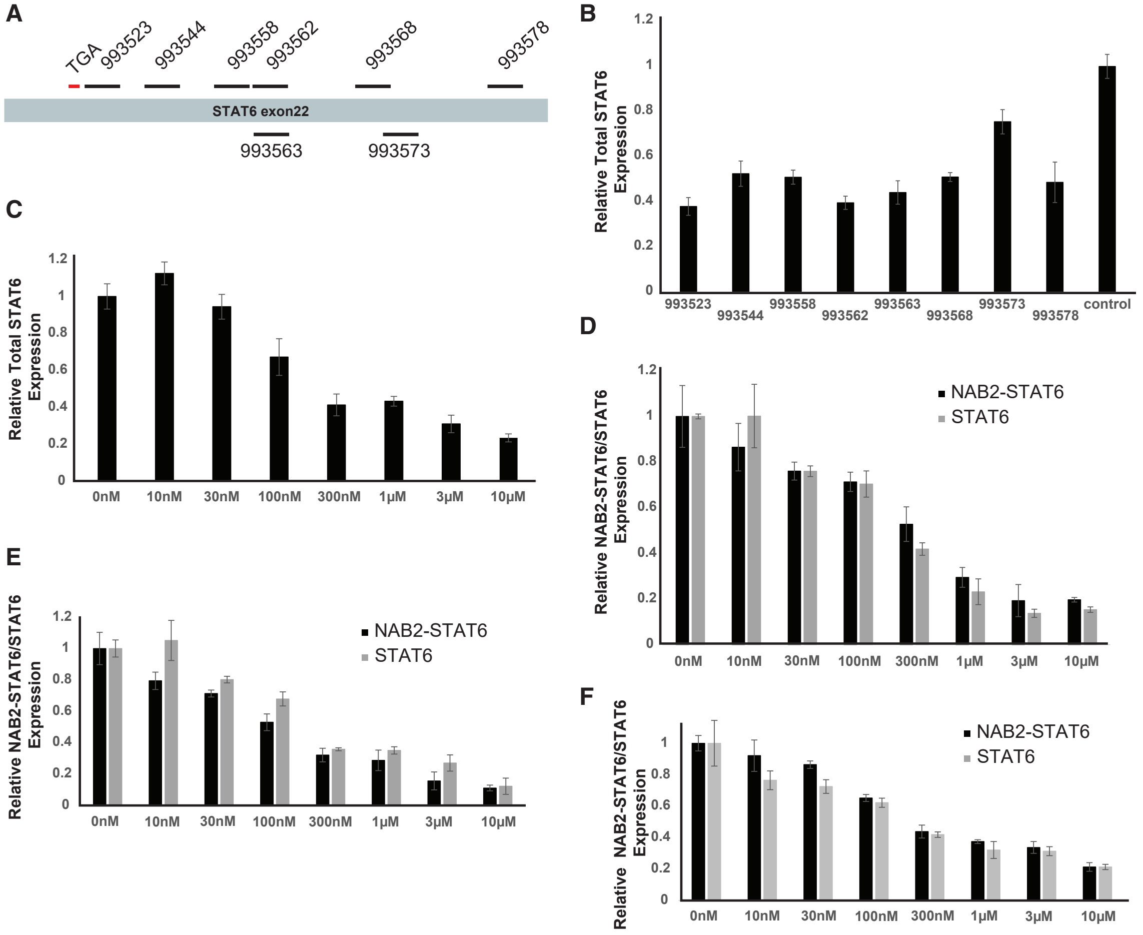

Figure 2. *In vitro* efficacy of unaided delivery of STAT6 3′ UTR-targeting ASOs

(A) Schematic illustration of where along exon 22 of STAT6 the 8 STAT6 3′ UTR-targeting ASOs bind. (B) 993523 showed the highest efficacy (62% suppression) against both wild-type (WT) STAT6 and NAB2-STAT6 fusion transcripts in Moffitt-ns cells with gymnotic delivery at concentration of 1 μM after 48 h. (C) In Moffitt-ns cells, 993523 efficiently suppressed both WT STAT6 and NAB2-STAT6 fusion transcripts (IC_{50} of 199.4 nM). 993523 efficiently suppressed both the NAB2-STAT6 fusion and WT STAT6 transcripts in (D) Lf-ns cells (IC_{50} of 299.9 nM and 206.3 nM). (E) INT-SFT cells (IC_{50} of 115.7 nM and 199.5 nM), and (F) IEC139 cells (IC_{50} of 250.8 nM and 201.7 nM).

combined) in Moffitt-ns cells (Figure 2C) (half-maximal inhibitory concentration [IC_{50}] of 199.4 nM).

To evaluate specificity and robustness of STAT6 ASOs, we further subjected the three top ASO candidates (993523, 993562, and 993563) to three additional SFT cell models (Lf-ns, INT-SFT, and IEC139). These cell lines are from different cell type backgrounds as well as NAB2-STAT6 fusion types (Table 1). Specifically, for 993523 in Lf-ns fusion cells, we designed two sets of primers. One primer set recognized only the NAB2-STAT6 fusion transcript (forward primer P10: within exon 6 of NAB2; reverse primer P11: within exon 6 of NAB2 and exon 17 of STAT6), and the other primer only recognized the wild-type STAT6 transcript (forward primer P12: within exon 16 of STAT6; reverse primer P13: within exon 17 of STAT6). As shown in Figure 2D, both transcripts were suppressed by 993523 with comparable potency (IC_{50} of 299.9 nM for the NAB2-STAT6 fusion transcript, and IC_{50} of 206.3 nM for STAT6 transcript). Similar results were also observed in INT-SFT cells (Figure 2E), which showed an IC_{50} of 115.7 nM for the NAB2-STAT6 fusion transcript (forward primer P14: within exon 6 of NAB2; reverse primer P15: within exon 16 of STAT6), and an IC_{50} of 199.5 nM for STAT6 transcript (forward primer P16: within exon 15 of STAT6; reverse primer P17: within exon 16 of STAT6), as well as in the IEC139 cells (Figure 2F) (same primer sets as in INF-SFT cells, IC_{50} of 250.8 nM for the NAB2-STAT6 fusion transcript, and IC_{50} of 201.7 nM for STAT6 transcript). Taken together, our results demonstrated that 993523, and to a lesser extent 993562 and 993563, can efficiently target both wild-type STAT6 and NAB2-STAT6 fusion transcripts in SFT cells (Table 2). Moreover, these studies prove that both STAT6 and NAB2-STAT6 fusion transcripts contain the targeting site for the three candidate ASOs.

Efficient delivery of ASOs remains a challenge in the clinical setting; accordingly, various vehicles, from DNA nanostructures to exosomes,

Table 2. IC_{50} values for STAT6 3′ UTR-targeting ASO inhibition of either NAB2-STAT6 or STAT6 transcript expression in SFT cell models

IC_{50} (nM)	993523	993562	993563
Lf-ns/NAB2-STAT6	299.9	309.4	378.1
Lf-ns/STAT6	206.3	343.1	1,020.5
INT-SFT/NAB2-STAT6	115.7	1472.5	2,574.9
INT-SFT/STAT6	199.5	3451.5	706.7
IEC139/NAB2-STAT6	250.8	966.8	,2452.7
IEC139/STAT6	201.7	1,241.8	1578

have been explored.[38] Herein, using liposome RNAiMAX (Thermo Fisher Scientific), our *in vitro* transfection results showed that 40 nM of ASOs 993523 or 993562 can be efficiently delivered into IEC139 cells and subsequently downregulate the expressions of both wild-type STAT6 and NAB2-STAT6 fusion transcripts (Figures 3A and 3B). Specifically, for STAT6 the addition of 40 nM of 993523 induced an 86.6% and 79.0% decrease after 48 and 72 h, respectively, and 993562 induced an 87.5% and 85.6% decrease after 48 and 72 h, respectively. Similarly, for NAB2-STAT6 the addition of 40 nM 993523 induced an 82.8% and 79.8% decrease after 48 and 72 h, respectively, and 993562 induced an 83.3% and 81.3% decrease after 48 and 72 h, respectively. More important, downregulation of NAB2-STAT6 and STAT6 transcripts by 40 nM of 993523 resulted in a 38.9% and 41.0% decrease in cell proliferation at 48 and 72 h, respectively, compared with the control ASO (Figure 3C). These results highlight the ASOs anti-tumor potential.

Characterization of the IEC139 patient-derived xenograft mouse model

Tumor tissues used in the IEC139 patient-derived xenograft (PDX) mouse model were originally resected from a patient with SFT (NAB2exon6-STAT6exon16 fusion type) in the mesogastrium (signed consent in accordance with the institutional guidelines of the biobank at the University Hospital Virgen del Rocio). In addition to the development of an IEC139 PDX mouse model (see “materials and methods/generation of IEC139 PDX mouse model”) we also established a matching IEC139 primary SFT cell line (Table 1).[50] To identify the NAB2-STAT6 fusion type of the IEC139 PDX model, total RNA was extracted from resected PDX tumor samples and subjected to RT-PCR and Sanger sequencing using primers specific to the fusion type (forward primer P18: within exon 6 of NAB2; reverse primer P19: within exon 16 of STAT6). As shown in Figures 4A and 4B, the IEC139 cell line and the corresponding PDX samples were confirmed to have a NAB2exon6-STAT6exon16 fusion type (breakpoint between NAB2 exon 6: 5′-CACCTCTCGCAG-3′ and STAT6 exon 16: 5′-GCTCTCCAC-3′, highlighted in blue). Importantly, histological analysis of the PDX tumor was consistent with the clinical presentation of SFT, showing proliferation of spindle and epithelioid cells with marked atypia and nuclear pleomorphism, arranged randomly on a fibrous stroma with necrotic areas (Figure 4C). Similar to pathological cases of SFT, expanded and branched vessels containing non-atypical endothelial cells were also observed. We note that the high mitotic index (25 mitoses/mm^2) together with atypia and pleomorphism are indicative of a high-grade SFT (Figures 4C and S2).[51] Finally, immunohistochemistry (IHC) demonstrated intense nuclear positivity of STAT6 and diffuse expression of CD34 (Figures 4D, 4E, and S2).[52] To our knowledge, this is the only xenograft animal model available for non-dedifferentiated SFT.

In vivo tolerability testing of STAT6 3′ UTR-targeting ASOs

Some high-affinity ASOs have been reported to induce hepatoxicity and immune system activation *in vivo*, potentially due to their immunostimulatory nature or hybridization-mediated off-target effects.[53–55] To probe the *in vivo* tolerability of our three candidate STAT6 3′ UTR-targeting ASOs (993523, 993562, and 993563), four female C57BL/6 mice per ASO group were treated with intraperitoneal injections of 50 mg/kg ASO twice a week for up to 24 days (Figure 5A). Next, the animals were evaluated using three metrics: body weight, tissue weight, and level of serum biomarkers. As shown in Figure 5B, all three candidate SFT-ASOs did not affect body weights, compared with the PBS control. Similarly, organ weights (liver, kidney, and spleen) were not significantly affected (Figure 5C). Finally, to evaluate ASO-induced hepatotoxicity, serum levels of biomarkers alanine transaminase (ALT), aspartate transaminase (AST), albumin, and total bilirubin were measured.[56] As shown in Figure 5D, no significant increases were observed in 993562- and 993563-treated groups for all four biomarkers, and only a moderate but not clinically precluding increase in serum ALT and AST levels (∼3- to 4-fold compared with PBS control) was observed in the 993523-treated group. Taken together, these results indicated that the selected SFT-ASOs 993523, 993562, and 993563 displayed acceptable *in vivo* tolerability profiles.

In vivo efficacy testing of the STAT6 3′ UTR-targeting ASOs

To evaluate *in vivo* efficacies of candidate ASOs, 993523 and 993562, 8- to 10-week-old female Foxn1^{nu} athymic nude mice bearing IEC139 PDX were randomized to receive either a treatment ASO or control ASO (792169, which has no targets within the human transcriptome but the same DNA backbone modifications) when the tumor volume reached 150–200 mm^3. Efficient delivery of ASOs (control and STAT6) to the tumor tissue was confirmed by IHC staining using anti-ASO primary antibody[57] (Ionis) (Figure S2-ASO panel). Next, as shown in Figure 6A, by day 17, mice treated with 993523 demonstrated a significant decrease in tumor volume compared with the control group. By completion of the study (day 24), the tumor volume for the 993523-treated (530.2 ± 115.9 mm^3) was on average 32.4% smaller than the control-treated tumors (784.7 ± 199.4 mm^3) (Figure 6B). Similarly, tumor weights measured at the end of treatment (day 24) were significantly lower in the 993523 group (348 ± 117 mg) compared with the control group (487 ± 90 mg) (Figure 6C). In contrast, treatment with ASO 993562 did not have any statistically significant effects on either tumor volumes or weights (Figures 6A and 6C).

Moreover, ASO 993523-treated tumor tissues had a significant reduction (21.9%) in RNA expression of the NAB2-STAT6 fusion

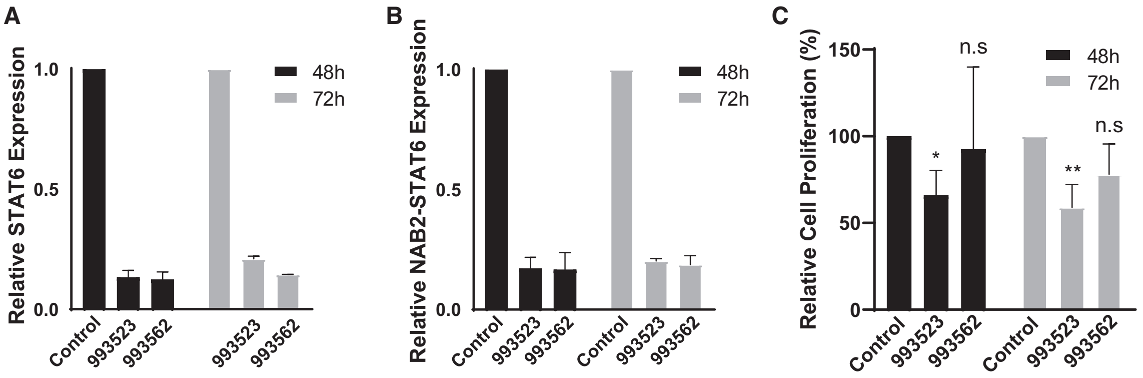

Figure 3. *In vitro* efficiency of liposome-mediated delivery of STAT6 3′ UTR-targeting ASOs
RNAiMAX-mediated delivery of 40 nM of ASOs 993523 or 993562 efficiently decreased the expression of (A) STAT6 and (B) NAB2-STAT6 fusion transcripts in IEC139 cells at both 48 and 72 h. (C) RNAiMAX-mediated delivery of ASO 993523 suppressed IEC139 cell proliferation at both 48 and 72 h. For statistical analysis, two-tailed t-tests were conducted. $^{*}p < 0.05$; $^{**}p < 0.01$; n.s., no significant difference.

transcript compared with control ASO treated tumors (Figure 6D). In contrast, wild-type STAT6 was not significantly downregulated (Figure S3). In addition, evaluation of IHC Ki67 staining revealed significant less proliferation in the 993523-treated tumors (88.13 ± 5.63) as compared with the control group (93.63 ± 3.88) (Figures 6E and S2/Ki-67 panel). These results are corroborative with the smaller tumor sizes (Figure 6B) and anti-proliferation effects of ASO 993523 observed in our *in vitro* assays (Figure 3C). We note that treatment of ASO 993562 also induced smaller, albeit statistically insignificant, Ki67 score (88.31 ± 9.92; $p = 0.1447$), as well as lower expression of the NAB2-STAT6 fusion transcript (decrease of 30.9%).

We want to emphasize that our data demonstrated a clear positive correlation between relative NAB2-STAT6 fusion transcript expression level and either tumor volume (Figure 6F) (Pearson correlation coefficient, 0.58) or Ki67 score (Figure 6G) (Pearson correlation coefficient, 0.44). These results, together with observations that no significant differences in STAT6 expression were found between the treatment groups (Figure S3), further support an oncogenic role of the NAB2-STAT6 fusion transcript in SFT.

Finally, consistent with our earlier *in vivo* tolerability testing results (Figure 5), no statistically significant body weight fluctuations were observed throughout the treatment period for any of the STAT6 3′ UTR-targeting ASOs (Figure S4). Taken together, our results demonstrate that 993523 can safely exert anti-tumor effects in SFT PDX models.

DISCUSSION

In this study, we have shown that STAT6 3′ UTR-targeting ASO 993523 effectively decreased the expression of NAB2-STAT6 fusion transcripts both *in vitro* and *in vivo* and, importantly, exerted anti-tumor impact in the SFT PDX model. These effects were likely attributed to suppressed cell proliferation, as evidenced by both inhibition of cell proliferation in the 993523-transfected SFT cells (Figure 3) and reduction of Ki67-positive cells in the 993523-treated SFT PDX model (Figure 6). It should be noted that, although NAB2-STAT6 fusion accounts for almost all known SFT cases, recently we have reported one rare SFT case in which a different fusion type (NFIX-STAT6) was observed.[58] Nevertheless, our current ASO design strategy, which targets the 3′ UTR of STAT6, should also treat those SFT fusions.

Broadly speaking, clinical adoption of ASOs poses challenges, primarily due to concerns surrounding their safety attributes and their capacity to effectively combat tumors. Regarding safety, herein we show STAT6 ASOs have a satisfactory safety profile in mice PDX models. Furthermore, recent studies using other STAT6-targeting ASOs have similarly reported the absence of toxicity in their mouse models, whether delivered with exosomes or alone.[38,59] That said, it is important to highlight that our candidate ASO 993523 exhibits the ability to target both NAB2-STAT6 fusion and the conventional wild-type STAT6 transcripts (as illustrated in Figure 2) and the consequences of inhibiting the wild-type STAT6 transcripts are yet to be understood fully. To address this concern, for each variant of the NAB2-STAT6 fusion, an alternative approach could involve the design of ASOs specifically aimed at the fusion junctions. For example, in our recent publication,[30] we developed an ASO tailored to target specifically the NAB2exon6-STAT6exon17 fusion junction that, when delivered using RNAiMAX, achieved a 58% suppression of expression of NAB2-STAT6 transcript at 1 μM within 48 h. Nevertheless, it should be noted that STAT6-targeting ASO 993523 showed even greater targeting efficiency, particularly when a gymnotic delivery method was adopted. Taken together, these results shed light on the delicate balance between two competing aspects: (a) specificity for the fusion transcript to prevent potential off-target effects and (b) efficacy to ensure a therapeutic benefit.

Another clinical challenge relates to the ability of ASOs to counteract tumor growth. Although intratumor suppression of the NAB2-STAT6 fusion transcript was relatively modest (as evidenced by the 21.9% decrease seen with 993523, as shown in Figure 6C), tumor growth was still significantly slowed (Figures 6A and 6B). This

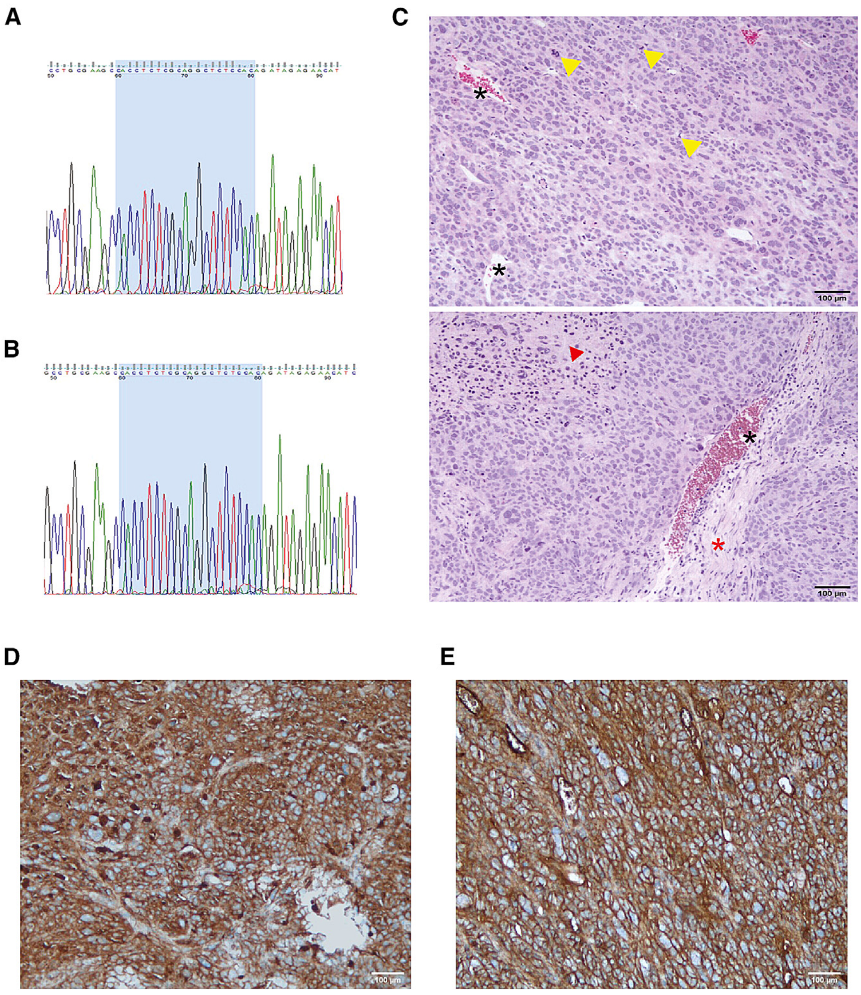

Figure 4. Characterization of the IEC139 primary cells and PDX models

The NAB2exon6-STAT6exon16 fusion type was confirmed by harvesting the total RNA from (A) IEC139 primary cells and (B) PDX tumors and subsequent RT-PCR and Sanger sequencing. (C) Hematoxylin and eosin staining of PDX tumor tissues showed hypercellular proliferation of fusiform cells, with enlarged "staghorn" blood vessels (black asterisks) and collagenous components (red asterisks). A grade III SFT was indicated by the high number of mitoses (yellow arrowheads), marked atypia, nuclear pleomorphism, and necrotic areas (red arrowheads). (D) IHC assays showed strong nuclear staining for STAT6 (brown) in the PDX tumor tissues. (E) IHC assays showed strong membrane staining for CD34 (brown) in the PDX tumor tissues.

observation suggests that the NAB2-STAT6 fusion protein plays a critical role in the expansion of SFT and, thus, could serve as an effective therapeutic target for SFTs. It is conceivable that a more pronounced decrease in the fusion transcript level, and subsequently stronger anti-tumor effects and extended survival, could be achieved by extending the treatment duration. This assumption is supported by the positive correlation between tumor volume and NAB2-STAT6 expression levels (Figure 6F), the gradual potency in tumor suppression in 993523-treated mice from day 17 onwards, as well as previous reports that showed tumor growth curves of anti-STAT6 ASO-treated mice diverging from the control group over an extended duration ($\leq$ 35 days).[38,59] Furthermore, the administration of higher dosages of ASOs is a viable option, given the low *in vivo* toxicity observed in our experiments. Subsequent investigations centered on enhancing the delivery of ASOs to the intended target and cellular uptake are needed and underway.[60]

An alternative approach to increase STAT6 ASO efficacy in treating SFTs includes the utilization of combinational therapies. ASOs have been shown to exert synergistic anti-tumor effects when used in combination with small-molecule chemotherapeutic agents. For example, patients with refractory or relapsed acute myeloid leukemia exhibited improved clinical outcomes when anti-survivin ASO (LY2181308) was administered in conjunction with cytarabine and idarubicin.[61] Our data (Figure S5) showed that the tyrosine kinase inhibitor (TKI) sitravatinib substantially suppressed the *in vitro* proliferation of IEC139 (IC_{50}, 1.1 nM). Sitravatinib is a multi-kinase inhibitor that has strong anti-angiogenic activity, while also inhibiting the function of TAMs (Tyro3, AXL, and MerTK) and DDR1.[62] Notably, sitravatinib is currently undergoing clinical evaluation for multiple indications including sarcomas.[63] Collectively, these studies suggest a combined treatment strategy involving ASO 993523 and sitravatinib or other TKIs (such as pazopanib[64,65]) could enhance the overall treatment effectiveness.

In conclusion, while the inhibitory effects related to the expression of NAB2-STAT6 fusion transcripts in 993523-treated SFT PDX model may be relatively mild, it is noteworthy that even this modest inhibition has led to a significant decrease in tumor size, highlighting the remarkable sensitivity of SFT growth to NAB2-STAT6 inhibition. Future studies investigating the mechanistic details of NAB2-STAT6 in SFT (e.g., RNA sequencing assays using ASO 993523 as perturbing agents) and validating the observed positive correlation between NAB2-STAT6 expression (at both mRNA and protein levels) and cell proliferation in a clinical cohort will be vital for advancing the field and ultimately improving outcomes for SFT patients.

MATERIALS AND METHODS

Isolation of primary Moffitt-ns cells

Primary SFT tissues were isolated at Moffitt Cancer Center under the Total Cancer Care research protocol and approved institutional review board, Institutional Biosafety and Chemical Safety Committee (IBCC),

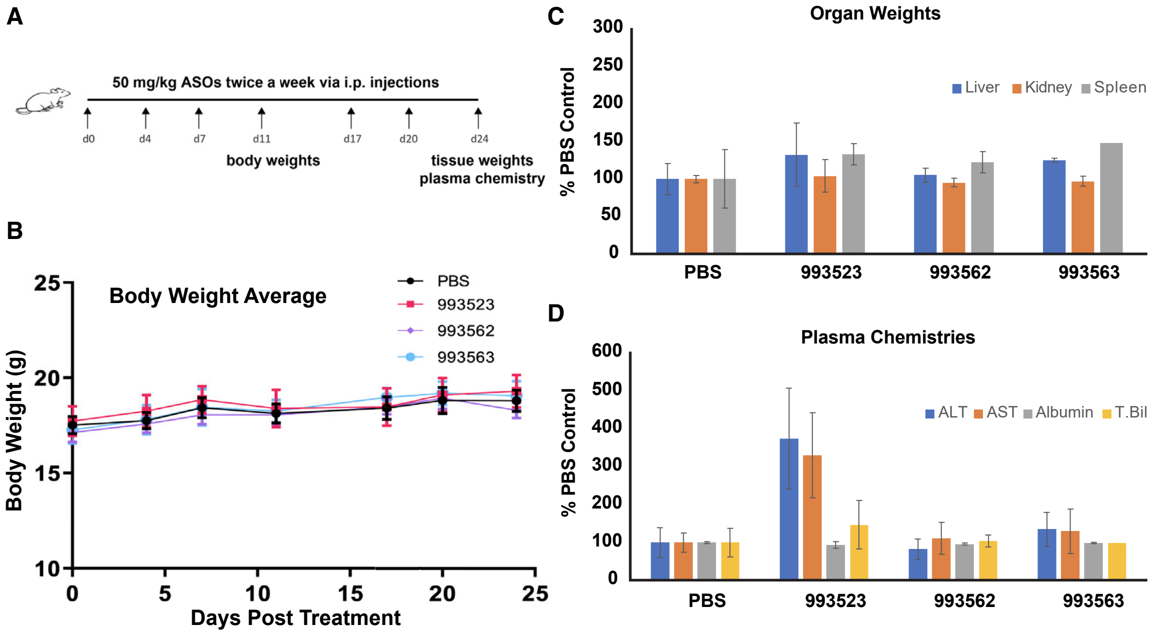

Figure 5. *In vivo* tolerability testing of STAT6 3'-UTR-targeting ASOs

(A) Schematic illustrating the injection interval and frequency for the *in vivo* tolerability testing experiments. (B) The animals' averaged body weight did not significantly change during the treatment course with ASOs 993523, 993562, and 993563 (n = 4). (C) ASOs 993523, 993562, and 993563 did not induce a significant change in the liver, kidney, or spleen of mice (n = 4). (D) ASOs 993562 and 993563 did not induce significant hepatotoxicity in mice (n = 4). ASO 993523 induced a moderate but not clinically precluding increase in serum ALT and AST levels ($p < 0.01$ using ANOVA with Dunnett's post hoc tests).

and Material Transfer Agreements (MTA) protocols. Tissues were cut into small pieces and incubated in RPMI 1640 (Gibco; catalog number: 11879020) media containing collagenase type II (1 mg/mL, Thermo Fisher Scientific; catalog number: NC9460908) and DNase I (30 U/mL, Roche; catalog number: 11284932001) for 40 min at 37°C. Next, the digested mixture was centrifuged at 1,000 rpm for 10 min at 4°C and the pellets were subjected to another round of collagenase type II/DNase I treatment. Finally, the pellet was resuspended in 10 mL RPMI-1640 media containing 10% fetal bovine serum (FBS; Corning; catalog number: 35-011-CV), 0.1 mM minimal essential medium nonessential amino acids (Invitrogen; catalog number: 11140-050), and penicillin (0.045 U/mL) and streptomycin (0.045 U/mL) (penicillin-streptomycin liquid; Invitrogen; catalog number: 15140), and subsequently maintained at 37°C with 5% CO_2.

Mammalian cell culture

The hTERT-immortalized Lf cells were acquired from the American Type Culture Collection (ATCC; catalog number: CRL-4058) and maintained at 37 °C, 100% humidity, and 5% CO_2. The cells were grown in Fibroblast Basal Medium (ATCC; catalog number: PCS-201-030) supplemented with Fibroblast Growth Kit-Low Serum (ATCC; catalog number: PCS-201-041) and 0.3 μg/mL puromycin (Gibco; catalog number: A1113803). To pass the cells, the adherent culture was first washed with PBS (Dulbecco's PBS; Mediatech; catalog number: 21-030-CM), then trypsinized with Trypsin-EDTA for Primary Cells (ATCC; catalog number: PCS-999-003), and finally diluted in fresh medium. Same protocol was used for maintaining Lf-ns cells, except that hygromycin (47.5 μg/mL; Thermo Fisher Scientific; catalog number: 10687010) was included in the complete growth medium.

The INT-SFT cell line was established using SV40 Large T Antigen-mediated immortalization by Dr. Roberta Maestro's group at Oncology Referral Center (Centro di Riferimento Oncologico). The IEC139 primary cell line was established by Dr. Javier Martin-Broto's group at Advanced Therapies and Biomarkers in Sarcomas (ATBSarc). INT-SFT and IEC139 cells were maintained in RPMI-1640 media (Thermo Fisher Scientific; catalog number: 11-875-085) containing 10% FBS (Invitrogen; catalog number: 26140), MEM non-essential amino acids (Invitrogen; catalog number: 11140–050) and 0.045 U/mL penicillin and 0.045 U/mL streptomycin (penicillin-streptomycin liquid; Invitrogen; catalog number: 15140). To pass the cells, the adherent culture was first washed with PBS (Dulbecco's PBS; Mediatech; catalog number: 21-030-CM), then trypsinized with Trypsin-EDTA (0.25% Trypsin with EDTAX4Na; Invitrogen; catalog number: 25200) and finally diluted in fresh medium.

Long-range genomic PCR and RT-PCR

For long-range genomic PCR, total genomic DNAs were harvested using DNeasy Blood & Tissue kit (Qiagen; catalog number: 69504), and long-range PCR reactions were performed using Q5 High-Fidelity 2× Master Mix (New England Biolabs; catalog number: M0492). We used 100 ng genomic DNAs as the template, and the PCR conditions were first 1 cycle of 98 °C for 30 s, followed by 40

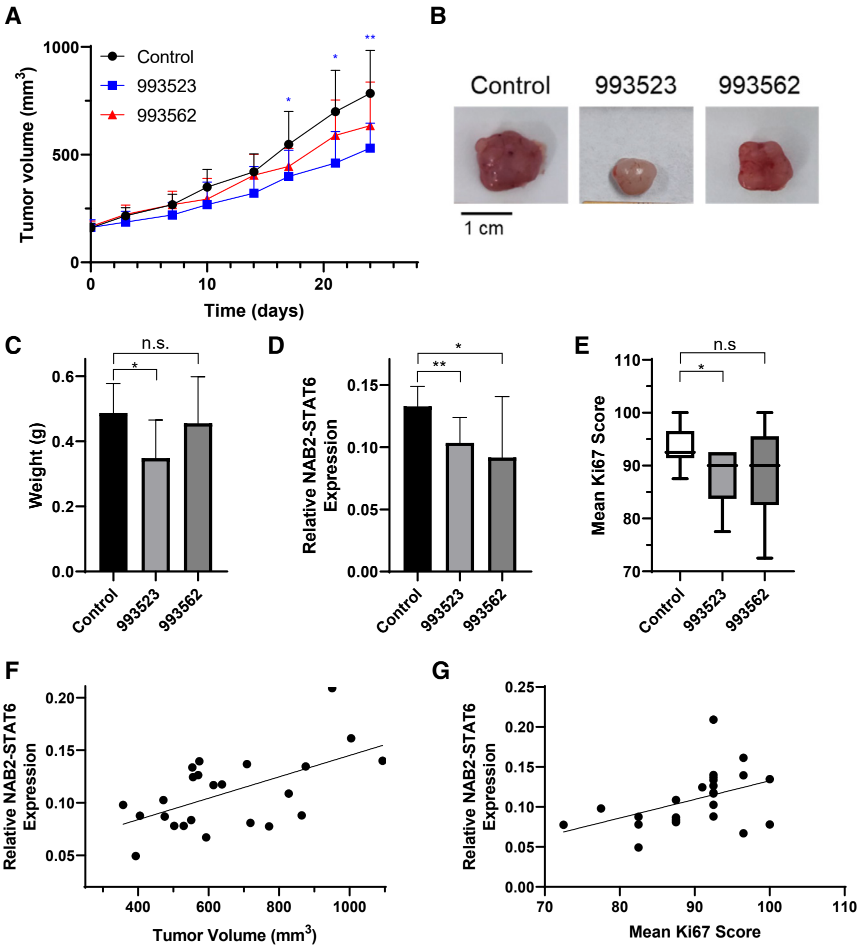

Figure 6. *In vivo* efficacy testing of STAT6 3′ UTR-targeting ASOs in IEC139 PDX mouse models
(A) Tumor volumes were significantly lower for ASO 993523-treated mice between days 17–24, compared with the untreated control tumors. No significant differences were observed for ASO 993562-treated group (n = 8). (B) Representative tumor samples for each treatment group on day 25. (C) The ASO 993523-treated group showed a significant decrease in tumor weight by day 25 (n = 8). (D) Both ASOs 993523 and 993562 demonstrated significant downregulation of the expression of NAB2-STAT6 fusion transcripts within the tumor tissues by day 25 (n = 8). (E) The ASO 993523-treated group showed a significantly lower mean Ki67 score compared with the control group. (F) Expression levels of NAB2-STAT6 fusion transcripts exhibited a positive correlation with tumor volumes (Pearson correlation coefficient: 0.58; p = 0.003). (G) Expression levels of NAB2-STAT6 fusion transcripts exhibited a positive correlation with Ki67 scores (Pearson correlation coefficient: 0.44; p value: 0.030). For statistical analysis, two-tailed t -tests were conducted. *p < 0.05; **p < 0.01; n.s., no significant difference.

cycles of 98 °C for 10 s, 66 °C for 30 s, and 72 °C for 2 min. The PCR products were subjected to 1% agarose gel electrophoresis and the DNA bands of interest were purified using QIAquick Gel Extraction Kit (Qiagen; catalog number: 28706) and subjected to direct Sanger sequencing (Genewiz) and analyzed using FinchTV (Geospiza).

For RT-PCR, total RNAs were harvested using RNeasy Mini kit (Qiagen; catalog number: 74106) and cDNAs were made using QuantiTect Reverse Transcription kit (500 ng RNA; Qiagen; catalog number: 205311). The cDNAs were then subjected to PCR reactions using Q5 High-Fidelity 2× Master Mix and the PCR conditions were: first 1 cycle of 98 °C for 30 s, followed by 40 cycles of 98 °C for 10 s, 63 °C for 30 s, and 72 °C for 1 min. The PCR products were subjected to 1% agarose gel electrophoresis and the DNA bands of interest were purified using QIAquick Gel Extraction Kit and subjected to direct Sanger sequencing (Genewiz) and analyzed using FinchTV (Geospiza).

ICC

We seeded 20,000 cells in 24-well glass plates and 16 h later, the cells were washed with 500 μL ice-cold PBS, and then treated with pre-chilled 100% methanol (Thermo Fisher Scientific; catalog number: A454) at 25 °C for 5 min. The cells were then washed three times with ice-cold PBS, and subsequently incubated with 1% BSA (Thermo Fisher Scientific; catalog number: 37525) in 1× PBS with 0.1% Triton X-100 (Sigma-Aldrich; catalog number: 93443) at 25 °C for 1 h. The cells were then incubated with Alexa Fluor 594 Anti-STAT6 antibody (1:100; Abcam; catalog number: ab207012) in 1% BSA in 1× PBST at 4 °C overnight. Next, the cells were washed with ice-cold PBS for three times, followed with incubation with DAPI (1 μg/mL in PBS; Thermo Fisher Scientific; catalog number: 62248) at 25 °C for 5 min. The cells were then washed with ice-cold PBS three times. Finally, cells were imaged using the Olympus IX81 microscopy and a Precision Control environmental chamber. The images were captured using a Hamamatsu ORCA-03 Cooled monochrome digital camera. The filter sets (Chroma) are as follows: ET390/18× (excitation) and ET460/60m (emission) for BFP, and ET560/40× (excitation) and ET630/75m (emission) for mKate. Data collection and processing was performed in the software package Slidebook 5.0.

In vitro ASO treatment

All ASOs were dissolved in PBS. ASO treatments were performed either without transfection reagents (gymnotic delivery) or using Lipofectamine RNAiMAX (Invitrogen; catalog number: 13778075). For gymnotic delivery, cells were seeded in complete growth medium and the following day, ASOs were added to the growth medium at desired final concentrations. For RNAiMAX-mediated transient transfection, cells were seeded in complete growth medium in six-well

plates (Sigma-Aldrich; catalog number: CLS3516) and the following day, 100 pmol ASOs were added according to the manufacturer's protocol. After 48 or 72 h, the cells were harvested for further analysis.

Real-time RT-PCR

For real-time RT-PCR assays, total RNAs were extracted using RNeasy Mini Kit. First-strand cDNAs were synthesized using QuantiTect Reverse Transcription kit. Next, quantitative PCR was performed using the KAPA SYBR FAST universal qPCR Kit (Kapa Biosystems; catalog number: KK4601), with GAPDH as the internal control. The forward primer (P20) for human GAPDH was 5′-AATCCCATCACCATCTTCCA-3′ and the reverse primer (P21) for human GAPDH was 5′-TGGACTCCACGACGTACTCA-3′. Quantitative analysis was performed using the $2^{-\Delta\Delta Ct}$ method. Fold-change values were reported as means with SDs.

Cell proliferation assay

IEC139 cells were seeded in complete growth medium and the following day ASOs were transiently transfected using RNAiMAX according to the manufacturer's protocol. After 48 and 72 h, cells were trypsinized with 0.25% trypsin-EDTA at 37 °C for 5 min. Trypsin-EDTA was then neutralized by adding the complete medium. The cell suspension was then counted using a hemacytometer (Paul Marienfeld GmbH & Co. KG; catalog number: 0640011). All experiments were performed in triplicate.

Generation of the IEC139 PDX mouse model

The tumor tissues for preparing IEC139 PDX mouse model were originally resected from an SFT patient (NAB2exon6-STAT6exon16 fusion type) with signed consent for the use in preclinical research. Briefly, under anesthesia and sterile conditions, a small incision on the skin was introduced in the flank area of a Foxn1^{nu} athymic nude mouse (Jackson Lab; strain number: 002019). Next, the harvested tumor fragment was placed in the cavity underneath the skin, and then the wound was stitched. This instance was considered as passage 0 of the SFT PDX mouse model. The tumor was resected when it reached the volume of 1,500 mm^3, and subsequently reimplanted in mice following the same protocol until passage 3. At this point, the model was considered as being stable, which guarantees growth upon reimplantation. For long-term storage, the harvested PDX tumors were frozen in the RPMI-1640 media containing 10% DMSO (Sigma-Aldrich; catalog number: D2650) and the tissues were found to be viable after re-thawing.

Hematoxylin and eosin staining

The paraffin-embedded tissue slides (10 μm) were first deparaffinized by heating at 60 °C for 10 min. The slides were then re-hydrated and staining using hematoxylin 560 (Leica; catalog number: 3801570). The slides were then counterstained using Eosin Y 515 (Leica; catalog number: 3801615). After dehydration, one drop of mounting medium (Abcam; catalog number: ab64230) and a glass cover were added to each slide, and slides were then observed using a Leica DMi1 Microscopy.

IHC

Paraffin-embedded tissue slides (10 μm) were deparaffinized by immersing in Xylene for 10 min, followed by sequential rehydration in anhydrous ethanol for 5 min and 96% ethanol for 5 min, and finally, in water. For automated staining, an advanced next generation stainer, the Benchmark ULTRA (Roche Diagnostics), was used with an integrated workflow. Before staining, the tissue samples underwent pretreatment using the ULTRA Cell Conditioning Solution (ULTRA CC1) buffer (Roche Diagnostics). CD34 staining required 56 min of pretreatment, while STAT6 staining required 64 min. The primary antibodies used in the staining process were prediluted and ready to use. CD34 staining was achieved using the QBEND/10 antibody (Roche Diagnostics) and required 8 min of incubation, while STAT6 staining used the EP325 antibody (Cell Marque; 1:50 dilution) with an incubation time of 32 min. Visualization of the stained samples was carried out using the OptiView DAB IHC Detection Kit (Roche Diagnostics) and Dako Autostainer Link 48 (Agilent), following the manufacturers' instructions. Ki67 staining (anti-Ki67 rabbit monoclonal antibody, Roche Diagnostics) in PDX paraffin tissue microarray was evaluated by examining multiple sections along the tumor. The percentages of Ki67-positive cells were recorded for each section, and the mean values across multiple sections were then calculated as the Ki67 final score.

Measurement of serum levels of AST, ALT, blood urea nitrogen, total bilirubin, and albumin

All four animals per group on terminal sacrifice were used for clinical chemistry assessment. Blood samples were collected from all surviving animals and put into tubes containing potassium salt of EDTA. The tubes were centrifuged (approximately 10,000 rpm, 10 min, at 4 °C) to obtain plasma. Plasma samples were processed on Olympus AU480 analyzer to obtain clinical chemistry parameters, which included AST, ALT, blood urea nitrogen, total bilirubin, and albumin.

In vivo ASO treatment

A total of 25 IEC139 PDX animals were used. Foxn1^{nu} athymic nude mice were purchased from Charles River Laboratories (catalog number: 490CRATHHO) and maintained in a pathogen-free facility, in accordance with Protocol PROEX 163.7/23 approved by the Department of Environment, Agriculture, and Interior Affairs of the Community of Madrid. Briefly, 8- to 10-week-old female Foxn1^{nu} athymic nude mice bearing IEC139 PDX were randomized to receive the treatment ASOs (ASOs 993523 and 993562) or control ASOs (792169, no targets within human transcriptome) when the tumor volume reached 150–200 mm^3. All ASOs were administered via subcutaneous injection under the neck area (50 mg/kg per injection, 2 injections per week) and the treatment lasted for a total of 24 days. During the treatment period, the animals' body weight and tumor volume were measured at the time of injection. The tumor size was measured using a digital caliper and the tumor volume was calculated as $(L \times W \times W)/2$, where L was tumor length and W the tumor width. On day 25 (1 day after the last injection), the animals were euthanized. Subsequently, tumor tissues and internal organs were weighted and then collected for further analysis.

CellTiter-Glo Luminescent Cell Viability assay

Sitravatinib was a gift from Dr. Rolf Brekken at University of Texas Southwestern. The CellTiter-Glo Luminescent Cell Viability kit was purchased from Promega (catalog number: G7573). The cell viability assays were performed according to the manufacturer's recommendations. Briefly, 3,000 IEC139 cells were seeded on 96-well plate. After 16 h, the cells were treated with sitravatinib at different final concentrations (0, 0.1, 0.3, 1, 3, 10, 30, and 100 nM) for 72 h. Next, the CellTiter-Glo reagent was added to each well and the plate was incubated for 10 min at room temperature, and subsequently the luminescence was measured using an FLUOstar Omega microplate reader (BMG Labtech; catalog number: 0415-0003). All experiments were performed in triplicate.

DATA AND CODE AVAILABILITY

Data collected for this article are available in the supplemental information.

SUPPLEMENTAL INFORMATION

Supplemental information can be found online at https://doi.org/10.1016/j.omtn.2024.102154.

ACKNOWLEDGMENTS

L.B. acknowledges funding from the U.S. National Science Foundation (NSF) grant 2114192, a Cecil H. and Ida Green Endowment, and the University of Texas at Dallas. H.H. acknowledges funding from the University of Texas at Dallas Bioengineering Transform Grant and Vice President Accelerator Award. J.M.B. acknowledges support from the SELNET project funded by the European Union's Horizon 2020 research and innovation program (grant agreement No. 825806). D.S.M. is the recipient of Sara Borrell postdoctoral fellowship from the National Institute of Health Carlos III (ISCIII) (CD20/00155). J.L.M.H. is the recipient of a PFIS predoctoral fellowship from the National Institute of Health Carlos III (ISCIII) (FI19/00184).

We thank the laboratory members in the Bleris, Hayenga, and Martin-Broto labs for their support and discussions. We also thank Dr. Rolf Brekken at the University of Texas Southwestern Medical Center for generously providing us with the chemical sitravatinib. We also thank HUVR-IbiS Biobank (Andalusian Public Health System Biobank and ISCIII-Red de Biobancos PT20/00069) for processing and staining PDX samples used in this study. We also thank the Asociación de Pacientes con Sarcomas y Tumores Raros de Aragón (APSATUR) for participation in this study.

AUTHOR CONTRIBUTIONS

Conceptualization: Y.L., J.L.M.-H., D.S.M., A.S.R., J.M.-B., C.A.M., H.N.H., and L.B.; formal analysis: Y.L., J.L.M.-H., A.S.R., D.D.L., and D.S.M.; investigation: Y.L., J.L.M.-H., D.S.M., J.T.N., N.T., J.M.-G., A.T., and A.S.R.; writing: all authors contributed to manuscript writing; supervision: J.M.-B., H.H., and L.B. All authors have read and agreed to the published version of the manuscript.

DECLARATION OF INTERESTS

We declare that we have no competing interests.

REFERENCES

1. Hayenga, H.N., Cai, C., Fetzer, D., White, S., Kuban, J., Wardak, Z., Benjamin, R.S., Pan, E., Strauss, J., Gao, B., et al. (2022). Rare Solid and Cystic Presentation of Hemangiopericytoma/Solitary Fibrous Tumor: A Case Report. Curr. Probl. Cancer Case Reports *6*, 100149. https://doi.org/10.1016/j.cpccr.2022.100149.
2. Sheehan, J., Kondziolka, D., Flickinger, J., Lunsford, L.D., Coffey, R.J., Loeffler, J.S., Sawaya, R., and Gutin, P.H. (2002). Radiosurgery for Treatment of Recurrent Intracranial Hemangiopericytomas. Neurosurgery *51*, 905–911. https://doi.org/10.1097/00006123-200210000-00008.
3. Ali, H.S.M., Endo, T., Endo, H., Murakami, K., and Tominaga, T. (2016). Intraspinal Dissemination of Intracranial Hemangiopericytoma: Case Report and Literature Review. In Proceedings of the Surgical Neurology International, *7* (Medknow Publications), pp. S1016–S1020.
4. Galanis, E., Buckner, J.C., Scheithauer, B.W., Kimmel, D.W., Schomberg, P.J., and Piepgras, D.G. (1998). Management of Recurrent Meningeal Hemangiopericytoma. Cancer *82*, 1915–1920. https://doi.org/10.1002/(SICI)1097-0142(19980515)82:10<1915::AID-CNCR15>3.0.CO;2-W.
5. Lee, S.J., Kim, S.T., Park, S.H., Choi, Y.L., Park, J.B., Kim, S.-J., and Lee, J. (2014). Successful Use of Pazopanib for Treatment of Refractory Metastatic Hemangiopericytoma. Clin. Sarcoma Res. *4*, 13. https://doi.org/10.1186/2045-3329-4-13.
6. Park, M.S., Ravi, V., Conley, A., Patel, S.R., Trent, J.C., Lev, D.C., Lazar, A.J., Wang, W.-L., Benjamin, R.S., and Araujo, D.M. (2013). The Role of Chemotherapy in Advanced Solitary Fibrous Tumors: A Retrospective Analysis. Clin. Sarcoma Res. *3*, 7. https://doi.org/10.1186/2045-3329-3-7.
7. Penel, N., Amela, E.Y., Decanter, G., Robin, Y.M., and Marec-Berard, P. (2012). Solitary Fibrous Tumors and So-Called Hemangiopericytoma. Sarcoma *2012*, 690251.
8. de Bernardi, A., Dufresne, A., Mishellany, F., Blay, J.-Y., Ray-Coquard, I., and Brahmi, M. (2022). Novel Therapeutic Options for Solitary Fibrous Tumor: Antiangiogenic Therapy and Beyond. Cancers *14*, 1064. https://doi.org/10.3390/cancers14041064.
9. Maruzzo, M., Martin-Liberal, J., Messiou, C., Miah, A., Thway, K., Alvarado, R., Judson, I., and Benson, C. (2015). Pazopanib as First Line Treatment for Solitary Fibrous Tumours: The Royal Marsden Hospital Experience. Clin. Sarcoma Res. *5*, 5. https://doi.org/10.1186/s13569-015-0022-2.
10. Park, M.S., Patel, S.R., Ludwig, J.A., Trent, J.C., Conrad, C.A., Lazar, A.J., Wang, W.L., Boonsirikamchai, P., Choi, H., Wang, X., et al. (2011). Activity of Temozolomide and Bevacizumab in the Treatment of Locally Advanced, Recurrent, and Metastatic Hemangiopericytoma and Malignant Solitary Fibrous Tumor. Cancer *117*, 4939–4947. https://doi.org/10.1002/cncr.26098.
11. Martin-Broto, J., Mondaza-Hernandez, J.L., Moura, D.S., and Hindi, N. (2021). A Comprehensive Review on Solitary Fibrous Tumor: New Insights for New Horizons. Cancers *13*, 2913. https://doi.org/10.3390/cancers13122913.
12. Robinson, D.R., Wu, Y.M., Kalyana-Sundaram, S., Cao, X., Lonigro, R.J., Sung, Y.S., Chen, C.L., Zhang, L., Wang, R., Su, F., et al. (2013). Identification of Recurrent NAB2-STAT6 Gene Fusions in Solitary Fibrous Tumor by Integrative Sequencing. Nat. Genet. *45*, 180–185. https://doi.org/10.1038/ng.2509.
13. Chmielecki, J., Crago, A.M., Rosenberg, M., O'Connor, R., Walker, S.R., Ambrogio, L., Auclair, D., McKenna, A., Heinrich, M.C., Frank, D.A., and Meyerson, M. (2013). Whole-Exome Sequencing Identifies a Recurrent NAB2-STAT6 Fusion in Solitary Fibrous Tumors. Nat. Genet. *45*, 131–132. https://doi.org/10.1038/ng.2522.
14. Guseva, N.V., Tanas, M.R., Stence, A.A., Sompallae, R., Schade, J.C., Bossler, A.D., Bellizzi, A.M., and Ma, D. (2016). The NAB2–STAT6 Gene Fusion in Solitary Fibrous Tumor Can Be Reliably Detected by Anchored Multiplexed PCR for Targeted next-Generation Sequencing. Cancer Genet. *209*, 303–312. https://doi.org/10.1016/j.cancergen.2016.05.071.
15. Tai, H.-C., Chuang, I.-C., Chen, T.-C., Li, C.-F., Huang, S.-C., Kao, Y.-C., Lin, P.-C., Tsai, J.-W., Lan, J., Yu, S.-C., et al. (2015). NAB2-STAT6 Fusion Types Account for Clinicopathological Variations in Solitary Fibrous Tumors. Mod. Pathol. *28*, 1324–1335. https://doi.org/10.1038/modpathol.2015.90.

16. Huang, S.-C., and Huang, H.-Y. (2019). Solitary Fibrous Tumor: An Evolving and Unifying Entity with Unsettled Issues. Histol. Histopathol. *34*, 313–334. https://doi.org/10.14670/HH-18-064.

17. Wang, B., Guo, H., Yu, H., Chen, Y., Xu, H., and Zhao, G. (2021). The Role of the Transcription Factor EGR1 in Cancer. Front. Oncol. *11*, 642547. https://doi.org/10.3389/fonc.2021.642547.

18. Zogg, H., Singh, R., and Ro, S. (2022). Current Advances in RNA Therapeutics for Human Diseases. Int. J. Mol. Sci. *23*, 2736. https://doi.org/10.3390/ijms23052736.

19. Shadid, M., Badawi, M., and Abulrob, A. (2021). Antisense Oligonucleotides: Absorption, Distribution, Metabolism, and Excretion. Expet Opin. Drug Metabol. Toxicol. *17*, 1281–1292. https://doi.org/10.1080/17425255.2021.1992382.

20. Gupta, A., Andresen, J.L., Manan, R.S., and Langer, R. (2021). Nucleic Acid Delivery for Therapeutic Applications. Adv. Drug Deliv. Rev. *178*, 113834. https://doi.org/10.1016/j.addr.2021.113834.

21. Saifullah, Motohashi, N., Tsukahara, T., and Aoki, Y. (2022). Development of Therapeutic RNA Manipulation for Muscular Dystrophy. Front. genome Ed. *4*, 863651. https://doi.org/10.3389/fgeed.2022.863651.

22. Sartorius, K., Antwi, S.O., Chuturgoon, A., Roberts, L.R., and Kramvis, A. (2022). RNA Therapeutic Options to Manage Aberrant Signaling Pathways in Hepatocellular Carcinoma: Dream or Reality? Front. Oncol. *12*, 891812. https://doi.org/10.3389/fonc.2022.891812.

23. Aimo, A., Castiglione, V., Rapezzi, C., Franzini, M., Panichella, G., Vergaro, G., Gillmore, J., Fontana, M., Passino, C., and Emdin, M. (2022). RNA-Targeting and Gene Editing Therapies for Transthyretin Amyloidosis. Nat. Rev. Cardiol. *19*, 655–667. https://doi.org/10.1038/s41569-022-00683-z.

24. Tarn, W.-Y., Cheng, Y., Ko, S.-H., and Huang, L.-M. (2021). Antisense Oligonucleotide-Based Therapy of Viral Infections. Pharmaceutics *13*, 2015. https://doi.org/10.3390/pharmaceutics13122015.

25. Grabowska-Pyrzewicz, W., Want, A., Leszek, J., and Wojda, U. (2021). Antisense Oligonucleotides for Alzheimer's Disease Therapy: From the MRNA to MiRNA Paradigm. EBioMedicine *74*, 103691. https://doi.org/10.1016/j.ebiom.2021.103691.

26. Edinoff, A.N., Nguyen, L.H., Odisho, A.S., Maxey, B.S., Pruitt, J.W., Girma, B., Cornett, E.M., Kaye, A.M., and Kaye, A.D. (2021). The Antisense Oligonucleotide Nusinersen for Treatment of Spinal Muscular Atrophy. Orthop. Rev. *13*, 24934. https://doi.org/10.52965/001c.24934.

27. Wiggins, R., and Feigin, A. (2021). Emerging Therapeutics in Huntington's Disease. Expet Opin. Emerg. Drugs *26*, 295–302. https://doi.org/10.1080/14728214.2021.1962285.

28. Amado, D.A., and Davidson, B.L. (2021). Gene Therapy for ALS: A Review. Mol. Ther. *29*, 3345–3358. https://doi.org/10.1016/j.ymthe.2021.04.008.

29. Robson, F., Khan, K.S., Le, T.K., Paris, C., Demirbag, S., Barfuss, P., Rocchi, P., and Ng, W.-L. (2020). Coronavirus RNA Proofreading: Molecular Basis and Therapeutic Targeting. Mol. Cell *79*, 710–727. https://doi.org/10.1016/j.molcel.2020.07.027.

30. Li, Y., Nguyen, J.T., Ammanamanchi, M., Zhou, Z., Harbut, E.F., Mondaza-Hernandez, J.L., Meyer, C.A., Moura, D.S., Martin-Broto, J., Hayenga, H.N., et al. (2023). Reduction of Tumor Growth with RNA-Targeting Treatment of the NAB2-STAT6 Fusion Transcript in Solitary Fibrous Tumor Models. Cancers *15*, 3127.

31. Li, Y., Mendiratta, S., Ehrhardt, K., Kashyap, N., White, M.A., and Bleris, L. (2016). Exploiting the CRISPR/Cas9 PAM Constraint for Single-Nucleotide Resolution Interventions. PLoS One *11*, e0144970. https://doi.org/10.1371/journal.pone.0144970.

32. Li, Y., Nowak, C.M., Withers, D., Pertsemlidis, A., and Bleris, L. (2018). CRISPR-Based Editing Reveals Edge-Specific Effects in Biological Networks. Crisol J. *1*, 286–293.

33. Nowak, C.M., Lawson, S., Zerez, M., and Bleris, L. (2016). Guide RNA Engineering for Versatile Cas9 Functionality. Nucleic Acids Res. *44*, 9555–9564.

34. Moore, R., Spinhirne, A., Lai, M.J., Preisser, S., Li, Y., Kang, T., and Bleris, L. (2015). CRISPR-Based Self-Cleaving Mechanism for Controllable Gene Delivery in Human Cells. Nucleic Acids Res. *43*, 1297–1303. https://doi.org/10.1093/nar/gku1326.

35. Quarton, T., Kang, T., Papakis, V., Nguyen, K., Nowak, C., Li, Y., and Bleris, L. (2020). Uncoupling Gene Expression Noise along the Central Dogma Using Genome Engineered Human Cell Lines. Nucleic Acids Res. *48*, 9406–9413. https://doi.org/10.1093/nar/gkaa668.

36. Hsieh, M.-H., Choe, J.H., Gadhvi, J., Kim, Y.J., Arguez, M.A., Palmer, M., Gerold, H., Nowak, C., Do, H., Mazambani, S., et al. (2019). P63 and SOX2 Dictate Glucose Reliance and Metabolic Vulnerabilities in Squamous Cell Carcinomas. Cell Rep. *28*, 1860–1878.e9. https://doi.org/10.1016/J.CELREP.2019.07.027.

37. Binnemars-Postma, K., Bansal, R., Storm, G., and Prakash, J. (2018). Targeting the Stat6 Pathway in Tumor-Associated Macrophages Reduces Tumor Growth and Metastatic Niche Formation in Breast Cancer. FASEB J. Off. Publ. Fed. Am. Soc. Exp. Biol. *32*, 969–978. https://doi.org/10.1096/fj.201700629R.

38. Kamerkar, S., Leng, C., Burenkova, O., Jang, S.C., McCoy, C., Zhang, K., Dooley, K., Kasera, S., Zi, T., Sisó, S., et al. (2022). Exosome-Mediated Genetic Reprogramming of Tumor-Associated Macrophages by ExoASO-STAT6 Leads to Potent Monotherapy Antitumor Activity. Sci. Adv. *8*, eabj7002. https://doi.org/10.1126/sciadv.abj7002.

39. Lesterhuis, W.J., Punt, C.J.A., Hato, S.V., Eleveld-Trancikova, D., Jansen, B.J.H., Nierkens, S., Schreibelt, G., de Boer, A., Van Herpen, C.M.L., Kaanders, J.H., et al. (2011). Platinum-Based Drugs Disrupt STAT6-Mediated Suppression of Immune Responses against Cancer in Humans and Mice. J. Clin. Invest. *121*, 3100–3108. https://doi.org/10.1172/JCI43656.

40. Haselager, M.V., Thijssen, R., Bax, D., Both, D., De Boer, F., Mackay, S., Dubois, J., Mellink, C., Kater, A.P., and Eldering, E. (2022). JAK-STAT Signaling Shapes the NF-KB Response in CLL towards Venetoclax Sensitivity or Resistance via Bcl-XL. Mol. Oncol. *17*, 1112–1128. https://doi.org/10.1002/1878-0261.13364.

41. Hong, D., Kurzrock, R., Kim, Y., Woessner, R., Younes, A., Nemunaitis, J., Fowler, N., Zhou, T., Schmidt, J., Jo, M., et al. (2015). AZD9150, a next-Generation Antisense Oligonucleotide Inhibitor of STAT3 with Early Evidence of Clinical Activity in Lymphoma and Lung Cancer. Sci. Transl. Med. *7*, 314ra185. https://doi.org/10.1126/scitranslmed.aac5272.

42. Zhang, H., Löwenberg, E.C., Crosby, J.R., MacLeod, A.R., Zhao, C., Gao, D., Black, C., Revenko, A.S., Meijers, J.C.M., Stroes, E.S., et al. (2010). Inhibition of the Intrinsic Coagulation Pathway Factor XI by Antisense Oligonucleotides: A Novel Antithrombotic Strategy with Lowered Bleeding Risk. Blood *116*, 4684–4692. https://doi.org/10.1182/blood-2010-04-277798.

43. Gutschner, T., Hämmerle, M., Eissmann, M., Hsu, J., Kim, Y., Hung, G., Revenko, A., Arun, G., Stentrup, M., Gross, M., et al. (2013). The Noncoding RNA MALAT1 Is a Critical Regulator of the Metastasis Phenotype of Lung Cancer Cells. Cancer Res. *73*, 1180–1189. https://doi.org/10.1158/0008-5472.CAN-12-2850.

44. Ross, S.J., Revenko, A.S., Hanson, L.L., Ellston, R., Staniszewska, A., Whalley, N., Pandey, S.K., Revill, M., Rooney, C., Buckett, L.K., et al. (2017). Targeting KRAS-Dependent Tumors with AZD4785, a High-Affinity Therapeutic Antisense Oligonucleotide Inhibitor of KRAS. Sci. Transl. Med. *9*, eaal5253. https://doi.org/10.1126/scitranslmed.aal5253.

45. Reilley, M.J., McCoon, P., Cook, C., Lyne, P., Kurzrock, R., Kim, Y., Woessner, R., Younes, A., Nemunaitis, J., Fowler, N., et al. (2018). STAT3 Antisense Oligonucleotide AZD9150 in a Subset of Patients with Heavily Pretreated Lymphoma: Results of a Phase 1b Trial. J. Immunother. cancer *6*, 119. https://doi.org/10.1186/s40425-018-0436-5.

46. Ferrone, J.D., Bhattacharjee, G., Revenko, A.S., Zanardi, T.A., Warren, M.S., Derosier, F.J., Viney, N.J., Pham, N.C., Kaeser, G.E., Baker, B.F., et al. (2019). IONIS-PKK(Rx) a Novel Antisense Inhibitor of Prekallikrein and Bradykinin Production. Nucleic Acid Therapeut. *29*, 82–91. https://doi.org/10.1089/nat.2018.0754.

47. Barrett, T.J., Wu, B.G., Revenko, A.S., MacLeod, A.R., Segal, L.N., and Berger, J.S. (2020). Antisense Oligonucleotide Targeting of Thrombopoietin Represents a Novel Platelet Depletion Method to Assess the Immunomodulatory Role of Platelets. J. Thromb. Haemostasis *18*, 1773–1782. https://doi.org/10.1111/jth.14808.

48. Sacco, A., Federico, C., Todoerti, K., Ziccheddu, B., Palermo, V., Giacomini, A., Ravelli, C., Maccarinelli, F., Bianchi, G., Belotti, A., et al. (2021). Specific Targeting of the KRAS Mutational Landscape in Myeloma as a Tool to Unveil the Elicited Antitumor Activity. Blood *138*, 1705–1720. https://doi.org/10.1182/blood.2020010572.

49. Revenko, A., Carnevalli, L.S., Sinclair, C., Johnson, B., Peter, A., Taylor, M., Hettrick, L., Chapman, M., Klein, S., Solanki, A., et al. (2022). Direct Targeting of FOXP3 in

Tregs with AZD8701, a Novel Antisense Oligonucleotide to Relieve Immunosuppression in Cancer. J. Immunother. cancer *10*, e003892. https://doi.org/10.1136/jitc-2021-003892.

50. Mondaza-Hernandez, J.L., Moura, D.S., Lopez-Alvarez, M., Sanchez-Bustos, P., Blanco-Alcaina, E., Castilla-Ramirez, C., Collini, P., Merino-Garcia, J., Zamora, J., Carrillo-Garcia, J., et al. (2022). ISG15 as a Prognostic Biomarker in Solitary Fibrous Tumour. Cell. Mol. Life Sci. *79*, 434. https://doi.org/10.1007/s00018-022-04454-4.

51. Sbaraglia, M., Bellan, E., and Dei Tos, A.P. (2021). The 2020 WHO Classification of Soft Tissue Tumours: News and Perspectives. Pathologica *113*, 70–84. https://doi.org/10.32074/1591-951X-213.

52. van de Rijn, M., Lombard, C.M., and Rouse, R.V. (1994). Expression of CD34 by Solitary Fibrous Tumors of the Pleura, Mediastinum, and Lung. Am. J. Surg. Pathol. *18*, 814–820. https://doi.org/10.1097/00000478-199408000-00008.

53. Prakash, T.P., Yu, J., Shen, W., De Hoyos, C.L., Berdeja, A., Gaus, H., Liang, X.-H., Crooke, S.T., and Seth, P.P. (2021). Site-Specific Incorporation of 2',5'-Linked Nucleic Acids Enhances Therapeutic Profile of Antisense Oligonucleotides. ACS Med. Chem. Lett. *12*, 922–927. https://doi.org/10.1021/acsmedchemlett.1c00072.

54. Shen, W., De Hoyos, C.L., Migawa, M.T., Vickers, T.A., Sun, H., Low, A., Bell, T.A., 3rd, Rahdar, M., Mukhopadhyay, S., Hart, C.E., et al. (2019). Chemical Modification of PS-ASO Therapeutics Reduces Cellular Protein-Binding and Improves the Therapeutic Index. Nat. Biotechnol. *37*, 640–650. https://doi.org/10.1038/s41587-019-0106-2.

55. Kamola, P.J., Maratou, K., Wilson, P.A., Rush, K., Mullaney, T., McKevitt, T., Evans, P., Ridings, J., Chowdhury, P., Roulois, A., et al. (2017). Strategies for In Vivo Screening and Mitigation of Hepatotoxicity Associated with Antisense Drugs. Mol. Ther. Nucleic Acids *8*, 383–394. https://doi.org/10.1016/j.omtn.2017.07.003.

56. Berrouague, S., Rouag, M., Khaldi, T., Boumendjel, A., Boumendjel, M., Taibi, F., and Messarah, M. (2019). Efficacy of Allium Sativum Oil to Alleviate Tebuconazol-Induced Oxidative Stress in the Liver of Adult Rats. Cell. Mol. Biol. (Noisy-Le-Grand) *65*, 23–31.

57. Ämmälä, C., Drury, W.J., 3rd, Knerr, L., Ahlstedt, I., Stillemark-Billton, P., Wennberg-Huldt, C., Andersson, E.-M., Valeur, E., Jansson-Löfmark, R., Janzén, D., et al. (2018). Targeted Delivery of Antisense Oligonucleotides to Pancreatic β-Cells. Sci. Adv. *4*, eaat3386. https://doi.org/10.1126/sciadv.aat3386.

58. Moura, D.S., Díaz-Martín, J., Bagué, S., Orellana-Fernandez, R., Sebio, A., Mondaza-Hernandez, J.L., Salguero-Aranda, C., Rojo, F., Hindi, N., Fletcher, C.D.M., and Martin-Broto, J. (2021). A Novel NFIX-STAT6 Gene Fusion in Solitary Fibrous Tumor: A Case Report. Int. J. Mol. Sci. *22*, 7514. https://doi.org/10.3390/ijms22147514.

59. He, K., Barsoumian, H.B., Puebla-Osorio, N., Hu, Y., Sezen, D., Wasley, M.D., Bertolet, G., Zhang, J., Leuschner, C., Yang, L., et al. (2023). Inhibition of STAT6 with Antisense Oligonucleotides Enhances the Systemic Antitumor Effects of Radiotherapy and Anti-PD-1 in Metastatic Non-Small Cell Lung Cancer. Cancer Immunol. Res. *11*, 486–500. https://doi.org/10.1158/2326-6066.CIR-22-0547.

60. Gagliardi, M., and Ashizawa, A.T. (2021). The Challenges and Strategies of Antisense Oligonucleotide Drug Delivery. Biomedicines *9*, 433. https://doi.org/10.3390/biomedicines9040433.

61. Erba, H.P., Sayar, H., Juckett, M., Lahn, M., Andre, V., Callies, S., Schmidt, S., Kadam, S., Brandt, J.T., Van Bockstaele, D., and Andreeff, M. (2013). Safety and Pharmacokinetics of the Antisense Oligonucleotide (ASO) LY2181308 as a Single-Agent or in Combination with Idarubicin and Cytarabine in Patients with Refractory or Relapsed Acute Myeloid Leukemia (AML). Invest. N. Drugs *31*, 1023–1034. https://doi.org/10.1007/s10637-013-9935-x.

62. Du, W., Huang, H., Sorrelle, N., and Brekken, R.A. (2018). Sitravatinib Potentiates Immune Checkpoint Blockade in Refractory Cancer Models. JCI insight *3*, e124184. https://doi.org/10.1172/jci.insight.124184.

63. Ingham, M., Lee, S., Van Tine, B.A., Choy, E., Oza, J., Doshi, S., Ge, L., Oppelt, P., Cote, G., Corgiat, B., et al. (2023). A Single-Arm Phase II Trial of Sitravatinib in Advanced Well-Differentiated/Dedifferentiated Liposarcoma. Clin. Cancer Res. *29*, 1031–1039. https://doi.org/10.1158/1078-0432.CCR-22-3351.

64. Martin-Broto, J., Stacchiotti, S., Lopez-Pousa, A., Redondo, A., Bernabeu, D., de Alava, E., Casali, P.G., Italiano, A., Gutierrez, A., Moura, D.S., et al. (2019). Pazopanib for Treatment of Advanced Malignant and Dedifferentiated Solitary Fibrous Tumour: A Multicentre, Single-Arm, Phase 2 Trial. Lancet Oncol. *20*, 134–144. https://doi.org/10.1016/S1470-2045(18)30676-4.

65. Martin-Broto, J., Cruz, J., Penel, N., Le Cesne, A., Hindi, N., Luna, P., Moura, D.S., Bernabeu, D., de Alava, E., Lopez-Guerrero, J.A., et al. (2020). Pazopanib for Treatment of Typical Solitary Fibrous Tumours: A Multicentre, Single-Arm, Phase 2 Trial. Lancet Oncol. *21*, 456–466. https://doi.org/10.1016/S1470-2045(19)30826-5.

OMTN, Volume *35*

Supplemental information

STAT6-targeting antisense oligonucleotides against solitary fibrous tumor

Yi Li, Jose L. Mondaza-Hernandez, David S. Moura, Alexey S. Revenko, Angelica Tolentino, John T. Nguyen, Nam Tran, Clark A. Meyer, Jose Merino-Garcia, Rafael Ramos, Davide Di Lernia, Javier Martin-Broto, Heather N. Hayenga, and Leonidas Bleris

Supplemental materials

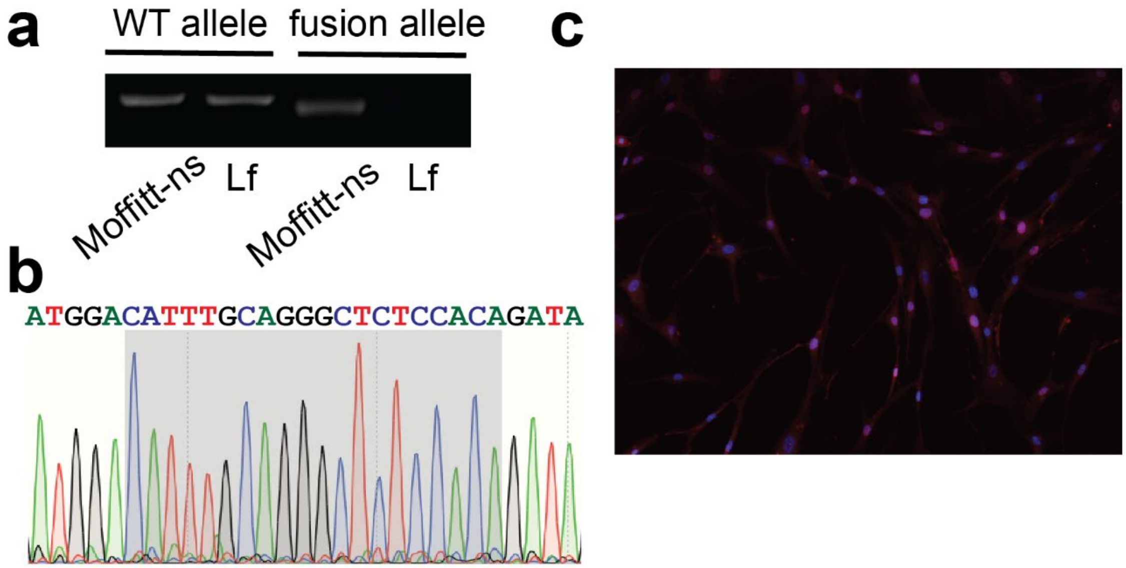

Figure S1. Primary SFT cell line established at Moffitt Cancer Center (Moffitt-ns). (a) RNA RT-PCR assays confirmed that Moffitt-ns cells are heterozygous. **(b)** Sanger confirmed the fusion as NAB2exon5-STAT6exon16. **(c)** ICC showed prominent nuclear expression of the fusion protein. (red) anti-STAT6 antibody conjugated with Alexa Fluor 594, (blue) DAPI.

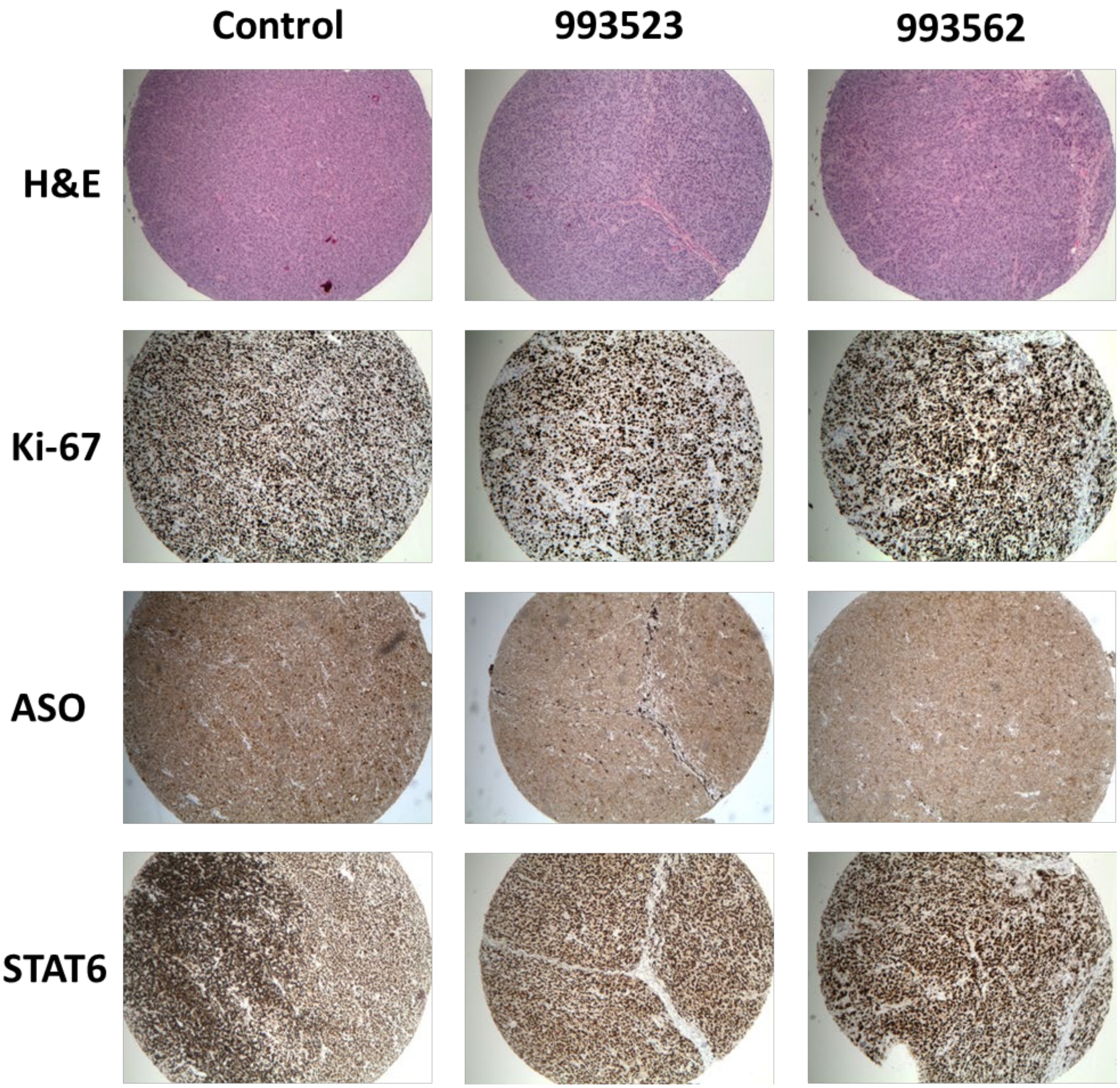

Figure S2. Representative images for H&E and IHC (immunohistochemistry) staining using IEC139 PDX tissues. The percentage of Ki-67-positive cells were significantly lower in 993523-treated tumor tissues compared to control-treated sample. In contrast, no significant differences were observed between different treatment groups for ASO or total STAT6 (NAB2-STAT6 fusion and wild type STAT6).

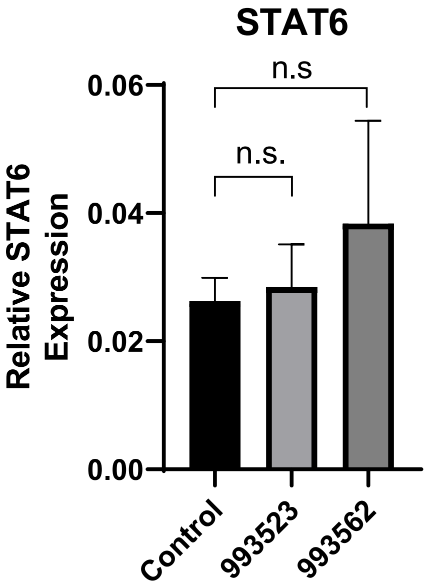

Figure S3. ASOs 993523 and 993562 did not significantly downregulate the expression of wild type STAT6 transcript within the tumor tissues at the end of treatment. For statistical analysis, two-tailed t-tests were conducted.

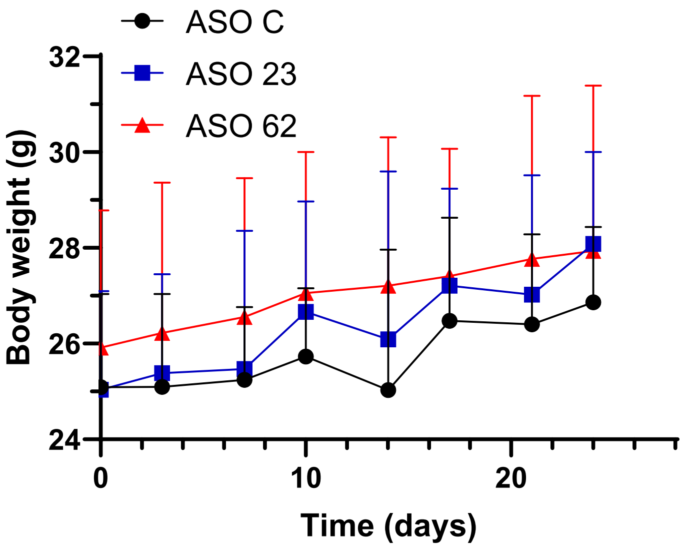

Figure S4. No significant changes in body weight were observed during the entire treatment period among the three treatment groups (n=8).

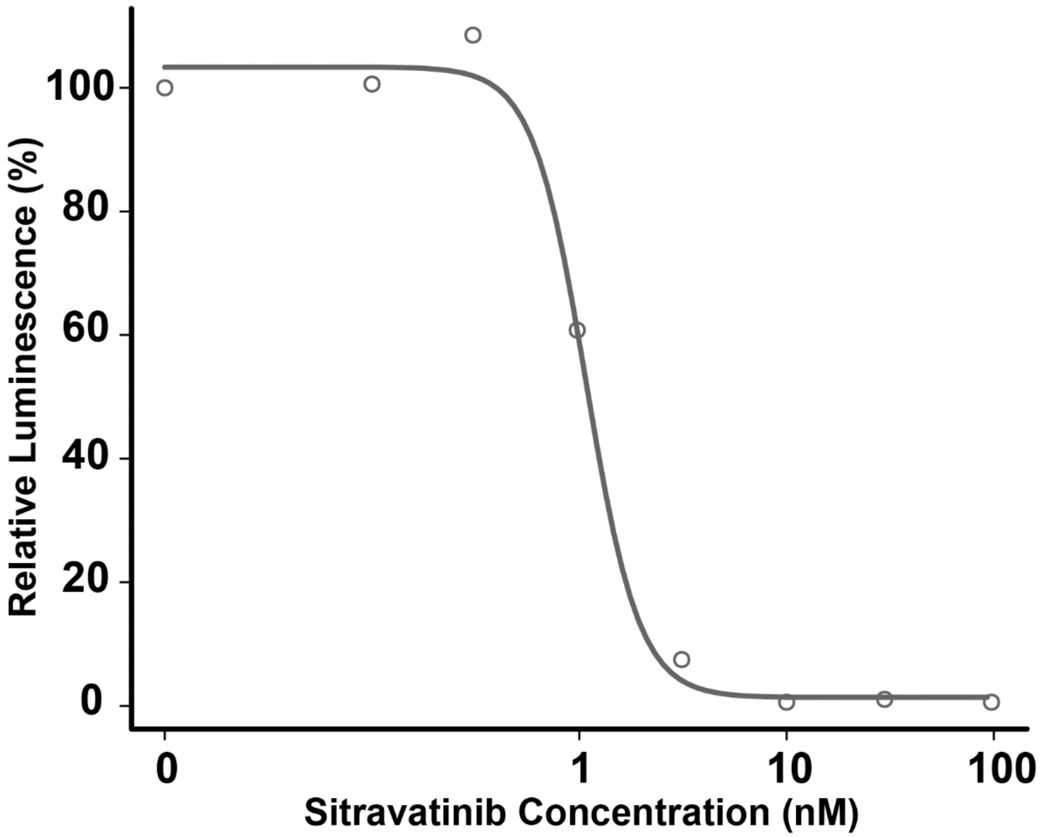

Figure S5. Tyrosine kinase inhibitor Sitravatinib potently inhibits SFT cell proliferation *in vitro* (IC50: 1.1 nM).

Table S1. Primers used in this study.

Primer	Sequence (5'->3')
P1	GGTCATGTCCAAGGCTGACGCCGCCCCCTG
P2	GTAGCTGGGACATAACCCCTGCCATCCTTACC
P3	GGTCATGTCCAAGGCTGACGCCGCCCCCTG
P4	CCTCCCCTCCCTGGCTGTGCGTAGCTCTGT
P5	CCTGTCTGGGGAGAGTCTGGATG
P6	GGGGGGATGGAGTGAGAGTGTG
P7	GGGGGGATGGAGTGAGAGTGTG
P8	CTTTCCGGAGCCACTACAAG
P9	AGGAAGTGGTTGGTCCCTTT
P10	CTGCAGCAGACACTGATGGA
P11	TACCCATCTGTTCAGCTGCG
P12	GAATCCGGGATCTTGCTCAG
P13	CCTGCCATCCTTACCCATCT
P14	CGAAGCCACCTCTCGCAG
P15	CTTGTAGTGGCTCCGGAAAG
P16	AACCTTTCTCCTCCGCTTCA
P17	TGGCTGGATGTTCTCTATCTGT
P18	AGCAGACACTGATGGACGAG
P19	TGGGCTTCTTGGGATAGAGA
P20	AATCCCATCACCATCTTCCA
P21	TGGACTCCACGACGTACTCA

Table S2. Candidate STAT6 3’-UTR-targeting ASOs used in this study.

ID	Sequence (5'->3')	Length	Molecular Weight	Extinction Coefficient
993523	GTAGAAGAGCTGTCTC	16	5456.5166	161.18
993544	CCAATATCCTCTATCC	16	5327.5137	146.08
993558	ACATATCCTAGGTACA	16	5398.2	166.38
993562	CAACTAAGGTGCCAGC	16	5438.5479	157.62
993563	GCAACTAAGGTGCCAG	16	5464.5449	162.18
993568	TTCTATGTGGTCATGC	16	5413.4771	149.32
993573	CAGATAGACACATGTT	16	5424.5176	166.02
993578	AGCATATGTCAGAGAG	16	5475.5278	173.8

Table S3. Lf-ns NAB2exon6-STAT6exon17 mRNA transcript sequence.

AGACAGAGGCGCGGAGGCTCGGAGAGAGAAGACGTGGAGGGAGGGACAGAGCCTG
GACAGCGGTGGACACGGCATCGTGCGCGGGGAAGAGGGCAGCACGCAGCAGGCGC
CGAGCGCCGGGCACCGAGAAGGGCAGCCCGGGTGATCTCCGGCCGTCCATGCACAG
AGCGCCTTCCCCCACAGCCGAGCAGCCGCCGGGCGGAGGGGACAGCGCCCGCCGGA
CCCTGCAGCCCAGACTCAAGCCCAGTGCCCGAGCCATGGCACTGCCTCGGACGCTG
GGGGAGCTGCAGCTGTACCGGGTCCTGCAGCGCGCCAACCTCCTTTCCTACTATGAG
ACCTTCATCCAGCAGGGAGGGGACGACGTGCAGCAGCTGTGTGAGGCGGGTGAGGA
GGAGTTTCTGGAGATCATGGCACTTGTGGGCATGGCCACCAAGCCCCTCCATGTCCG
GCGCCTGCAGAAGGCACTGAGAGAGTGGGCCACCAATCCAGGGCTCTTCAGTCAAC
CAGTGCCTGCTGTTCCCGTCTCCAGCATCCCGCTCTTCAAGATCTCTGAGACTGCGG
GTACCCGGAAAGGGAGCATGAGCAATGGGCATGGCAGCCCAGGGGAAAAGGCAGG
CAGTGCCCGCAGTTTTAGCCCCAAGAGCCCCCTTGAACTTGGAGAGAAGCTATCACC
ACTGCCTGGGGGACCTGGGGCAGGGGACCCCCGGATCTGGCCAGGCCGGAGCACTC
CAGAGTCGGACGTTGGGGCAGGAGGAGAAGAGGAGGCTGGCTCGCCCCCCTTCTCC
CCCCCTGCAGGGGGAGGAGTCCCTGAGGGGACTGGGGCTGGGGGGCTGGCAGCAG
GTGGGACTGGGGGTGGTCCAGACCGACTGGAGCCAGAGATGGTACGCATGGTGGTG
GAAAGTGTGGAGAGGATCTTCCGGAGCTTCCCAAGGGGGGATGCTGGGGAGGTCAC
ATCCCTGCTAAAGCTGAATAAGAAGCTGGCACGGAGCGTTGGGCACATCTTTGAGA
TGGATGATAATGACAGCCAGAAGGAAGAGGAGATCCGCAAATACAGCATCATCTAT
GGCCGTTTCGACTCTAAGCGGCGGGAGGGCAAGCAGCTCAGCCTGCACGAGCTCAC
CATCAACGAGGCTGCTGCCCAGTTCTGCATGAGGGACAACACGCTCTTATTACGGAG
AGTGGAGCTCTTCTCTTTGTCCCGCCAAGTAGCCCGAGAGAGCACCTACTTGTCCTC
CTTGAAGGGCTCCAGGCTTCACCCTGAAGAACTGGGAGGCCCTCCACTGAAGAAGC
TGAAACAAGAGGTTGGAGAACAGAGTCACCCTGAAATCCAGCAGCCTCCCCCAGGC
CCTGAGTCCTATGTACCCCCATACCGCCCCAGCCTGGAGGAGGACAGCGCCAGCCT
GTCTGGGGAGAGTCTGGATGGACATTTGCAGGCTGTGGGGTCATGTCCAAGGCTGA
CGCCGCCCCCTGCTGACCTGCCTCTGGCATTGCCAGCCCATGGGCTATGGAGCCGAC
ACATCCTGCAGCAGACACTGATGGACGAGGGGCTGCGGCTCGCCCGCCTCGTCTCCC
ACGACCGCGTGGCCGCCTCAGCCCCTGTGTGCCTGCGAAGCCACCTCTCGCAGCTG
AACAGATGGGTAAGGATGGCAGGGGTTATGTCCCAGCTACCATCAAGATGACCGTG
GAAAGGGACCAACCACTTCCTACCCCAGAGCTCCAGATGCCTACCATGGTGCCTTCT
TATGACCTTGGAATGGCCCCTGATTCCTCCATGAGCATGCAGCTTGGCCCAGATATG
GTGCCCCAGGTGTACCCACCACACTCTCACTCCATCCCCCCGTATCAAGGCCTCTCC
CCAGAAGAATCAGTCAACGTGTTGTCAGCCTTCCAGGAGCCTCACCTGCAGATGCCC
CCCAGCCTGGGCCAGATGAGCCTGCCCTTTGACCAGCCTCACCCCCAGGGCCTGCTG
CCGTGCCAGCCTCAGGAGCATGCTGTGTCCAGCCCTGACCCCCTGCTCTGCTCAGAT
GTGACCATGGTGGAAGACAGCTGCCTGAGCCAGCCAGTGACAGCGTTTCCTCAGGG
CACTTGGATTGGTGAAGACATATTCCCTCCTCTGCTGCCTCCCACTGAACAGGACCT
CACTAAGCTTCTCCTGGAGGGGCAAGGGGAGTCGGGGGGAGGGTCCTTGGGGGCAC
AGCCCCTCCTGCAGCCCTCCCACTATGGGCAATCTGGGATCTCAATGTCCCACATGG
ACCTAAGGGCCAACCCCAGTTGGTGATCCCAGCTGGAGGGAGAACCCAAAGAGACA
GCTCTTCTACTACCCCCACAGACCTGCTCTGGACACTTGCTCATGCCCTGCCAAGCA
GCAGATGGGGAGGGTGCCCTCCTATCCCCACCTACTCCTGGGTCAGGAGGAAAAGA
CTAACAGGAGAATGCACAGTGGGTGGAGCCAATCCACTCCTTCCTTTCTATCATTCC

CCTGCCCACCTCCTTCCAGCACTGACTGGAAGGGAAGTTCAGGCTCTGAGACACACC
CCAACATGCCTGCACCTGCAGCGCACACGCACGCACACACACATACAGAGCTCT
CTGAGGGTGATGGGGCTGAGCAGGAGGGGGGCTGGGTAAGAGCACAGGTTAGGGC
ATGGAAGGCTTCTCCGCCCATTCTGACCCAGGGCCTAGGACGGATAGGCAGGAACA
TACAGACACATTTACACTAGAGGCCAGGGATAGAGGATATTGGGTCTCAGCCCTAG
GGGAATGGGAAGCAGCTCAAGGGACCCTGGGTGGGAGCATAGGAGGGGTCTGGAC
ATGTGGTTACTAGTACAGGTTTTGCCCTGATTAAAAAATCTCCCAAAGCCCCAAATT
CCTGTTAGCCAGGTGGAGGCTTCTGATACGTGTATGAGACTATGCAAAAGTACAAG
GGCTGAGATTCTTCGTGTATAGCTGTGTGAACGTGTATGTACCTAGGATATGTTAAA
TGTATAGCTGGCACCTTAGTTGCATGACCACATAGAACATGTGTCTATCTGCTTTTGC
CTACGTGACAACACAAATTTGGGAGGGTGAGACACTGCACAGAAGACAGCAGCAAG
TGTGCTGGCCTCTCTGACATATGCTAACCCCCAAATACTCTGAATTTGGAGTCTGACT
GTGCCCAAGTGGGTCCAAGTGGCTGTGACATCTACGTATGGCTCCACACCTCCAATG
CTGCCTGGGAGCCAGGGTGAGAGTCTGGGTCCAGGCCTGGCCATGTGGCCCTCCAG
TGTATGAGAGGGCCCTGCCTGCTGCATCTTTTCTGTTGCCCCATCCACCGCCAGCTTC
CCTTCACTCCCCTATCCCATTCTCCCTCTCAAGGCAGGGGTCATAGATCCTAAGCCAT
AAAATAAATTTTATTCCAAAATAACAAAATAAATAATCTACTGTACACAATCTGAAA
A

Blue: NAB2 exons 1-6
Green: STAT6 exons 17-22
Red: ASO IONIS993523 target site

Table S4. Moffitt-ns NAB2exon5-STAT6exon16 mRNA transcript sequence:

AGACAGAGGCGCGGAGGCTCGGAGAGAGAAGACGTGGAGGGAGGGACAGAGCCTG
GACAGCGGTGGACACGGCATCGTGCGCGGGGAAGAGGGCAGCACGCAGCAGGCGC
CGAGCGCCGGGCACCGAGAAGGGCAGCCCGGGTGATCTCCGGCCGTCCATGCACAG
AGCGCCTTCCCCCACAGCCGAGCAGCCGCCGGGCGGAGGGGACAGCGCCCGCCGGA
CCCTGCAGCCCAGACTCAAGCCCAGTGCCCGAGCCATGGCACTGCCTCGGACGCTG
GGGGAGCTGCAGCTGTACCGGGTCCTGCAGCGCGCCAACCTCCTTTCCTACTATGAG
ACCTTCATCCAGCAGGGAGGGGACGACGTGCAGCAGCTGTGTGAGGCGGGTGAGGA
GGAGTTTCTGGAGATCATGGCACTTGTGGGCATGGCCACCAAGCCCCTCCATGTCCG
GCGCCTGCAGAAGGCACTGAGAGAGTGGGCCACCAATCCAGGGCTCTTCAGTCAAC
CAGTGCCTGCTGTTCCCGTCTCCAGCATCCCGCTCTTCAAGATCTCTGAGACTGCGG
GTACCCGGAAAGGGAGCATGAGCAATGGGCATGGCAGCCCAGGGGAAAAGGCAGG
CAGTGCCCGCAGTTTTAGCCCCAAGAGCCCCCTTGAACTTGGAGAGAAGCTATCACC
ACTGCCTGGGGGACCTGGGGCAGGGGACCCCCGGATCTGGCCAGGCCGGAGCACTC
CAGAGTCGGACGTTGGGGCAGGAGGAGAAGAGGAGGCTGGCTCGCCCCCCTTCTCC
CCCCCTGCAGGGGGAGGAGTCCCTGAGGGGACTGGGGCTGGGGGGCTGGCAGCAG
GTGGGACTGGGGGTGGTCCAGACCGACTGGAGCCAGAGATGGTACGCATGGTGGTG
GAAAGTGTGGAGAGGATCTTCCGGAGCTTCCCAAGGGGGGATGCTGGGGAGGTCAC
ATCCCTGCTAAAGCTGAATAAGAAGCTGGCACGGAGCGTTGGGCACATCTTTGAGA
TGGATGATAATGACAGCCAGAAGGAAGAGGAGATCCGCAAATACAGCATCATCTAT
GGCCGTTTCGACTCTAAGCGGCGGGAGGGCAAGCAGCTCAGCCTGCACGAGCTCAC
CATCAACGAGGCTGCTGCCCAGTTCTGCATGAGGGACAACACGCTCTTATTACGGAG
AGTGGAGCTCTTCTCTTTGTCCCGCCAAGTAGCCCGAGAGAGCACCTACTTGTCCTC
CTTGAAGGGCTCCAGGCTTCACCCTGAAGAACTGGGAGGCCCTCCACTGAAGAAGC
TGAAACAAGAGGTTGGAGAACAGAGTCACCCTGAAATCCAGCAGCCTCCCCCAGGC
CCTGAGTCCTATGTACCCCCATACCGCCCCAGCCTGGAGGAGGACAGCGCCAGCCT
GTCTGGGGAGAGTCTGGATGGACATTTGCAGGGCTCTCCACAGATAGAGAACATCC
AGCCATTCTCTGCCAAAGACCTGTCCATTCGCTCACTGGGGGACCGAATCCGGGATC
TTGCTCAGCTCAAAAATCTCTATCCCAAGAAGCCCAAGGATGAGGCTTTCCGGAGCC
ACTACAAGCCTGAACAGATGGGTAAGGATGGCAGGGGTTATGTCCCAGCTACCATC
AAGATGACCGTGGAAAGGGACCAACCACTTCCTACCCCAGAGCTCCAGATGCCTAC
CATGGTGCCTTCTTATGACCTTGGAATGGCCCCTGATTCCTCCATGAGCATGCAGCTT
GGCCCAGATATGGTGCCCCAGGTGTACCCACCACACTCTCACTCCATCCCCCCGTAT
CAAGGCCTCTCCCCAGAAGAATCAGTCAACGTGTTGTCAGCCTTCCAGGAGCCTCAC
CTGCAGATGCCCCCCAGCCTGGGCCAGATGAGCCTGCCCTTTGACCAGCCTCACCCC
CAGGGCCTGCTGCCGTGCCAGCCTCAGGAGCATGCTGTGTCCAGCCCTGACCCCCTG
CTCTGCTCAGATGTGACCATGGTGGAAGACAGCTGCCTGAGCCAGCCAGTGACAGC
GTTTCCTCAGGGCACTTGGATTGGTGAAGACATATTCCCTCCTCTGCTGCCTCCCACT
GAACAGGACCTCACTAAGCTTCTCCTGGAGGGGCAAGGGGAGTCGGGGGGAGGGTC
CTTGGGGGCACAGCCCCTCCTGCAGCCCTCCCACTATGGGCAATCTGGGATCTCAAT
GTCCCACATGGACCTAAGGGCCAACCCCAGTTGGTGATCCCAGCTGGAGGGAGAAC
CCAAAGAGACAGCTCTTCTACTACCCCCACAGACCTGCTCTGGACACTTGCTCATGC
CCTGCCAAGCAGCAGATGGGGAGGGTGCCCTCCTATCCCCACCTACTCCTGGGTCAG
GAGGAAAAGACTAACAGGAGAATGCACAGTGGGTGGAGCCAATCCACTCCTTCCTT
TCTATCATTCCCCTGCCCACCTCCTTCCAGCACTGACTGGAAGGGAAGTTCAGGCTC

TGAGACACACCCCAACATGCCTGCACCTGCAGCGCACACGCACGCACACACACA
TACAGAGCTCTCTGAGGGTGATGGGGCTGAGCAGGAGGGGGGCTGGGTAAGAGCAC
AGGTTAGGGCATGGAAGGCTTCTCCGCCCATTCTGACCCAGGGCCTAGGACGGATA
GGCAGGAACATACAGACACATTTACACTAGAGGCCAGGGATAGAGGATATTGGGTC
TCAGCCCTAGGGGAATGGGAAGCAGCTCAAGGGACCCTGGGTGGGAGCATAGGAG
GGGTCTGGACATGTGGTTACTAGTACAGGTTTTGCCCTGATTAAAAAATCTCCCAAA
GCCCCAAATTCCTGTTAGCCAGGTGGAGGCTTCTGATACGTGTATGAGACTATGCAA
AAGTACAAGGGCTGAGATTCTTCGTGTATAGCTGTGTGAACGTGTATGTACCTAGGA
TATGTTAAATGTATAGCTGGCACCTTAGTTGCATGACCACATAGAACATGTGTCTAT
CTGCTTTTGCCTACGTGACAACACAAATTTGGGAGGGTGAGACACTGCACAGAAGA
CAGCAGCAAGTGTGCTGGCCTCTCTGACATATGCTAACCCCCAAATACTCTGAATTT
GGAGTCTGACTGTGCCCAAGTGGGTCCAAGTGGCTGTGACATCTACGTATGGCTCCA
CACCTCCAATGCTGCCTGGGAGCCAGGGTGAGAGTCTGGGTCCAGGCCTGGCCATG
TGGCCCTCCAGTGTATGAGAGGGCCCTGCCTGCTGCATCTTTTCTGTTGCCCCATCCA
CCGCCAGCTTCCCTTCACTCCCCTATCCCATTCTCCCTCTCAAGGCAGGGGTCATAGA
TCCTAAGCCATAAAATAAATTTTATTCCAAAATAACAAAATAAATAATCTACTGTAC
ACAATCTGAAAA
Blue: NAB2 exons 1-5
Green: STAT6 exons 16-22
Red: ASO IONIS993523 target site

Table S5. INT-SFT NAB2exon6-NAB2intron6-STAT6exon16 mRNA transcript sequence:

AGACAGAGGCGCGGAGGCTCGGAGAGAGAAGACGTGGAGGGAGGGACAGAGCCTG
GACAGCGGTGGACACGGCATCGTGCGCGGGGAAGAGGGCAGCACGCAGCAGGCGC
CGAGCGCCGGGCACCGAGAAGGGCAGCCCGGGTGATCTCCGGCCGTCCATGCACAG
AGCGCCTTCCCCCACAGCCGAGCAGCCGCCGGGCGGAGGGGACAGCGCCCGCCGGA
CCCTGCAGCCCAGACTCAAGCCCAGTGCCCGAGCCATGGCACTGCCTCGGACGCTG
GGGGAGCTGCAGCTGTACCGGGTCCTGCAGCGCGCCAACCTCCTTTCCTACTATGAG
ACCTTCATCCAGCAGGGAGGGGACGACGTGCAGCAGCTGTGTGAGGCGGGTGAGGA
GGAGTTTCTGGAGATCATGGCACTTGTGGGCATGGCCACCAAGCCCCTCCATGTCCG
GCGCCTGCAGAAGGCACTGAGAGAGTGGGCCACCAATCCAGGGCTCTTCAGTCAAC
CAGTGCCTGCTGTTCCCGTCTCCAGCATCCCGCTCTTCAAGATCTCTGAGACTGCGG
GTACCCGGAAAGGGAGCATGAGCAATGGGCATGGCAGCCCAGGGGAAAAGGCAGG
CAGTGCCCGCAGTTTTAGCCCCAAGAGCCCCCTTGAACTTGGAGAGAAGCTATCACC
ACTGCCTGGGGGACCTGGGGCAGGGGACCCCCGGATCTGGCCAGGCCGGAGCACTC
CAGAGTCGGACGTTGGGGCAGGAGGAGAAGAGGAGGCTGGCTCGCCCCCCTTCTCC
CCCCCTGCAGGGGGAGGAGTCCCTGAGGGGACTGGGGCTGGGGGGCTGGCAGCAG
GTGGGACTGGGGGTGGTCCAGACCGACTGGAGCCAGAGATGGTACGCATGGTGGTG
GAAAGTGTGGAGAGGATCTTCCGGAGCTTCCCAAGGGGGGATGCTGGGGAGGTCAC
ATCCCTGCTAAAGCTGAATAAGAAGCTGGCACGGAGCGTTGGGCACATCTTTGAGA
TGGATGATAATGACAGCCAGAAGGAAGAGGAGATCCGCAAATACAGCATCATCTAT
GGCCGTTTCGACTCTAAGCGGCGGGAGGGCAAGCAGCTCAGCCTGCACGAGCTCAC
CATCAACGAGGCTGCTGCCCAGTTCTGCATGAGGGACAACACGCTCTTATTACGGAG
AGTGGAGCTCTTCTCTTTGTCCCGCCAAGTAGCCCGAGAGAGCACCTACTTGTCCTC
CTTGAAGGGCTCCAGGCTTCACCCTGAAGAACTGGGAGGCCCTCCACTGAAGAAGC
TGAAACAAGAGGTTGGAGAACAGAGTCACCCTGAAATCCAGCAGCCTCCCCCAGGC
CCTGAGTCCTATGTACCCCCATACCGCCCCAGCCTGGAGGAGGACAGCGCCAGCCT
GTCTGGGGAGAGTCTGGATGGACATTTGCAGGCTGTGGGGTCATGTCCAAGGCTGA
CGCCGCCCCCTGCTGACCTGCCTCTGGCATTGCCAGCCCATGGGCTATGGAGCCGAC
ACATCCTGCAGCAGACACTGATGGACGAGGGGCTGCGGCTCGCCCGCCTCGTCTCCC
ACGACCGCGTGGGCCGCCTCAGCCCCTGTGTGCCTGCGAAGCCACCTCTCGCAGCTC
CTGACAGAACCAGAAATTCCAGCGCAACCGAGTTGCAGCTCAAAAATCTCTATCCC
AAGAAGCCCAAGGATGAGGCTTTCCGGAGCCACTACAAGCCTGAACAGATGGGTAA
GGATGGCAGGGGTTATGTCCCAGCTACCATCAAGATGACCGTGGAAAGGACCAAC
CACTTCCTACCCCAGAGCTCCAGATGCCTACCATGGTGCCTTCTTATGACCTTGGAA
TGGCCCCTGATTCCTCCATGAGCATGCAGCTTGGCCCAGATATGGTGCCCCAGGTGT
ACCCACCACACTCTCACTCCATCCCCCCGTATCAAGGCCTCTCCCCAGAAGAATCAG
TCAACGTGTTGTCAGCCTTCCAGGAGCCTCACCTGCAGATGCCCCCCAGCCTGGGCC
AGATGAGCCTGCCCTTTGACCAGCCTCACCCCCAGGGCCTGCTGCCGTGCCAGCCTC
AGGAGCATGCTGTGTCCAGCCCTGACCCCCTGCTCTGCTCAGATGTGACCATGGTGG
AAGACAGCTGCCTGAGCCAGCCAGTGACAGCGTTTCCTCAGGGCACTTGGATTGGTG
AAGACATATTCCCTCCTCTGCTGCCTCCCACTGAACAGGACCTCACTAAGCTTCTCCT
GGAGGGGCAAGGGGAGTCGGGGGGAGGGTCCTTGGGGGCACAGCCCCTCCTGCAG
CCCTCCCACTATGGGCAATCTGGGATCTCAATGTCCCACATGGACCTAAGGGCCAAC
CCCAGTTGGTGATCCCAGCTGGAGGGAGAACCCAAAGAGACAGCTCTTCTACTACC
CCCACAGACCTGCTCTGGACACTTGCTCATGCCCTGCCAAGCAGCAGATGGGGAGG

GTGCCCTCCTATCCCCACCTACTCCTGGGTCAGGAGGAAAAGACTAACAGGAGAAT
GCACAGTGGGTGGAGCCAATCCACTCCTTCCTTTCTATCATTCCCCTGCCCACCTCCT
TCCAGCACTGACTGGAAGGGAAGTTCAGGCTCTGAGACACACCCCAACATGCCTGC
ACCTGCAGCGCGCACACGCACGCACACACACATACAGAGCTCTCTGAGGGTGATGG
GGCTGAGCAGGAGGGGGGCTGGGTAAGAGCACAGGTTAGGGCATGGAAGGCTTCTC
CGCCCATTCTGACCCAGGGCCTAGGACGGATAGGCAGGAACATACAGACACATTTA
CACTAGAGGCCAGGGATAGAGGATATTGGGTCTCAGCCCTAGGGGAATGGGAAGCA
GCTCAAGGGACCCTGGGTGGGAGCATAGGAGGGGTCTGGACATGTGGTTACTAGTA
CAGGTTTTGCCCTGATTAAAAAATCTCCCAAAGCCCCAAATTCCTGTTAGCCAGGTG
GAGGCTTCTGATACGTGTATGAGACTATGCAAAAGTACAAGGGCTGAGATTCTTCGT
GTATAGCTGTGTGAACGTGTATGTACCTAGGATATGTTAAATGTATAGCTGGCACCT
TAGTTGCATGACCACATAGAACATGTGTCTATCTGCTTTTGCCTACGTGACAACACA
AATTTGGGAGGGTGAGACACTGCACAGAAGACAGCAGCAAGTGTGCTGGCCTCTCT
GACATATGCTAACCCCCAAATACTCTGAATTTGGAGTCTGACTGTGCCCAAGTGGGT
CCAAGTGGCTGTGACATCTACGTATGGCTCCACACCTCCAATGCTGCCTGGGAGCCA
GGGTGAGAGTCTGGGTCCAGGCCTGGCCATGTGGCCCTCCAGTGTATGAGAGGGCC
CTGCCTGCTGCATCTTTTCTGTTGCCCCATCCACCGCCAGCTTCCCTTCACTCCCCTA
TCCCATTCTCCCTCTCAAGGCAGGGGTCATAGATCCTAAGCCATAAAATAAATTTTA
TTCCAAAATAACAAAATAAATAATCTACTGTACACAATCTGAAAA

Blue: NAB2 exons 1-6
Purple: NAB2 intron 6
Green: STAT6 exons 16-22
Red: ASO IONIS993523 target site

Table S6. IEC139 NAB2exon6-STAT6exon16 mRNA transcript sequence:

AGACAGAGGCGCGGAGGCTCGGAGAGAGAAGACGTGGAGGGAGGGACAGAGCCTG
GACAGCGGTGGACACGGCATCGTGCGCGGGGAAGAGGGCAGCACGCAGCAGGCGC
CGAGCGCCGGGCACCGAGAAGGGCAGCCCGGGTGATCTCCGGCCGTCCATGCACAG
AGCGCCTTCCCCCACAGCCGAGCAGCCGCCGGGCGGAGGGGACAGCGCCCGCCGGA
CCCTGCAGCCCAGACTCAAGCCCAGTGCCCGAGCCATGGCACTGCCTCGGACGCTG
GGGGAGCTGCAGCTGTACCGGGTCCTGCAGCGCGCCAACCTCCTTTCCTACTATGAG
ACCTTCATCCAGCAGGGAGGGGACGACGTGCAGCAGCTGTGTGAGGCGGGTGAGGA
GGAGTTTCTGGAGATCATGGCACTTGTGGGCATGGCCACCAAGCCCCTCCATGTCCG
GCGCCTGCAGAAGGCACTGAGAGAGTGGGCCACCAATCCAGGGCTCTTCAGTCAAC
CAGTGCCTGCTGTTCCCGTCTCCAGCATCCCGCTCTTCAAGATCTCTGAGACTGCGG
GTACCCGGAAAGGGAGCATGAGCAATGGGCATGGCAGCCCAGGGGAAAAGGCAGG
CAGTGCCCGCAGTTTTAGCCCCAAGAGCCCCCTTGAACTTGGAGAGAAGCTATCACC
ACTGCCTGGGGGACCTGGGGCAGGGGACCCCCGGATCTGGCCAGGCCGGAGCACTC
CAGAGTCGGACGTTGGGGCAGGAGGAGAAGAGGAGGCTGGCTCGCCCCCCTTCTCC
CCCCCTGCAGGGGGAGGAGTCCCTGAGGGGACTGGGGCTGGGGGGCTGGCAGCAG
GTGGGACTGGGGGTGGTCCAGACCGACTGGAGCCAGAGATGGTACGCATGGTGGTG
GAAAGTGTGGAGAGGATCTTCCGGAGCTTCCCAAGGGGGGATGCTGGGGAGGTCAC
ATCCCTGCTAAAGCTGAATAAGAAGCTGGCACGGAGCGTTGGGCACATCTTTGAGA
TGGATGATAATGACAGCCAGAAGGAAGAGGAGATCCGCAAATACAGCATCATCTAT
GGCCGTTTCGACTCTAAGCGGCGGGAGGGCAAGCAGCTCAGCCTGCACGAGCTCAC
CATCAACGAGGCTGCTGCCCAGTTCTGCATGAGGGACAACACGCTCTTATTACGGAG
AGTGGAGCTCTTCTCTTTGTCCCGCCAAGTAGCCCGAGAGAGCACCTACTTGTCCTC
CTTGAAGGGCTCCAGGCTTCACCCTGAAGAACTGGGAGGCCCTCCACTGAAGAAGC
TGAAACAAGAGGTTGGAGAACAGAGTCACCCTGAAATCCAGCAGCCTCCCCCAGGC
CCTGAGTCCTATGTACCCCCATACCGCCCCAGCCTGGAGGAGGACAGCGCCAGCCT
GTCTGGGGAGAGTCTGGATGGACATTTGCAGGCTGTGGGGTCATGTCCAAGGCTGA
CGCCGCCCCCTGCTGACCTGCCTCTGGCATTGCCAGCCCATGGGCTATGGAGCCGAC
ACATCCTGCAGCAGACACTGATGGACGAGGGGCTGCGGCTCGCCCGCCTCGTCTCCC
ACGACCGCGTGGGCCGCCTCAGCCCCTGTGTGCCTGCGAAGCCACCTCTCGCAGGCT
CTCCACAGATAGAGAACATCCAGCCATTCTCTGCCAAAGACCTGTCCATTCGCTCAC
TGGGGGACCGAATCCGGGATCTTGCTCAGCTCAAAAATCTCTATCCCAAGAAGCCC
AAGGATGAGGCTTTCCGGAGCCACTACAAGCCTGAACAGATGGGTAAGGATGGCAG
GGGTTATGTCCCAGCTACCATCAAGATGACCGTGGAAAGGGACCAACCACTTCCTA
CCCCAGAGCTCCAGATGCCTACCATGGTGCCTTCTTATGACCTTGGAATGGCCCCTG
ATTCCTCCATGAGCATGCAGCTTGGCCCAGATATGGTGCCCCAGGTGTACCCACCAC
ACTCTCACTCCATCCCCCCGTATCAAGGCCTCTCCCCAGAAGAATCAGTCAACGTGT
TGTCAGCCTTCCAGGAGCCTCACCTGCAGATGCCCCCCAGCCTGGGCCAGATGAGCC
TGCCCTTTGACCAGCCTCACCCCCAGGGCCTGCTGCCGTGCCAGCCTCAGGAGCATG
CTGTGTCCAGCCCTGACCCCCTGCTCTGCTCAGATGTGACCATGGTGGAAGACAGCT
GCCTGAGCCAGCCAGTGACAGCGTTTCCTCAGGGCACTTGGATTGGTGAAGACATAT
TCCCTCCTCTGCTGCCTCCCACTGAACAGGACCTCACTAAGCTTCTCCTGGAGGGGC
AAGGGGAGTCGGGGGGAGGGTCCTTGGGGGCACAGCCCCTCCTGCAGCCCTCCCAC
TATGGGCAATCTGGGATCTCAATGTCCCACATGGACCTAAGGGCCAACCCCAGTTGG
TGATCCCAGCTGGAGGGAGAACCCAAAGAGACAGCTCTTCTACTACCCCCACAGAC

CTGCTCTGGACACTTGCTCATGCCCTGCCAAGCAGCAGATGGGGAGGGTGCCCTCCT
ATCCCCACCTACTCCTGGGTCAGGAGGAAAAGACTAACAGGAGAATGCACAGTGGG
TGGAGCCAATCCACTCCTTCCTTTCTATCATTCCCCTGCCCACCTCCTTCCAGCACTG
ACTGGAAGGGAAGTTCAGGCTCTGAGACACACCCCAACATGCCTGCACCTGCAGCG
CGCACACGCACGCACACACACATACAGAGCTCTCTGAGGGTGATGGGGCTGAGCAG
GAGGGGGGCTGGGTAAGAGCACAGGTTAGGGCATGGAAGGCTTCTCCGCCCATTCT
GACCCAGGGCCTAGGACGGATAGGCAGGAACATACAGACACATTTACACTAGAGGC
CAGGGATAGAGGATATTGGGTCTCAGCCCTAGGGGAATGGGAAGCAGCTCAAGGGA
CCCTGGGTGGGAGCATAGGAGGGGTCTGGACATGTGGTTACTAGTACAGGTTTTGCC
CTGATTAAAAAATCTCCCAAAGCCCCAAATTCCTGTTAGCCAGGTGGAGGCTTCTGA
TACGTGTATGAGACTATGCAAAAGTACAAGGGCTGAGATTCTTCGTGTATAGCTGTG
TGAACGTGTATGTACCTAGGATATGTTAAATGTATAGCTGGCACCTTAGTTGCATGA
CCACATAGAACATGTGTCTATCTGCTTTTGCCTACGTGACAACACAAATTTGGGAGG
GTGAGACACTGCACAGAAGACAGCAGCAAGTGTGCTGGCCTCTCTGACATATGCTA
ACCCCCAAATACTCTGAATTTGGAGTCTGACTGTGCCCAAGTGGGTCCAAGTGGCTG
TGACATCTACGTATGGCTCCACACCTCCAATGCTGCCTGGGAGCCAGGGTGAGAGTC
TGGGTCCAGGCCTGGCCATGTGGCCCTCCAGTGTATGAGAGGGCCCTGCCTGCTGCA
TCTTTTCTGTTGCCCCATCCACCGCCAGCTTCCCTTCACTCCCCTATCCCATTCTCCCT
CTCAAGGCAGGGGTCATAGATCCTAAGCCATAAAATAAATTTTATTCCAAAATAACA
AAATAAATAATCTACTGTACACAATCTGAAAA

Blue: NAB2 exons 1-6
Green: STAT6 exons 16-22
Red: ASO IONIS993523 target site
